# Materials Engineering for High Performance and Durability Proton Exchange Membrane Water Electrolyzers

**DOI:** 10.1021/acsaem.5c01989

**Published:** 2025-09-11

**Authors:** Pablo A. García-Salaberri, Lonneke van Eijk, William Bangay, Kara J. Ferner, Mee H. Ha, Michael Moore, Ivan Perea, Ahmet Kusoglu, Marc Secanell, Prodip K. Das, Nausir Firas, Svitlana Pylypenko, Melissa Novy, Michael Yandrasits, Suvash C. Saha, Ali Bayat, Shawn Litster, Iryna V. Zenyuk

**Affiliations:** † Department of Chemical and Environmental Technology (ESCET), 16776 Universidad Rey Juan Carlos, C/Tulipán s/n, 28933 Móstoles, Madrid, Spain; ‡ Instituto de Investigación de Tecnologías para la Sostenibilidad, 16776 Universidad Rey Juan Carlos, C/Tulipán s/n, 28933 Móstoles, Spain; § Chemistry Department, 3557 Colorado School of Mines, 1500 Illinois St, Golden, Colorado 80401, United States; ∥ Johnson Matthey Technology Centre, Blounts Court Rd, Sonning Common, Reading RG4 9NH, U.K.; ⊥ Department of Mechanical Engineering, Carnegie Mellon University, Pittsburgh, Pennsylvania 15213, United States; # Energy Systems Design Laboratory, Department of Mechanical Engineering, University of Alberta, Edmonton T6G 2R3, Canada; ∇ Energy Conversion Group, Energy Technologies Area, Lawrence Berkeley National Laboratory, Berkeley, California 94720, United States; ○ School of Engineering, Newcastle University, Newcastle Upon Tyne NE1 7RU, U.K.; ◆ School of Engineering, University of Edinburgh, Edinburgh EH9 3FB, U.K.; ¶ Department of Chemical and Biomolecular Engineering, National Fuel Cell Research Center, University of California Irvine, Irvine, California 92697, United States; ◁ Chemistry and Nanoscience Center, National Renewable Energy Laboratory, 15013 Denver West Parkway, Golden, Colorado 80401, United States; ◀ School of Mechanical and Mechatronic Engineering, University of Technology Sydney, Broadway, NSW 2007, Australia

**Keywords:** materials, design, performance, durability, characterization, modeling, PEMWE

## Abstract

Proton exchange membrane water electrolyzers (PEMWEs) are expected to play a crucial role in the global green energy transition during the 21st century. They provide a versatile and sustainable solution for generating hydrogen with very high purity in combination with renewable energies, such as solar and wind. Despite their promise, PEMWEs face several critical problems, including high costs, performance limitations, and durability challenges, particularly at low iridium (Ir) loading on the anode. Advancing next-generation PEMWEs requires extensive work on materials engineering of all cell components, including the catalyst layer (CL), membrane, porous transport layer (PTL), bipolar plate (BPP), and gasket. This task must be performed with the complementary contribution of different modeling and characterization techniques. This review presents a critical perspective from academia, research centers, and industry, mapping main developments, remaining gaps, and strategic pathways to advance PEMWE technology. A focus is devoted to key aspects, such as operation at low Ir loading, membrane durability, multiscale transport layers, porous and non-porous flow fields, multiphysics modeling, and multipurpose characterization techniques, which are thoroughly discussed. By unifying these topics, this review provides readers with the essential knowledge to grasp current developments and tackle tomorrow’s challenges in PEMWE engineering.

## Graphical abstract

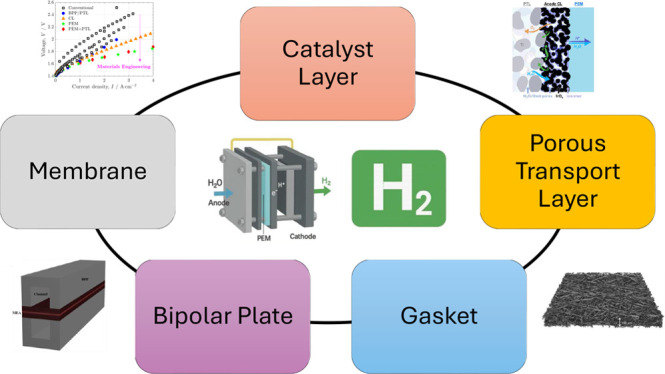

## 1 Introduction

Governments around the world have committed to decarbonizing their economies by 2050. In this endeavor, the use of hydrogen as an energy vector and feedstock in industrial applications and fine chemical production is expected to play a paramount role in combination with renewable energies, such as solar, wind, hydraulic, or geothermal.^1–4^ The worldwide transition of the energy infrastructure toward hydrogen energy necessitates the development of efficient hydrogen production, storage, and utilization. Water electrolysis technologies are crucial for sustainable green hydrogen production.^5,6^ Currently, the hydrogen energy industry has received substantial attention, with relevant policies continuously improving to create a favorable environment for technology development. From the market perspective, although the number of completed water electrolyzer projects is still limited, the capacity under construction and planning is vast. It is expected that a significant number of new projects will begin within the next few years, and the market scale is likely to expand rapidly.^7^ At the same time, more companies are actively engaging in the field, creating fierce competition and a dynamic industry environment,^8^ which presents more opportunities for the widespread application and promotion of electrolyzers with the increasing global focus on green and low-carbon development. The transition from gray to green hydrogen is crucial for achieving global decarbonization goals and creating a sustainable energy future.^9^ Unlike gray hydrogen, which is produced from fossil fuels and generates significant carbon emissions, green hydrogen is made using renewable energy sources like wind or solar power through electrolysis, resulting in zero direct CO_2_ emissions. This makes green hydrogen a clean and sustainable energy carrier that supports global efforts to reduce greenhouse gas emissions and combat climate change.^10^ Additionally, it enhances energy security by reducing dependence on imported fossil fuels and benefits from growing policy support, including subsidies and climate-focused regulations.^11^

There are three main types of water electrolyzers that operate at low temperature: liquid alkaline water electrolyzers (LAWEs), proton-exchange membrane water electrolyzers (PEMWEs), and anion-exchange membrane water electrolyzers (AEMWEs). Currently, there is also a growing interest in solid oxide electrolyzers (SOEs) operating at high temperatures, even though they are still in an early stage of development. Each type of electrolyzer drives the same reaction of splitting water into hydrogen and oxygen using an electric current, but operates under different conditions and requires distinct materials (see Table 1).^12^ The LAWE is the most mature and cost-effective electrolyzer. However, it faces challenges such as low current densities, long start-up times, and large system dimensions. The AEMWE and the PEMWE offer potentially similar operational characteristics, the former having the advantage of lower consumption of precious metals. However, state-of-the-art AEMWEs still operate at lower current densities than PEMWEs, leading to rather large electrolysis systems. Moreover, the durability of AEMWEs is a continuous source of research, especially regarding the stability of the membrane material.^13^ The larger current densities, increased durability, and compact stacks that offer the PEMWE technology make it the most promising electrolyzer type to date, even though it is critical to reduce cost in the near term.

**Table 1:** Comparison of the Main Characteristics of Different Types of Electrolyzers (LAWE, AEMWE, PEMWE and SOE), According to Their Operating Temperature, Current Density Range, Start-Up Time, Durability, Dimensions, Capital Expenditures (CAPEX) and Maturity (TRL)

parameter	liquid alkaline water electrolyzer (LAWE)	anion exchange membrane water electrolyzer (AEMWE)	proton exchange membrane water electrolyzer (PEMWE)	solid oxide electrolyzer (SOE)
operating temperature	60–90 °C	40–70 °C	50–80 °C	600–850 °C
current density range	0.2–0.4 A cm^–2^	0.3–1 A cm^–2^	1–3 A cm^–2^	0.5–1.5 A cm^–2^ (or higher at high *T*)
start-up time	minutes to hours	seconds to minutes	seconds to minutes	several hours (thermal ramp-up)
durability	>60,000 h	10,000–20,000 h	20,000–50,000 h	<20,000 h (often lower)
dimensions	large (bulky with external tanks)	moderate (still evolving)	compact (high power density)	large and complex (thermal insulation)
CAPEX	low (maturity and simple materials)	moderate-low (not standardized)	high (expensive materials)	high (expensive and complex)
maturity (TRL)	high (TRL 9–commercial)	medium (TRL 5/7–pilot)	high (TRL 8/9-commercial)	low-medium (TRL 4/6-demo)

The high efficiency of PEMWEs, particularly at small scales and under variable power inputs, is well suited for decentralized hydrogen production and use with intermittent renewable energy sources. One of its standout features is its ability to rapidly respond to changes in power supply, allowing it to seamlessly integrate with intermittent energy sources, such as wind and solar power.^14^ Additionally, the proton-exchange membrane (PEM) ensures that the hydrogen produced is of very high purity.^15^ The compact and lightweight design of PEMWE systems further enhances their appeal for decentralized or mobile hydrogen production. The advanced development of PEMWEs toward commercialization and the need in materials engineering to reduce costs while preserving or improving performance and durability motivates this work. The industrial interest in PEMWE technology is clearly reflected in Table 2, which shows a comparison of major commercial PEMWE manufacturers in the world.

**Table 2:** Comparison of Major Commercial PEMWE System Manufacturers, Indicating Their Country, Capacity, Hydrogen Output, Operating Pressure, Efficiency Based on the Higher Heating Value (HHV), and Notes of Interest

manufacturer	country	capacity	hydrogen output Nm^3^ h^–1^	operating pressure bar	efficiency (HHV) %	notes
Nel Hydrogen	Norway	up to 5 MW	∼10 to 1,000	up to 30	∼65 to 70	gigafactory
ITM Power	UK	2 MW/module	∼40	∼30	∼65 to 68	REFHYNE (10 MW)
Siemens Energy	Germany	17.5 MW/module	∼340	∼30	∼65 to 70	multiple EU projects
Cummins (Hydrogenics)	USA/Canada	1–5 MW	∼20 to 100	up to 30	∼65 to 69	projects in USA, Europe, and Canada
Plug Power	USA	1–5 MW	∼20 to 100	∼20 to 30	∼65 to 68	green H_2_ plants in U.S.
Ohmium	USA/India	1–2 MW	∼20 to 50	∼30	∼65 to 70	deployed in India, Oman, UAE
H-TEC Systems	Germany	1–2 MW	∼20 to 40	∼30	∼65	backed by MAN Energy Solutions
SinoHy Energy (Beijing SinoHytec)	China	1–2 MW	∼20 to 40	up to 35	∼65	China’s largest PEM producer
Sungrow Hydrogen	China	1–4 MW	∼40 to 160	∼30	∼65 to 68	linked to solar- H_2_ integration
PERIC Hydrogen	China	1–2 MW	∼1 to 40	∼20 to 35	∼62 to 65	electrolyzers for Chinese H_2_ stations

Although the general mechanism is similar to all electrolyzers, PEMWE systems use a solid PEM that separates the two electrodes, the anode and the cathode. Water molecules at the anode are split into protons (H^+^), electrons (e^–^), and oxygen gas when an electric current is applied, better known as the oxygen evolution reaction (OER). The protons then pass through the PEM to the cathode, where they combine with electrons to form hydrogen gas, better known as the hydrogen evolution reaction (HER). This unique membrane design is one of the key differentiators of PEMWE, offering advantages in efficiency and hydrogen purity.^16^

$$H_{2}O(l)\to 2H^{+}+2e^{-}+\frac{1}{2}O_{2}(g)$$ 1a

$$2H^{+}+2e^{-}\to H_{2}(g)$$ 1b

As shown in Figure 1, a PEMWE consists of several critical components, prominently featuring the membrane electrode assembly (MEA), a three-layered structure with the PEM at the cell’s center. The layout is similar to that of proton-exchange membrane fuel cells (PEMFCs). The PEM is typically made with a perfluorinated sulfonic acid (PFSA) polymer, such as Nafion, which conducts protons while blocking the passage of gases like hydrogen and oxygen.^17^ This feature ensures that the gases produced remain separated, enhancing the purity of the hydrogen output. The membrane is critical to ensuring the reliability and performance of PEMWE, as it allows the system to produce hydrogen with a purity level exceeding 99.99%, which is essential for many high-demand applications.^15^ The MEA also contains two electrodes, a cathodic and an anodic porous transport layer (PTL) and catalyst layer (CL). The CLs in PEMWEs are often made with noble metal catalysts, such as platinum (Pt) at the cathode and iridium (Ir) at the anode, to help facilitate electrochemical reactions during electrolysis. While these materials are more expensive than those used in alkaline systems, they are essential for achieving the high efficiency and fast response times that PEMWEs are known for. The harsh operating conditions on the anode side, characterized by high potentials and low pH, necessitate the use of these precious metals to ensure performance and durability. Each layer significantly impacts the PEMWE’s cost, performance, and durability, leading to extensive research on their properties individually and collectively as an MEA.^16,18^

**Figure 1:**
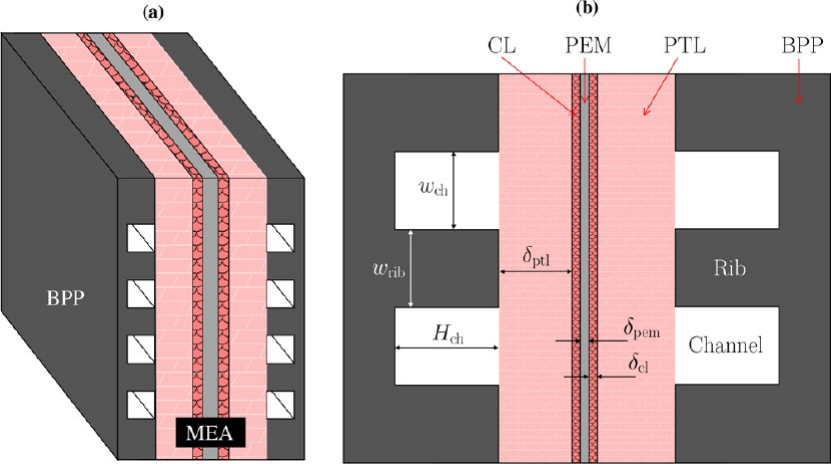
(a) Schematic of the planar structure of a cell within a PEMWE stack, indicating the location of the bipolar plates (BPPs) and the membrane electrode assembly (MEA). (b) Magnification of the cross-section of a PEMWE cell with the geometry of its main components: BPPs, porous transport layers (PTLs), catalyst layers (CLs), and proton exchange membrane (PEM). The channel/rib pattern of the BPP is indicated. Drawing dimensions are not to scale.

The PTL is made with titanium (Ti) on the anode and carbon paper on the cathode. This layer must possess sufficient mechanical strength to support the electrolysis cell, ensuring effective electrical conductivity as it connects the bipolar plate (BPP) to the CL. Note that BPP denotes here the bipolar plate of a representative cell within a stack, even though stacks of monopolar plates are also extensively used for electrolyzers. One major challenge faced within the PEMWE is the passivation of PTL materials due to the harsh anodic conditions, which necessitates using corrosion-resistant and highly conductive materials for the anode PTL. To this end, anode PTLs are typically coated with a protective layer of Pt. The anode catalyst can be deposited onto the PEM to create a catalyst-coated membrane (CCM), which is then assembled with the PTL. Alternatively, the catalyst may be directly deposited onto the PTL to form a porous transport electrode (PTE).^15,19^ The cathodic side faces fewer material limitations and is primarily composed of a carbon gas diffusion electrode (GDE) along with a Pt-based catalyst supported on carbon paper.^20,21^ Here, the HER occurs, characterized by faster kinetics than the OER, thereby not limiting system performance. Both sides are enclosed between two BPPs, which conduct electricity between the anode and the cathode. The entire assembly is compressed between two end plates, completing the cell.^22^ These systems are typically designed as stacks of individual cells, each producing hydrogen and oxygen. PEMWEs can generate significant amounts of hydrogen when combined in a stack, making them suitable for small- and large-scale applications.

Despite their numerous advantages, PEMWEs face certain challenges. Using expensive materials, particularly catalysts and membranes, contributes to higher production and maintenance costs than LAWEs. Durability is also a concern, as the membranes and catalysts can degrade over time, mainly when operating under harsh conditions. This degradation can lead to reduced efficiency and the need for replacement parts.^24^ Moreover, PEMWEs require high-purity water to avoid contamination and damage to the membrane, which adds complexity to their operation in environments where water quality may be a concern.^25^ As shown in Figure 2a, the stack can amount to 40% of the system cost, with the rest coming from the power supplies (29%) and the balance of plant (31%).^20^ Thus, reducing the cost of PEMWE stacks is a major concern for the widespread deployment of this technology. In a stack, as shown in Figure 2b, most of the cost originates from the CCM (32%) due to the high price of Nafion-like PEMs and catalysts, especially Ir at the anode. In second place, we have the BPPs with 24*%* of the stack cost, which mainly originates from the use of Ti and precious metal coatings at the anode. The cost of the anode PTL is comparable to that of the BPPs (23%) also due to the extended use of Pt-coated Ti. The prices of the cathode PTL and other components are lower, representing around 14 and 7% of the stack cost, respectively. Therefore, primary attention must be devoted to the reduction of catalyst loading, the development of more cost-effective PEMs that can be used as an alternative to Nafion, and the affordable use of metals in the anode compartment.^20,23^

**Figure 2:**
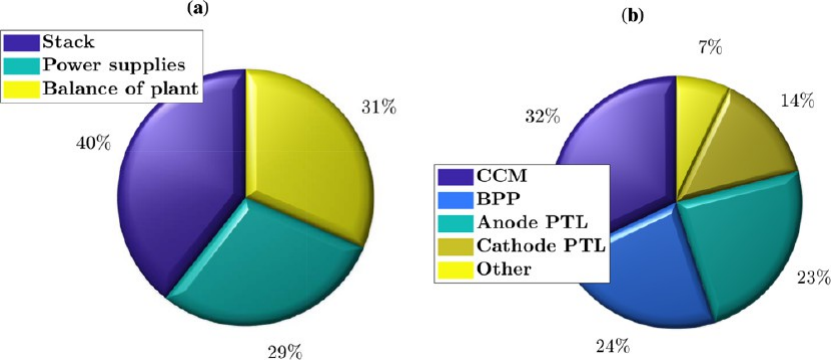
Breakdown of the cost of (a) 1 MW PEMWE system, divided into stack, power supplies and balance of plant, and (b) 5 MW PEMWE stack components, divided into CCM, BPP, anode PTL, cathode PTL and other. Data in (a) extracted from the 2019 National Renewable Energy Laboratory report based on a 50,000 units/year production rate,^20^ and in (b) from the 2021 Fraunhofer ISE report based on forecast predictions for 2030.^23^

The distributed cost and importance of the different stack components (CL, PEM, PTL and BPP) has motivated a growing body of work for their optimization. As shown in Figure 3a, the number of works published on PEMWEs has grown exponentially since the beginning of the 21st century, reaching around 500 publications per year in 2024. This trend is expected to continue in the coming years due to the worldwide concern regarding the decarbonization of the energy sector and the research needed to develop more economical PEMWE designs with high performance and durability.^26,27^ In this context, materials engineering plays a pivotal role in optimizing components with reduced cost that can make PEMWE technology more affordable, while preserving the high efficiency and lifetime demanded by energy storage applications.^28,29^ As shown in Figure 3b, this fact is clearly reflected in the number of publications that have been devoted so far to examine the CL, PEM, PTL and BPP in PEMWEs. The number of works focused on PEMWE components is inherently linked to the growth of the number of works published on this technology. This result further shows the importance of materials engineering above and beyond other technological aspects. Some current areas in materials engineering of intense research by the scientific community include but are not limited to (i) development of composite PEMs and hydrocarbon-based PEMs, as an alternative to costly PFSA membranes, such as Nafion;^30–34^ (ii) study of different CL preparation techniques, namely CCM, PTE and direct membrane deposition (DMD);^18,35,36^ (iii) reduction of Ir loading in the anode CL, either through the development of novel electro-catalysts for the OER or the improvement of triple phase point distribution at the anode PTL/CL interface;^37–42^ (iv) synthesis of alternative electro-catalysts for the HER with reduced cost and improved stability;^43–45^ (v) development of anode PTLs and BPPs with enhanced corrosion resistance and organized microstructures to promote two-phase flow, while providing good thermal and electrical conductivity;^46–48^ and (vi) design of flow-field configurations with improved reactant supply and product removal rates.^49–51^

**Figure 3:**
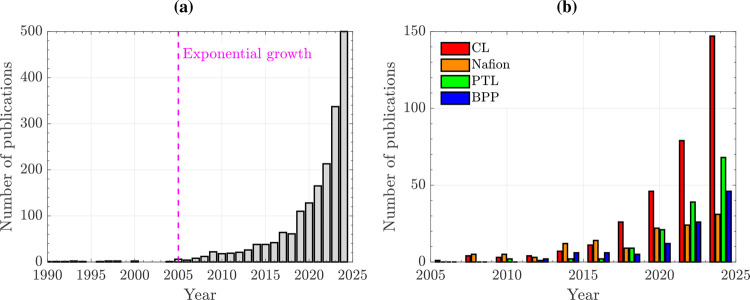
(a) Histogram of the number of publications per year with the topic “PEMWE” in the period 1990–2024. (b) Histogram of the number of publications per year with the topics “PEMWE and string”, where string is either CL, Nafion, PTL or BPP, in the period 2005–2024. Source: Web of Science.

The voltage losses in a PEMWE can be divided into three types of overpotentials (η): (i) activation overpotential due to the finite rate of electrochemical reactions, (ii) ohmic overpotential due to the finite rate of transport of electrons and protons (i.e., the limited electronic and ionic conductivities of components and interfaces), and (iii) mass concentration overpotential due to the finite rate of transport of reactants toward active catalyst sites. Figure 4a shows the overpotentials breakdown collected from various literature sources.^42,52–55^ The data set includes measurements at different cell temperatures, *T* = 50 °C^55^ and *T* = 80 °C,^42,52–54^ with different PEMs, Nafion 117 ^53,55^ (δ_pem_ = 180 μm), Nafion 115 ^42,52^ (δ_pem_ = 125 μm) and a 100 μm-thick PFSA PEM.^54^ The activation, ohmic and mass transport overpotentials can be fitted through the following expressions as a function of current density (*I*):

$$\mathrm{Activation}:\;\eta_{\mathrm{act}}=2.77\times 10^{-4}\log(3.45\times 10^{5}I)\;V$$ 2a

$$\mathrm{Ohmic}:\;\eta_{\mathrm{ohm}}=0.0046+0.13I\;V$$ 2b

$$\mathrm{Mass}:\;\eta_{\mathrm{mass}}=0.0063+0.041I\;V$$ 2c

**Figure 4:**
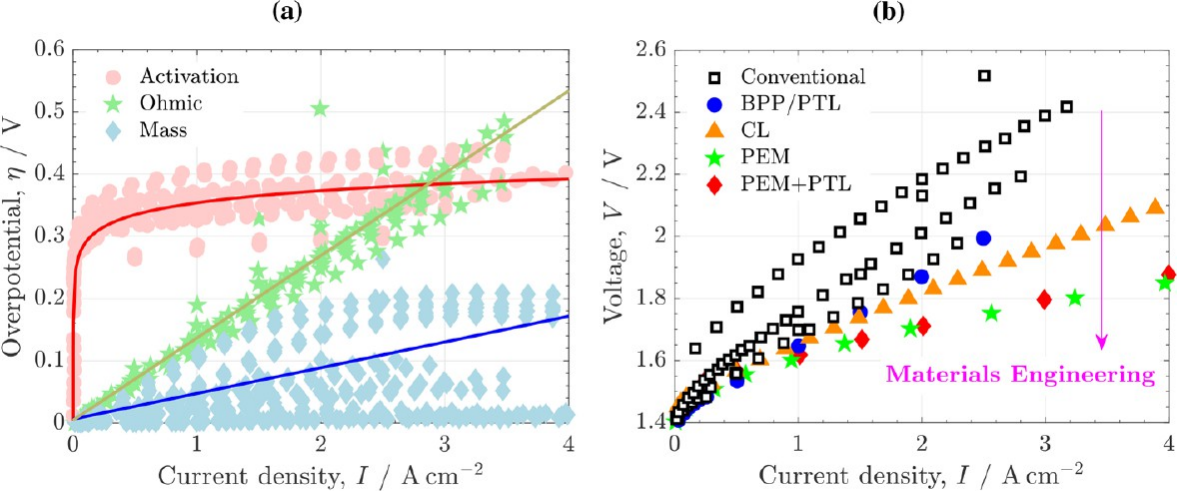
(a) Overpotentials breakdown (activation, ohmic and mass) reported by different literature sources.^42,52–55^ The fitted curves to the data sets are represented by solid lines. (b) Polarization curves reported for PEMWEs with different materials engineering improvements: conventional,^18,52,56,57^ BPP/PTL assembly,^52^ CL,^57^ PEM,^58^ and PEM+PTL.^42^ Operating temperature, *T* = 80 °C.

Note that a logarithmic expression was adopted for the activation overpotential according to the Butler–Volmer equation,^59^ and a linear expression for the ohmic overpotential according to Ohm’s law. The mass concentration overpotential was adjusted to a representative linear expression to capture the expected increase with the current density (i.e., the product generation rate, especially oxygen at the anode) and to allow a direct comparison with the ohmic overpotential.

The largest voltage losses in a PEMWE are caused by the activation and the ohmic overpotentials. The main activation overpotential is present at the anode due to the sluggishness of the OER, which is usually more than double that of the cathode (η_a_ ≈ 0.25 V vs η_c_ ≈ 0.1 V at *T* = 80 °C).^60^ The ohmic overpotential is lower than the activation overpotential at low-to-middle current densities (*I* ∼ 0 – 3 A cm^–2^) but surpasses it at higher current densities (*I* ≳ 3 A cm^–2^). The ohmic resistance largely depends on the PEM thickness, even though other sources can also be relevant, such as electrical losses at the anode PTL/CL interface at low Ir loading.^19,57,61,62^ The mass concentration overpotential is significantly less important, showing a wide variability among different works. For example, in 2019, Schuler et al.^55^ reported a non-negligible contribution of the mass concentration overpotential, especially at high current densities. The same team found in 2024 that there was a virtually zero mass concentration overpotential.^42^ The data provided by other authors lies in the middle, typically reaching around η_mass_ ≈ 0.1 V at *I* = 4 A cm^–2^. Therefore, despite the lack of a firm consensus among authors, the detrimental effect of the mass concentration overpotential should not be disregarded.

The potential benefit that can be achieved on PEMWE performance and efficiency by materials engineering is illustrated in Figure 4b. The polarization curves reported for conventional cell designs without any particular advancement^18,52,56,57^ are compared with those reached by introducing certain materials engineering improvements.^42,52,57,58^ All data were measured at *T* = 80 °C. A decrease of up to Δ*V* ≈ 0.5 V is observed at *I* = 2 A cm^–2^ between the worst conventional design and the best modified design, showing the large margin of improvement that exists for materials engineering in PEMWEs. The magnitude of the voltage enhancement is directly related to the relative importance of the affected overpotentials discussed before. The lowest improvement is found in the work of Tugirumubano et al.,^52^ who presented an innovative PTL design with integrated channels at the anode to modify the PTL/flow channel interface and provide additional pathways for in-plane flow. As a result, they reported better water transport to the region under the rib and PEM hydration, decreasing the mass concentration and ohmic overpotentials by 71.4 and 42.8%, respectively. The Ir loading was not modified with respect to that of conventional designs (*L*_Ir_ = 1.0 mg_Ir_ cm^–2^). The second lowest performance improvement is found in the work of Ferner and Litster,^57^ who proposed a composite anode CL using a cheaper conductive additive than Ir (Pt black) to facilitate robust, high-performance operation with low Ir loading (*L*_Ir_ ≈ 0.1 mg_Ir_ cm^–2^) by retaining electrode thickness and in-plane electrical conductivity. This approach allowed them to reduce the Ir loading by 95% and cost by 80% while preserving high performance and stability. The best performance improvements were reported by Lyu et al.^58^ and Schuler et al.^42^ In both works, a thin PEM with a thickness of δ_pem_ = 50 μm (Nafion 212) was introduced (compared to δ_pem_ ≈ 125–180 μm used in conventional designs), so the main origin of the exceptional performance is ascribed to the reduction of the cell’s ionic resistance. Additionally, it is worth noting that Lyu et al.^58^ used IrO_2_ doped with TiO_*x*_ and NbOx as the anode catalyst at a relatively low Ir loading of *L*_Ir_ = 0.3 mg_Ir_ cm^–2^. In the case of Schuler et al.,^42^ they also incorporated a thin Ti microporous layer (MPL) with a thickness of δ_mpl_ ≈ 50 μm to improve electrical conduction and mass transport at the anode, using an Ir loading of *L*_Ir_ = 0.4 mg_Ir_ cm^–2^. The above examples show some of the opportunities that materials engineering can bring to maintain or even improve performance while reducing the amount of expensive materials. Further research is still needed to determine the technological limits that exist in terms of Ir loading reduction, PEM thickness reduction, MPL design, or integration of the BPP and PTL porous architecture. These areas should be examined by a combination of experimental and numerical work to build up a solid foundation.

Apart from performance optimization, degradation is an ever-present challenge in PEMWEs. Almost all components are susceptible to degradation, especially in the strongly acidic environment and at the high electrochemical potentials reached on the anode side (see, e.g.,^22,63–65^ among others). Figure 5 shows a summary flowchart of some of the main degradation phenomena found in the CL, PEM, PTL, and BPP. Catalyst dissolution, oxidation, and agglomeration present a significant problem for the anode CL.^65^ The combined action of catalyst degradation leads to a reduction of the electrochemically active surface area (ECSA), electrochemical activity, electrical conductivity, and possibly ionic conductivity.^22,66^ In addition, mechanical degradation caused by the assembly pressure and cyclic PEM swelling can lead to the creation of defects and cracks in the CL, negatively affecting two-phase transport and catalyst microstructure.^67,68^ The PEM can suffer from both chemical and mechanical degradation. Chemical degradation involves membrane thinning due to attacks from hydrogen peroxide radicals, while mechanical degradation is due to changes in temperature and humidification as well as electrolyte creep under compression.^17,69–72^ The loss of electrolyte material in the sulfonated side chains and/or the backbone reduces the ionic conductivity and increases the crossover rate of gas species between compartments.^73,74^ This issue is aggravated by the possible creation of pinholes and cracks in the PEM under mechanical stressors, which can exacerbate the conductivity reduction and crossover increase, eventually leading to mechanical failure and the need for material replacement.^75–78^ Allowing the mixture of hydrogen and oxygen raises serious safety concerns due to the high flammability of hydrogen in the operating window of PEMWEs.^79^ Oxidation/passivation of the anode PTL and BPP surface is also a relevant degradation concern. The metal oxide compounds formed are characterized by low electrical conductivity, which can lead to both a reduction of the bulk electrical conductivity and a high electrical contact resistance between components (e.g., at the anode PTL/CL interface).^47,80,81^ For this reason, the anode PTL and BPP usually receive a surface treatment by adding a protective coating (e.g., Pt). However, these treatments typically involve noble metals, further increasing PEMWE cost. Surface treatments that only involve non-noble metals would be desirable, although it is currently a scientific and industrial challenge.^82^

**Figure 5:**
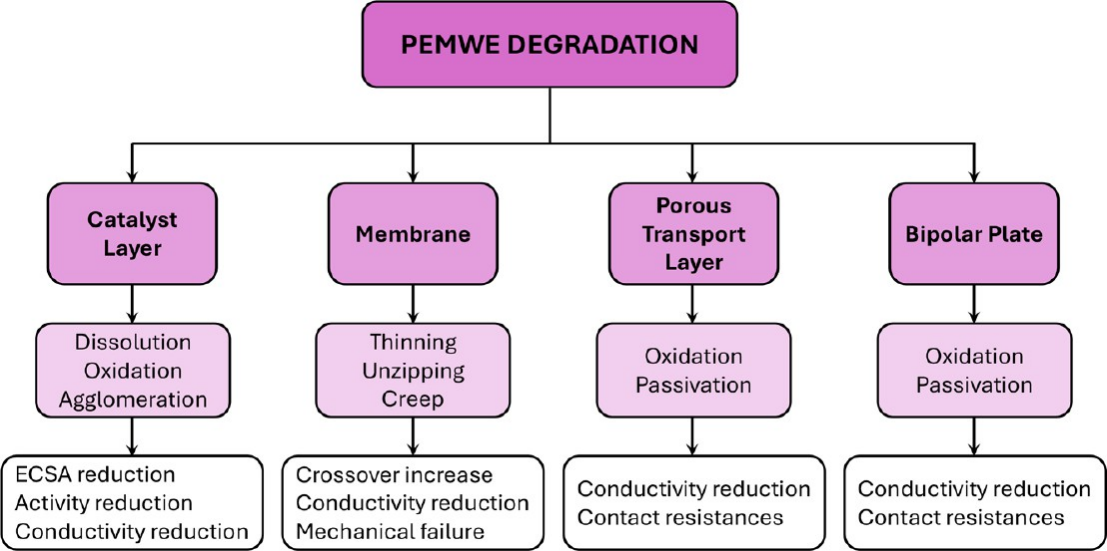
Flowchart of the main degradation phenomena that take place in the CL, membrane, PTL and BPP of a PEMWE, especially at the anode side.

Operation at high current density is also expected to amplify degradation stressors on PEMWE components. Elevated current densities accelerate OER and HER rates, leading to increased bubble formation and mechanical stresses within the CL and PTL, which can cause cracks, delamination, and loss of ECSA.^83^ Localized heating can also exacerbate membrane dehydration, mechanical thinning, and chemical degradation, while also increasing ionic resistance.^84,85^ On the anode side, intensified oxygen bubble accumulation can promote uneven current distribution and localized hot spots, thereby accelerating catalyst dissolution and support corrosion.^84^ Similarly, in the PTL and BPP, higher current densities amplify corrosion, passivation, and structural degradation, which contribute to increased interfacial resistances.^46,86^ In addition, polymeric seals and gaskets experience greater thermal and mechanical stresses under fluctuating humidity and temperature gradients, resulting in swelling, creep, and eventual mechanical failure.

The variety of degradation processes discussed above, along with the stringent requirements set by the U.S. Department of Energy (DOE) in terms of precious metals reduction and durability increase, have recently motivated a large body of work in this area.^87^ The most extensive strategies in the literature are devoted to the reduction of Ir loading and Pt content while preserving or increasing PEMWE lifetime, including the following three action lines: (i) modification of catalyst and microstructure of the anode CL;^88–93^ (ii) use of novel PEMs and the addition of radical scavengers for extended durability of PEMs based on either hydrocarbon or PFSA ionomers;^30,94,95^ (iii) development of coatings for the anode PTL and BPP without or with reduced Pt loading.^96–99^ The growth of this research trend is expected to continue in the near term, as the industrial interest for cost-effective, long-term green hydrogen storage increases in the U.S. and other leading countries.

In tandem with the ongoing work on materials durability, there is significant literature devoted to the development of accelerated stress tests (ASTs) to evaluate PEMWE degradation and durability.^64,100,101^ The lower maturity of testing protocols for PEMWEs compared to other related electrochemical technologies, such as PEMFCs, demands work for the standardized, harmonized assessment of performance and durability. In particular, further work is required to establish equivalent ASTs adapted to the long service time expected for PEMWEs under both continuous and cycling operations.^100^ Furthermore, ASTs must be adapted to analyze specific components subjected to different degradation modes (e.g., chemical vs mechanical degradation).^64^ Durability targets must be translated into physicochemical degradation descriptors, such as mass activity loss for the OER in the anode CL, carbon corrosion in the cathode CL, fluoride emissions from the PEM, or increase of internal contact resistances. These deficiencies make a one-to-one comparison between conventional and novel materials difficult, complicating the technological movement from lab scale to commercial deployment.

Significant reviews have been published on PEMWEs, especially in the last five years, either focused on a general description of the technology,^28^ overall materials and components,^22,29,102,103^ specific components (CL,^19,65,104–107^ PEM,^108^ PTL,^46,47,109,110^ BPP^82,111–113^ ), degradation,^64,76,101^ performance,^114^ both performance and degradation^63^ or other topics, such as multiphase flow and mass transport,^115,116^ and machine learning.^117^ The large amount of published work hampers the learning curve of the reader in the field, making it difficult to select a preferred baseline literature source. The aim of this review is to provide the reader with a comprehensive, actual, and exhaustive analysis of materials engineering in PEMWEs. The long-standing experience of the authors from academia, research centers, and industry is integrated into a single document with the purpose of giving a reference review to the water electrolyzer community. In particular, little work has addressed simultaneously the impact of materials engineering on performance and durability, along with a discussion of numerical modeling and characterization techniques at different scales. Including all this information in a single work is considered indispensable to get a global but detailed picture of the field. The three main objectives considered during the development of this review are summarized in the following points:

- To provide an updated review of materials engineering with application to the design of high performance and durable PEMWEs. Electrolysis technologies are rapidly evolving with an exponential growth in the number of publications during this century, so it is essential to keep up to date with new developments in the field.
- To provide a comprehensive review including the complementary perspectives provided by experts in the field with different backgrounds. The complete design of a PEMWE involves knowledge from different disciplines (e.g., chemical and mechanical engineering) and materials (e,g, catalyst, PEM and PTL/BPP design), so it is crucial to combine complementary expertise.
- To provide a critical vision of the state of the art and the main opportunities and challenges that must be overcome in the near term to advance PEMWE technology. The vast amount of work generated in the field requires a critical analysis to delineate the most important aspects to be addressed by the water electrolyzer community in the coming years.

The organization of the paper is as follows. In Sections 2, 3, 4, 5, and 6, the design of the CL, PEM, PTL, BPP and gasket is examined toward the development of high performance and durability PEMWEs, respectively. Attention is devoted to the traditional use of materials in the field and novel strategies under research. Sections 7 and 8 are devoted to a review of the state of the art in terms of mathematical modeling and characterization techniques, as complementary approaches for understanding PEMWE behavior. Finally, a summary of the review is presented in Section 9, including the main conclusions drawn from the work.

## 2 Catalyst Layer

### 2.1 Introduction

The use of catalysts in PEMWEs is necessary to facilitate both the OER at the anode and HER at the cathode, which is paramount to such systems’ performance, durability, and cost. Thus, a significant amount of research has focused on appropriate material selection for such catalysts, and large, continued efforts are needed for their optimization and improvement.^105,107,118^ At the highest level, the most basic requirement for material selection of both the anode and cathode catalysts is high kinetic activity toward the OER and HER, respectively, to enable the reactions to proceed rapidly and minimize kinetic overpotentials. Yet, arguably of equal importance is the requirement of acid stability. The high concentration of protons in flux across the PEM results in a highly acidic electrolyte environment (pH ∼ 0), and materials readily dissolved in acid will quickly degrade PEMWE cell performance. The other major limiting factor for the anode (but not the cathode) is the thermodynamically high potentials needed to drive the OER (>1.3 V vs RHE), resulting in highly oxidizing conditions. Thus, in the case of the OER anode catalysts, the only viable materials that can withstand these harsh conditions are platinum group metals (PGMs).^119–121^ Another key consideration for the anode is the sluggishness of the OER compared to the fast HER. Compared to the two-electron transfer of the HER, the four-electron transfer required for the OER results in slower kinetics at the anode. Thus, most of the cell’s kinetic overpotential is attributed to the anode rather than the cathode.^122^ Consequently, more research and literature have focused on catalysts and CL design for the OER anode. Nonetheless, achieving a good balance of activity and stability, enabling high durability, and ensuring quality electrode engineering is crucial for both OER and HER catalysts and CLs.

The activity trends of various catalyst materials toward a particular reaction can be examined by plotting the theoretical overpotential versus a descriptor of the materials’ affinity for the reaction intermediates (i.e., surface binding energy, formation enthalpy), which typically yields a “volcano plot”. Following Sabatier’s principle, the most promising catalysts are identified as the materials at the optimum of the volcano plot, which do not bind to reaction intermediates too weakly or too strongly.^118,119,123^ In conjunction with stability considerations, today’s standard PEMWE catalysts are Ir- or Ru-based materials for the anode and Pt-based materials for the cathode. Additional trends of activity and stability can be further distinguished by comparing more specific features of these materials (i.e., electronic structure, surface states, oxides, alloys, etc.), which will be discussed in the following sections.

Although activity and electrochemical stability are key factors for achieving high initial performance, the durability of the catalysts and CLs must also be considered. Durable catalysts and CLs are crucial for long-term operation and commercial relevance for PEMWE systems, that is, catalysts that will not degrade significantly over tens of thousands of hours due to chemical, electrochemical, or mechanical factors.^65,106,124^ Currently, commercial systems that must meet durability and lifetime requirements contain overengineered CLs with high loadings of PGMs.^125,126^ Given the harsher conditions and more sluggish kinetics of the OER, higher loadings of Ir are needed for the anode (1–3 mg cm^–2^), while lower loadings of Pt (≤0.1 mg cm^–2^) are typical without incurring significant performance loss.^127^ The high commercial loadings of Ir are needed for durable CLs for thicker, uniform, and mechanically robust electrodes while also buffering against Ir dissolution.

#### 2.1.1 Limitations and Outlook of PGMs in PEMWE

The high loadings of PGMs currently used for high-performance and durability CLs are among the most critical obstacles that must be overcome for wider adoption of PEMWE for cost-competitive green hydrogen. Namely, the high cost and scarcity of these PGM-based materials render the current loadings unsustainable for the increased deployment of PEMWE systems needed to meet decarbonization goals in the coming decades. As shown in Figure 6, the current nominal cost of Pt is ∼$1,000/troy oz, while the cost of Ir is around ∼$4500/troy oz.^128^ Moreover, PGMs are rare, precious metals, with an annual global mine production of approximately 200 tons of Pt and only 7–8 tons of Ir (ca. 2018).^129^ The high cost and scarcity of these materials result in the CLs being one of the most significant contributors to the overall stack cost of PEMWE systems. Furthermore, as the annual PEMWE capacity production rate increases and other stack components are subject to the benefits of economies of scale, the costs associated with the scarce PGM catalysts become even more significant toward the overall stack cost.^126^ Thus, for PEMWE systems to contribute at the GW scale to global decarbonization, three key objectives must be met: (i) total PGM loadings reduction, (ii) high current density operation/high efficiency, and (iii) low degradation rates for long lifetimes. Specifically, for example, the U.S. DOE has delineated targets of: (i) loadings of 0.125 mg cm^–2^ of total PGMs, (ii) performance of 3 A cm^–2^ at 1.6 V_cell_, and (iii) degradation rate of 2.0 μV h^–1^ with 80,000 h lifetime.^87^ The following subsections will discuss the critical processes and considerations, survey the current state-of-the-art, and review recent advancements in alternative materials, designs, and methods related to CLs, which show a promising outlook toward achieving these goals.

**Figure 6:**
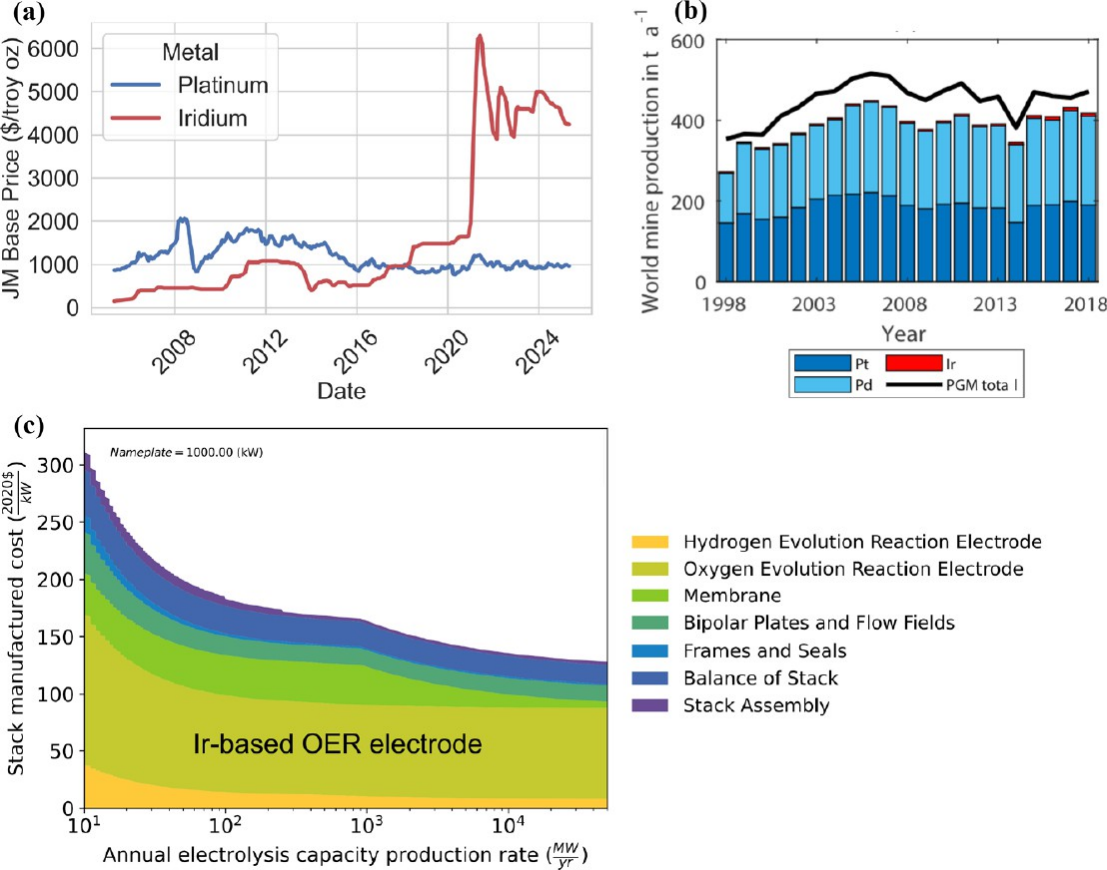
(a) Prices of Pt and Ir from 2005 to 2025. Data extracted from Matthey.^128^ Copyright 2025 Johnson Matthey. (b) World mine production of Pt, Ir, and Pd from 1998 to 2018. Figure adapted from ref 129. Copyright 2021 The Authors under Creative Commons Attribution 4.0 License (http://creativecommons.org/licenses/by/4.0/). (c) Breakdown of PEMWE stack manufactured cost vs annual production rate. Figure adapted from ref 126. Copyright 2024 National Renewable Energy Laboratory.

### 2.2 Anode CL

The anode CL typically comprises two key components: an Ir-based catalyst and a proton-conducting ionomer (which also serves as a binder). Currently, the baseline Ir-based catalyst in the field is IrO_*x*_, either amorphous or crystalline, of nanoparticle structure. The ionomer film binds these nanoparticles together, forming the porous CL typically on the order of 10 μm thick. Moreover, the CL is not self-standing; therefore, it either adheres to the membrane, forming a CCM configuration, or the PTL, creating a PTE configuration. This ionomer-bound nanoparticle CL is the current conventional PEMWE anode CL configuration. Alternative configurations, such as ionomer-free or thin film CLs,^130–133^ have emerged as promising techniques to lower Ir loading and simplify fabrication, yet are not as common or are in early stage development. Thus, this section will use the traditional ionomer-bound, Ir-based nanoparticle CL as the basis for discussion of CL structure, transport processes, fabrication, etc. While alternatives to catalyst and CL designs will be discussed throughout, it is important to note that different types of CLs can vary in these aspects.

The following subsections will delve deeper into critical materials engineering considerations for the anode CL, first discussing the complex transport phenomena that occur at the CL level (Section 2.2.1) and then the requirements for acidic OER at the catalyst material level (Section 2.2.2). Next, key strategies of alternative catalysts and CL designs for performance and cost improvement will be surveyed (Section 2.2.3), followed by a discussion of special considerations regarding degradation (Section 2.2.4), the use of ionomer (Section 2.2.5), and preparation methods for the anode CL (Section 2.2.6).

#### 2.2.1 Transport Processes in the Anode CL

In the conventional design, the anode CL is sandwiched between the PTL and the membrane and is responsible for the OER, requiring several transport processes to coincide, as shown in Figure 7. First, the liquid water reactant that is transported to the CL through the BPP flow field and the PTL pores must reach a catalyst active site. After the reaction occurs, all products must be effectively transported away from the active site: electrons must reach the PTL to then pass through the external circuit, protons must be conducted to the membrane, and oxygen gas must diffuse back through the pores of the PTL and anode flow field to be removed from the cell.^16,134^ Any anode CL configuration requires at least three phases to be accessible at the reaction sites: an electrically conducting phase, a protonically conducting phase, and a pore phase. In a typical ionomer-bound IrO_*x*_ CL, these phases are provided by the IrO_*x*_, the ionomer, and the pores, respectively. The IrO_*x*_ has decent electrical conductivity and is responsible for both in-plane and through-plane electron conduction to transport electrons to the PTL. The ionomer is the primary mode of proton conduction, and the pore phase is necessary for delivering reactant liquid water and removing product oxygen gas. Like other porous electrodes in electrochemical systems, optimizing these necessary phases and increasing the triple phase contact area (TPCA) is critical for improving overall performance for a given catalyst material.

**Figure 7:**
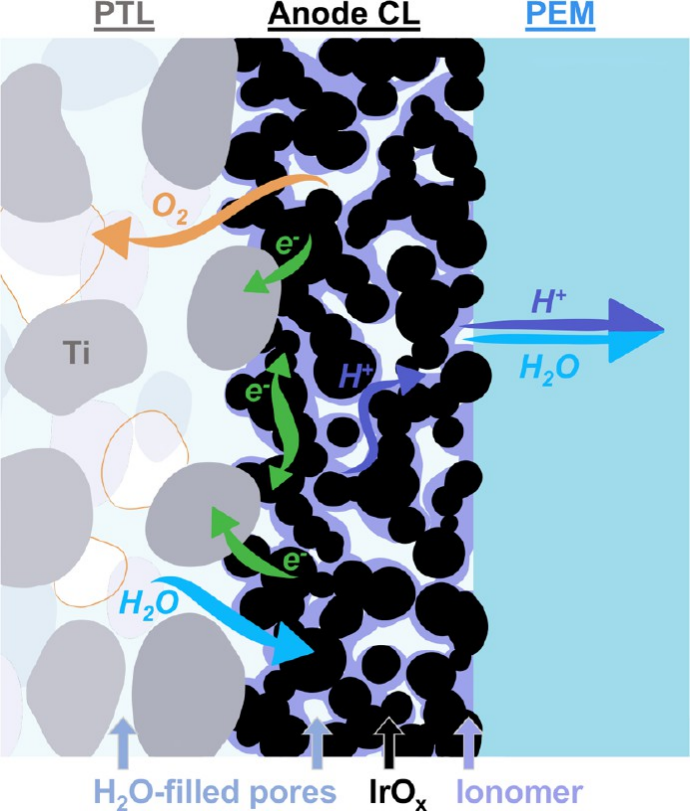
Transport processes at the anode for a conventional ionomer-bound IrO_*x*_ CL. Liquid water, transported through the pores of the PTL to the anode CL, reacts at the catalyst active sites, producing electrons, protons, and oxygen. Electrons are conducted in the in-plane direction through the IrO_*x*_ and in the through-plane direction to reach the Ti PTL. Protons are primarily transported through the ionomer network to reach the membrane, then conducted across toward the cathode. Product oxygen gas must diffuse back through the water-filled pore network of the CL and the PTL to be removed from the cell. Additionally, electroosmotic drag results in the transport of water through the membrane to the cathode.

#### 2.2.2 Acidic OER Catalysts

In addition to effectively managing the complex transport phenomena at the anode CL, careful selection of the catalyst material is of vital importance. As mentioned, PEMWE anode catalysts should exhibit high activity toward the OER and good stability in the acidic and highly oxidizing environment, limiting the best material choices to excellent and stable catalysts like PGMs. Analyses of PGMs for acidic OER have established that activity increases as Os ≫ Ru > Ir > Pt ≫ Au (in monometallic oxide form), while stability has the precise inverse relationship.^121^ Moreover, crystalline oxides are more stable yet less active than amorphous or hydrous oxides. It has also been established that metallic materials (Ir, Ru) are initially more active but less stable than their oxides (IrO_2_, RuO_2_).^135^ It has been proposed that these inverse relationships are correlative, such that the mechanisms for high activity toward OER (such as high crystalline structure defect density) contribute to higher metal dissolution, resulting in poor stability. Therefore, the best OER catalysts should balance activity and stability. Thus, far, IrO_2_ has best met these criteria and has served as the state-of-the-art catalyst for PEMWE systems for the past several years, both at the laboratory and commercial levels.^121,135^ While research on PGM-free catalysts for PEMWE anodes is prevalent, we exclude this topic from our review. Instead, we focus on Ir-based catalysts, referring readers to existing works on PGM-free catalyst discussion.^136,137^

#### 2.2.3 Strategies for High Performance Anodes with Reduced Iridium

The critical challenge of reducing Ir content while maintaining or enhancing performance and durability can be addressed through two complementary approaches: (i) enhancing the intrinsic activity of catalyst materials, and (ii) improving catalyst utilization within the electrode structure, as depicted in Figure 8. In other words, one approach is to produce better catalysts, and another is to use them more efficiently. The former relies on a material synthesis focus to create more active Ir-based catalysts, while the latter aims to structurally engineer catalyst and CL designs to maximize efficiency. This structurally focused pathway can be further categorized into distinct strategies – increasing catalyst surface area, enhancing electrical conductivity, and improving the PTL/CL interface – each addressing specific limitations in the current conventional ionomer-bound IrO_*x*_ CL toward maximizing utilization of the scarce and expensive Ir. Notably, these approaches are neither exhaustive nor mutually exclusive. Instead, the most promising advancements often combine multiple strategies to overcome the complex transport and stability challenges in anode CLs.

**Figure 8:**
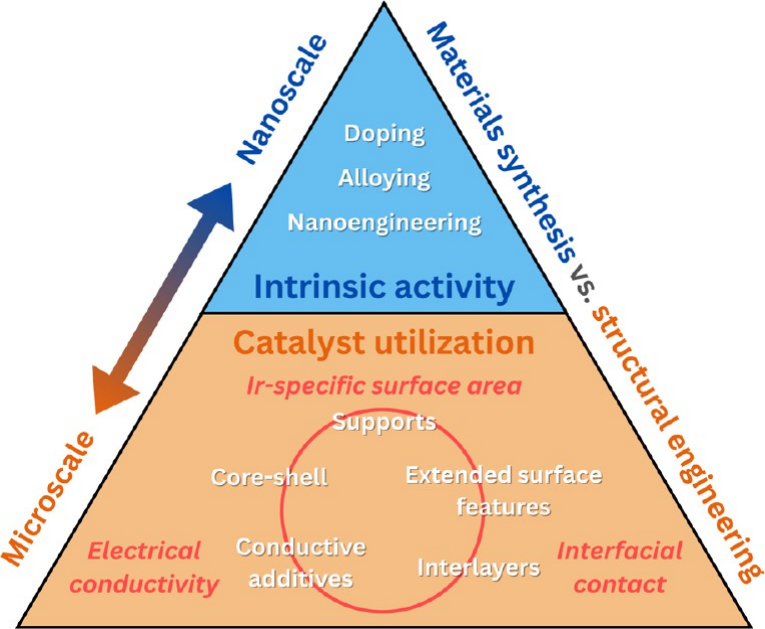
Strategies for anode CL improvements, focused on material synthesis for increasing the intrinsic OER activity of the catalyst, and on structural engineering of catalysts and CLs for improving catalyst utilization.

##### 2.2.3.1 Intrinsic OER Catalyst Activity

Catalysts with improved intrinsic activity enable more reactions per active site per unit time, or a higher turnover frequency (TOF), effectively producing more current at a given overpotential, which in turn allows for lower loadings of catalyst needed. However, TOF is challenging to quantify in practice, given the difficulty of experimentally determining the precise number of active sites. Similarly, specific activity (*j*_ECSA_ [A cm_ECSA_^–2^]), which is the current normalized to the ECSA, provides a good metric of the intrinsic catalyst performance. While specific activity is commonly used for PEMFC Pt-based electrodes, it is experimentally challenging to determine ECSA for IrO_*x*_-based electrodes. Hence, other metrics are often used to evaluate activity, such as Tafel slope, kinetic overpotential at a given current density, or current density at a given overpotential.

Strategies to improve the intrinsic activity of Ir-based catalysts have included modifying the electronic structure, atomic structure, surface coordination, and oxidation state. Numerous novel catalyst materials have been synthesized through doping, alloying, tuning nanoscale morphology, or a combination of these methods. In particular, alloys and mixed oxides containing both Ir and Ru have shown competitive outcomes compared to IrO_*x*_ catalysts.^138^ Creating IrRu alloys or multimetallic oxides of IrRuO_*x*_ can benefit from both the high activity of Ru and the superior stability of Ir. Siracusano et al.^139^ synthesized an Ir_0.7_Ru_0.3_O_*x*_ catalyst via thermal treatment and demonstrated a higher OER activity than an IrO_2_ catalyst. They also later showed good performance with Ir_0.7_Ru_0.3_O_*x*_ of nearly 3 A cm^–2^ @ 1.8 V_cell_ and a degradation rate of 23 μV h^–1^ at a low loading of 0.34 mg_Ir+Ru_ cm^–2^.^84^ Moreover, Wang et al.^140^ compared an electrochemically prepared IrRuO_*x*_ to a thermally prepared counterpart, showing a 13-fold increase in half-cell activity. Besides Ru, alloying Ir with cheaper and more abundant transition metals, such as Ni, Fe, and Co, is an area of significant ongoing research.^141–144^ For example, Alia et al.^145^ fabricated Ir–Ni nanowires, modifying the material’s electronic and morphological structure for enhanced activity. Similar to this work, many other novel high-activity catalysts employ changes both in elemental composition and nanoscale architecture. There are a vast number of similarly innovative OER catalyst materials in the literature, which have been extensively reviewed by others.^105,146,147^ However, many of these materials are still in the early stages of development. They will require additional research to demonstrate scale-up from half-cell to full-cell PEMWE systems, as well as the durability necessary to compete with conventional IrO_*x*_ catalysts.

##### 2.2.3.2 Ir-Specific Surface Area

In addition to enhancing the intrinsic activity of the catalyst, improving catalyst utilization is crucial for PEMWE performance. While intrinsic activity is challenging to quantify in real PEMWE systems, catalyst utilization can be more readily assessed using mass activity (*j*_mass_ [A mg_Ir_^–1^]), which measures the current generated per unit mass of Ir. A higher mass activity indicates more effective use of the catalyst within the CL. One key strategy to enhance utilization is increasing ECSA per unit mass of Ir, or in other words, the Ir-specific (mass-specific) surface area. Optimizing nano- and microstructural design can significantly increase ECSA and improve kinetic performance while minimizing scarce and expensive Ir.

One of the most established strategies for increasing ECSA is the use of supported catalysts, where small Ir nanoparticles are dispersed onto larger support particles to enhance the Ir-specific surface area. This approach has been successfully used for Pt/C catalysts in PEMFCs; however, PEMWE anodes cannot rely on carbon supports due to their instability under highly oxidizing conditions. Instead, acid-stable and oxidation-resistant supports such as TiN, TaC, and SnO_2_ have been explored, with TiO_2_ emerging as the most robust due to its stability, low cost, and commercial availability. Oakton et al.^148^ synthesized a 55 wt % IrO_2_/TiO_2_ catalyst with a higher surface area and improved mass activity in a rotating disk electrode (RDE) compared to unsupported IrO_2_, attributed to better Ir dispersion and accessibility. Recently, Zargarian et al.^149^ used a freeze-drying technique to synthesize homogeneous and well-dispersed IrO_2_/TiO_2_ with varying Ir loadings, finding that 40 wt % provided an optimal connectivity and performance. Commercial IrO_*x*_/TiO_2_ catalysts (75 wt % IrO_2_/TiO_2_ from Umicore and 45 wt % IrOOH/TiO_2_ from Heraeus) have also demonstrated high performance. Bernt et al.^150^ compared these materials and found that the 45 wt % amorphous catalyst exhibited nearly 37 times higher mass activity at reduced Ir loadings due to lower packing density and improved utilization. However, this benefit was only observed when using a platinized PTL, revealing that its significantly lower electrical conductivity (nearly 3 orders of magnitude lower than the 75 wt % catalyst) limits standalone performance. As such, achieving high performance at low Ir loadings with supported catalysts requires further optimization of CL thickness, Ir distribution, and, especially, electrical conductivity, which is discussed in the following section.

Other novel structural strategies have been explored to enhance ECSA and Ir-specific mass activity, including the design of high-aspect-ratio features and highly porous catalyst architectures to increase the overall surface area and active site accessibility, thereby reducing Ir content. To date, one of the best performances achieved for PEMWE systems was demonstrated with 3M’s nanostructured thin film (NSTF) catalyst, achieving 4.98 A cm^–2^ @ 1.8 V_cell_ with an Ir loading of 0.25 mg cm^–2^ and membrane thickness of 50 μm.^132^ The NSTF design features a crystalline polymer “whisker”-like foundation with an Ir coating, which effectively disperses Ir while maintaining activity and stability. Other similar designs with extended surface area features have been reported, such as nanowires^145,151,152^ or IrO_*x*_ nanotube arrays.^153^ However, further development is needed regarding durability and scale-up. Synthesizing highly porous catalysts has been demonstrated by using templates or pore-formers that are dissolved or removed to leave behind a structure with high porosity for mass transport, high ECSA for catalyst accessibility, and a CL with robust network connectivity and thickness. Examples in recent literature include using polymer beads as a template to form mesoporous IrO_2_, Ir–Ru mixed oxides, or Ir–Mo mixed oxides,^68,154–156^ an inverse-opal structure of IrO_*x*_,^157^ and honeycomb-structured IrO_*x*_ foam platelets.^158^ Similarly, dealloying Ir-based catalysts has been shown to create porous structures with improved ECSA and promising activity. In a recent study, Liang et al.^159^ synthesized porous Ir metal aerogels by dealloying IrCu, demonstrating 2 A cm^–2^ @ 1.79 V with 0.5 mg_Ir_ cm^–2^, outperforming a high-loaded Ir black anode and decaying only ≈40 μV h^–1^. Similar to intrinsic activity improvements, many of the Ir-specific surface area enhancements have shown promising activity, and in some cases, even good stability. Nevertheless, the repeatable fabrication of the catalyst materials, integration into the CL of a full PEMWE cell, and long-term durability remain key factors that need to be addressed.

##### 2.2.3.3 Electrical Conductivity

In addition to surface area effects, electrical conductivity is another crucial aspect for PEMWE anode CLs. Especially when reducing Ir loadings, in-plane electrical conductivity becomes a significant limitation.^160^ Previous studies have shown a linear relationship between Ir loading and CL thickness in conventional ionomer-bound IrO_*x*_ CLs.^161,162^ The reduced thickness at low Ir loadings exacerbates in-plane conductivity issues since IrO_*x*_ is the sole electrically conductive phase in these CLs. Additionally, fabricating homogeneous low-loading CLs without cracks or agglomeration is challenging, and these defects can further hinder in-plane electrical conductivity, overall catalyst utilization, and lead to hotspots that increase degradation.^127,134,163^

As mentioned, using supported catalysts is a promising approach to improve mass activity, yet they suffer from low electrical conductivity. Since most viable and stable support particles (e.g., TiO_2_, SnO_2_) are highly insulating, the IrO_*x*_ remains the preferential electron pathway over the support. This requires high Ir loadings, often despite performance benefits achieved due to increased Ir-specific surface area.^164–166^ To address this, core–shell architectures have emerged as a promising alternative. Ir or IrO_*x*_ forms a continuous, thin shell around a support core in these structures, maintaining electrical percolation across particles even at low Ir loadings. Several studies have shown that optimizing heat treatment and precursor chemistry can yield continuous and well-connected IrO_*x*_ shells while preserving high surface area and activity.^166–169^ For example, Böhm et al.^165^ demonstrated that thermal treatment of an IrOOH_*x*_-coated TiO_2_ structure resulted in a well-connected network of crystalline IrO_2_, improving conductivity while maintaining high catalytic activity and low Ir packing density.

Compared to forming core–shell type structures, another route is to directly improve the electrical conductivity of the support particles, such that the electronic transport within a CL of supported catalyst no longer becomes limited by the IrO_*x*_, and in theory, loadings can be reduced beyond the IrO_*x*_ percolation threshold. Doping of various metal oxide supports has been investigated in several studies to improve support conductivity. Hartig-Weiss et al.^170^ synthesized an Ir-supported antimony-doped tin oxide (Ir/ATO) with just 11 wt % Ir. They demonstrated nearly 25 times better mass activity (@ 1.45 V_*iR*‑free_) compared to the commercial 75 wt % IrO_*x*_/TiO_2_ catalyst, which they attributed to good Ir dispersion, good conductivity of the antimony-doped tin oxide (ATO) support, and strong metal–support interaction (SMSI). Moreover, Böhm et al.^171^ combined the strategies of porosity tuning with support doping to synthesize IrO_2_ nanoparticles on a macroporous inverse opal ATO, resulting in a catalyst with just 25 wt % Ir and improved activity over commercial IrO_*x*_/TiO_2_ catalyst. Alternative support materials, such as substoichiometric titanium oxide (TiO_2‑x_)^172^ and titanium oxynitride (TiON_*x*_),^173,174^ are also being explored for improvements in support conductivity.

##### 2.2.3.4 PTL/CL Interface

The issue of poor electrical conductivity also has important implications at the PTL/CL interface. Since the PTL is inherently porous, not all IrO_*x*_ particles are in direct contact with it. Isolated particles may still be electrochemically active if they are connected via an in-plane, percolating conductive network. However, reduced CL thickness and poor connectivity at low loadings limit the formation of these pathways, leaving more catalyst electrically isolated and unutilized.^160^ Therefore, high interfacial contact between the CL and PTL is essential for ensuring electrical connectivity and catalyst accessibility. Yet, since the PTL must also enable gas and water transport, a balance must be struck between solid–solid contact and maintaining adequate pore space. This creates a complex optimization problem that depends on local morphology, pore alignment, and material properties at the PTL/CL interface.

While much of the literature has focused on PTL-side optimization, several strategies have emerged from the CL side to improve interfacial conductivity and overall performance. One approach is to incorporate conductive additives into IrO_*x*_-based CLs to enhance in-plane electron transport and CL thickness for improved contact between the CL and the PTL, thereby reducing the fraction of electrically isolated and unutilized catalyst particles. Recently, Ferner and Litster^57^ and Liao et al.^175^ integrated Pt black as a conductive additive to reach high performance at low Ir loadings of 0.10 mg_Ir_ cm^–2^. Alternatively, nanostructured supports and architectures such as nanofiber interlayers,^176^ nanosheets,^177^ nanostructured textiles,^178^ and hierarchical or graded porosity structures^179^ offer enhanced connectivity, interfacial contact, and durability. For example, Hegge et al.^176^ incorporated Ir nanofibers as an interlayer between the PTL and a layer of Ir nanoparticles. They demonstrated improved performance of this hybrid CL at low loadings (0.2 mg_Ir_ cm^–2^) compared to a high loading (1.2 mg_Ir_ cm^–2^) Ir nanoparticle CL, as well as improved durability compared to a low loading CL without the nanofiber interlayer. This study emphasized the benefits of the hybrid CL, which combined the advantages of the high activity and ECSA of the Ir nanoparticle layer with high electrical conductivity and PTL contact provided by the nanofiber layer. Overall, given that the PTL/CL interface has been identified as one of the mechanically and electrically weakest points of the anode structure,^55,68^ further investigation of this interface remains a promising direction. Improved microstructural design and a deeper understanding of how CL architecture influences interfacial properties could unlock further improvements in Ir utilization, performance, and durability.

#### 2.2.4 Degradation

In addition to producing high-performance catalysts and CLs for the anode, understanding and reducing degradation is crucial. Degradation studies aim to meet or exceed DOE targets for PEMWE operations of more than 80,000 h using less than 2.0 μV h^–1^ and reaching a performance better than 1.8 V at 3 A cm^–2^.^65,180^ This goal can only be achieved by closing the research gap, studying the mechanisms by which physical and operational factors impact degradation and failure.^65^ While stability is often discussed in conjunction with durability, an important distinction should be made. Although the stability of the catalyst in the CL impacts the durability of its performance, durability also refers to the degradation resulting from the long-term operation of a PEMWE. Degradation is a consequence of both insufficient stability and limited durability. As mentioned previously, Ir-based catalysts exhibit the best stability, while Ru-based catalysts offer better kinetic performance but rapidly degrade; thus, choosing the catalyst has a significant impact on degradation. Some important degradation mechanisms seen in CLs are catalyst dissolution, leaching, agglomeration, and poisoning.^180^ Dissolution occurs when the catalyst material breaks down, causing structural changes. Leaching refers to the dissolution of the active components in the catalyst in the surrounding liquid, which deactivates the catalyst. Agglomeration involves the catalyst particles clumping together, thereby reducing performance by decreasing the available active area. Poisoning of the catalyst occurs in the presence of contaminants, which also harms activity. These modes of degradation occur through mechanical or chemical mechanisms, which are explained below.

##### 2.2.4.1 Chemical Degradation

Chemical dissolution is one of the most critical degradation mechanisms in CLs, particularly in high-voltage operation.^180^ At elevated cell potentials, Ir and Pt catalysts undergo oxidation and dissolution, leading to structural changes that can block proton transport pathways and reduce overall conductivity.^180^ In the case of IrO_*x*_, the catalyst can experience valence state transitions depending on the applied voltage. These redox transitions can lead to lattice instability, loss of crystallinity, and the formation of amorphous phases, all of which contribute to irreversible performance losses.^64^ Additionally, catalyst particle agglomeration and morphological evolution further reduce the ECSA, limiting reaction kinetics and accelerating overall degradation.^24^ These effects are often exacerbated under dynamic or unsteady operating conditions, where repeated voltage cycling promotes both chemical and mechanical instability of the CL.

##### 2.2.4.2 Mechanical Degradation

Given the drive to reduce precious metal content in CLs, it is essential to understand how lower loadings influence degradation. Studies have shown that decreasing loadings often compromises durability due to poor CL uniformity and increased susceptibility to mechanical degradation. The morphology of the CL, including porosity, thickness, and particle dispersion, plays a crucial role in both long-term performance and stability, making it a key focus in current research. Mechanical stresses at interfaces, especially between the PTL and CL, can lead to the formation of microcracks during compression. These cracks disrupt electronic pathways, reduce conductivity, and create localized non-uniformities that alter temperature distribution.^180^ Additionally, poor contact and structural mismatches at the PTL/CL interface can limit catalyst utilization and accelerate degradation.^24^ High differential pressures across the MEA further exacerbate mechanical failure risks, leading to PTL penetration into the CL, formation of pinholes, and crack propagation.^78,181^ It is also important to recognize that degradation mechanisms differ between the anode and cathode, necessitating interface and electrode-specific durability strategies.

##### 2.2.4.3 AST Development

Catalyst degradation is reported to worsen during long-term operation of PEMWEs.^64^ ASTs are critical tools for investigating these degradation mechanisms in CLs. They allow researchers to simulate harsh, long-term operating conditions within a manageable time frame, providing valuable insights into performance loss and catalyst stability. Unlike fuel cells, PEMWEs degrade under different electrochemical conditions and tend to degrade more slowly. Thus, protocols designed for PEMFCs are not directly applicable, and significantly more aggressive testing (up to 100 times faster) is often required to generate meaningful degradation data for PEMWEs. Current PEMWE AST strategies typically involve cycling the cell between high and low potentials under specific gas flows. For example, cycling between high and low potentials at the anode can mimic the transient and dynamic conditions seen in an industrial-scale setting.^181,182^ Observing the catalyst’s stability in realistic operating conditions is essential as it highlights which mechanisms of degradation are more prevalent, and in a study by Weiß et al.,^183^ it was observed that during open circuit voltage (OCV) and operating potential cycling, IrO_*x*_ reduced the surface of the catalyst at the anode to a hydrous IrO_*x*_, which is more likely to corrode. Current holds and current cycling have also been investigated as stressors on the catalyst. Researchers have revealed that high current density holds resulted in significant performance losses due to catalyst dissolution,^64^ whereas compared to current cycling, potential cycling had a more severe effect on catalyst durability by greatly reducing the ECSA. Performance decay due to catalyst contamination from impurities in feedwater has also been investigated, revealing that it caused corrosion and subsequent thinning of the CL.^64^ The ongoing development of AST protocols aims to establish reliable and reproducible methods that accurately reflect real-world degradation and support the DOE’s durability targets.

#### 2.2.5 Ionomer

The role of the ionomer in the CL is multifaceted. It acts as a proton conductor, a binder for the catalyst, and it also preserves moisture.^28,107^ Due to its role as a proton conductor, decreasing the amount of ionomer is thought to cause poor kinetics from reduced proton transport pathways, while increasing the amount of ionomer can also block the transport of reactant water and produced oxygen.^21,107^ Thus, optimizing the amount of ionomer is a critical area of research for designing CLs. Some studies have found that an ideal ionomer content is about 2.4–5 wt %.^107,184^ Khandavalli et al.^184^ noticed that higher ionomer content affects the viscosity of the liquid ink that is used to fabricate the CL, causing the resulting microstructure to be heterogeneous with lower catalyst utilization and poor performance, concluding that rheological properties significantly impact the final CL microstructure. There is also research studying CL fabrication without the use of an ionomer. Lee et al.^130^ studied CLs without ionomer, and the results demonstrated better electrode kinetics and maximized catalyst utilization while maintaining good durability. The benefits of exploring ionomer-free CL fabrication are the reduced material cost of fabrication, better safety from eliminating flammable ionomer, and simplification of the precious metals in the CL.^130^

The translation of catalyst performance interrogated in an aqueous-electrolyte environment using RDE measurements to MEA performance is not trivial due to the critical role of the ionomer/catalyst interactions. Elucidating the role of ionomer in catalyst inks and CLs has been a focus in PEMFCs, motivating a wealth of studies on ionomer thin films and interfaces to understand their role in transport resistances in the electrodes.^17^ Ionomer exists as a nanometer-thick thin film in CLs, wherein their structural and transport characteristics deviate from their bulk membrane form, contributing to electrode performance limitations at the electrode.^185^ A recent work by the Berkeley group investigating the Nafion-based ionomer inks and thin films interfacing various metallic and functionalized Ir catalysts confirms the role of these ionomer-catalyst interactions in modulating the electrolyzer’s performance.^186^ Nafion’s swelling in thin film form (<50 nm) and binding strength with the catalyst exhibit strong dependence on Ir surface composition and properties (such as hydrophilicity), which are related to the overpotentials observed at the cell level.^186^ For example, Nafion binds most strongly to IrOOH, likely yielding reduced kinetic overpotentials, but swells more on metallic surfaces, likely improving transport and ohmic overpotentials. Thus, the role of ionomer-catalyst interactions in electrolyzer electrodes is governed by a set of complex and competing factors, which should be examined further, especially in relation to the ink optimization, ionomer development, and catalyst-layer design and performance at higher current densities. For example, the ionomer also alters the particle size distribution of the Ir-based catalyst inks, albeit with a varying impact depending on the catalyst type^186^ (see Figure 9). It has also been shown that the interplay between the ionomer chemistry and solvent composition impacts the ionomer structure–functionality,^187^ which paves the way to tune the catalyst-ionomers using ink preparation and fabrication methods as discussed next.

**Figure 9:**
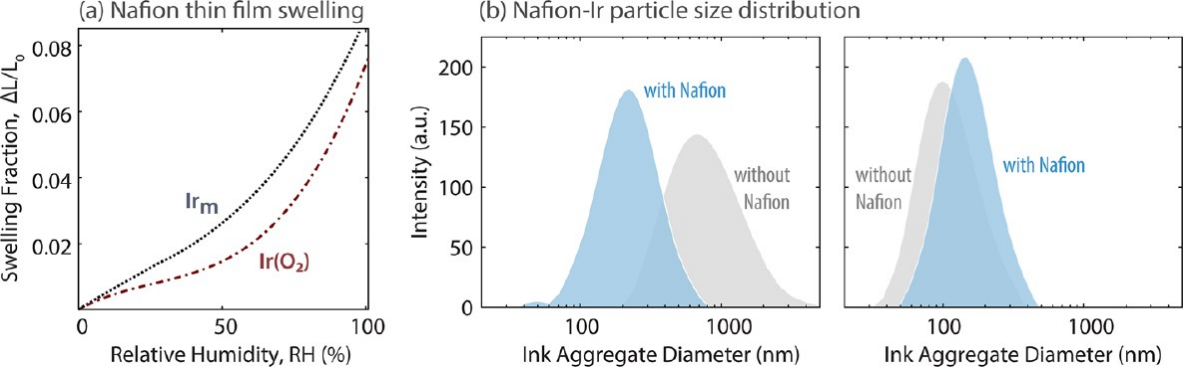
(a) Humidity-dependent swelling of Nafion thin films (40 ± 5 nm) on metallic Ir_m_ and IrO_2_, and (b) aggregate diameters of Ir_m_ (left) and IrO_2_ (right) catalyst inks with (blue) and without (gray) Nafion. Reproduced from ref 186. Copyright 2024 American Chemical Society.

#### 2.2.6 Preparation Methods

##### 2.2.6.1 Ink Fabrication

Although, as mentioned, there are several types of fabrication methods for different CL designs and structures, the following section will focus on the preparation of conventional CLs. This fabrication typically begins by combining an Ir-based catalyst, ionomer, and solvents into a slurry. This is followed by a dispersion procedure to create an ink mixture that can be coated onto a substrate to create the final CL. Figure 10 depicts an example of the steps of ink preparation, ink dispersion, CL coating, and CL configuration. Although there are several parameters in the ink fabrication procedure, there is limited research on the systematic impacts of changing each variable. However, the most influential factors for CL design may be the ionomer content and dispersion of the catalyst.^28,107,184^ Some effects of changing the ink composition on performance have been studied, giving insight into best practices for CL ink fabrication. Kang et al.^21^ found that the performance of PEMWEs has high flexibility and low sensitivity to various ink fabrication methods. For example, the ratio of the solvents incorporated, usually deionized water and propanol, is negligible, and changing the catalyst weight percentage also showed insignificant performance changes, but did have an impact on high-frequency resistance (HFR) and kinetics.^21^ Alia et al.^188^ determined that modifying ionomer content in the catalyst ink largely affected kinetics, where excess ionomer slowed down the kinetics, likely because of the ionomer blocking access to the catalyst particle surface and decreasing CL porosity, which impedes electron transport pathways. Similarly, the dispersion of the ink helps break apart large clusters of catalyst, ensuring higher surface area utilization. Liu et al.^189^ indicated that, different from Pt/C catalysts, the particle size distribution and rheological behavior of the Ir catalyst make it challenging to form a stably dispersed ink; thus, it is important for the catalyst to have fewer large aggregates that would cause heterogeneous CLs. To reduce the aggregates in the mixture, common dispersion techniques include bath or horn sonication, typically in an ice bath, and ball milling.^29,40,57,188,189^ Some studies combine several different types of dispersion techniques and lengthen dispersion duration to maximize catalyst dispersion and minimize particle size, but Liu et al.^189^ concluded that although initially there is a large reduction in particle size through more dispersion, this effect decreases over time and further dispersion past a certain point does not significantly impact the particle size distribution. Overall, this ink fabrication process is often time-consuming,^39^ and other methods to eliminate the need for a coating method are being explored, which will be discussed in the next subsection.

**Figure 10:**
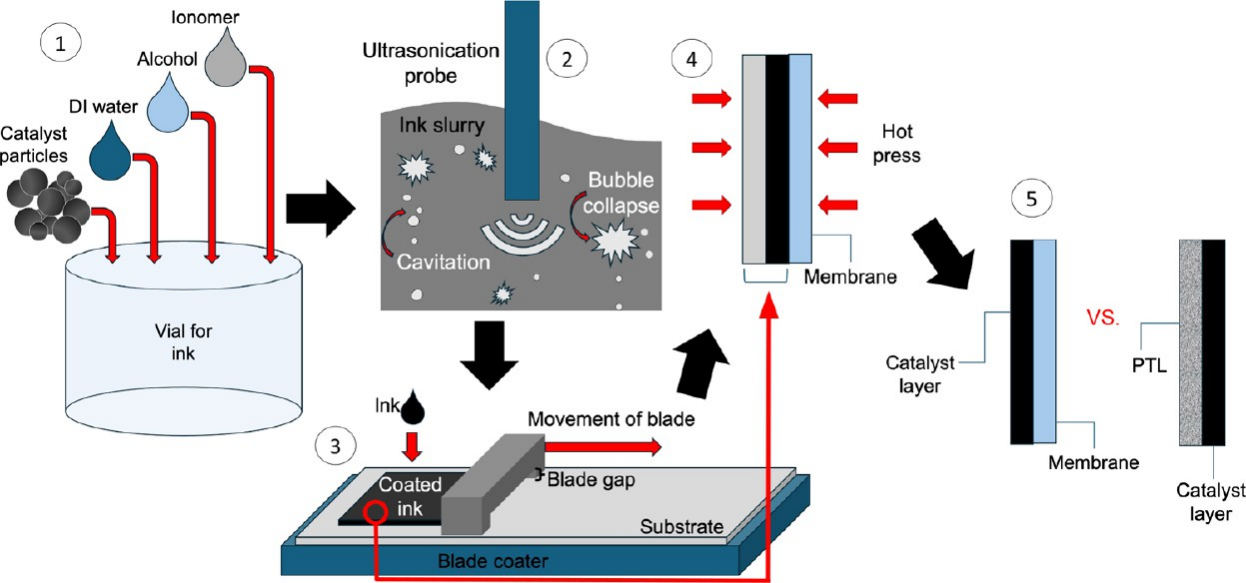
Conventional CL preparation: (i) ink slurry is made by combining catalyst particles, solvents, and ionomer; (ii) ink is dispersed via methods like ultrasonication; (iii) ink is coated onto a substrate using blade coater; (iv) CL is hot pressed onto membrane and peeled off the substrate; and (v) anode CL is on a membrane (CCM configuration) or, alternatively, on a PTL (PTE configuration).

##### 2.2.6.2 Coating Methods

After preparing the ink slurry, CCMs are fabricated by either transfer or direct coating onto the proton-conducting membrane. The transferring process via hot-press avoids swelling and deformation of the membrane. Direct coating methods include ultrasonic spray or aerosol, roll-to-roll processes such as slot die, and hand painting. Ultrasonic spray is suitable for small-scale and slot die gravure or knife can achieve continuous coatings, improving production efficiency and reducing costs, but needs to be optimized for slurry composition and processing conditions.^29^ Ultrasonic spraying the CL is widely used, but the decal transfer by hot press is also a common fabrication technique for CCMs because it has lower ohmic and transport resistance compared to spray.^22,40,41,44,188,190,191^ Other processes that do not involve a coating method are techniques like reactive spray deposition technology (RSDT), NSTFs, DMD, inkjet printing, and other chemical or physical vapor deposition methods.^22,107^ These methods eliminate the ink fabrication step and can enhance contact resistance between the membrane and the CL, thereby improving performance.

##### 2.2.6.3 CCM vs GDE/PTE

There is the option of having the CL coated on the membrane, creating a CCM, or alternatively to have the CL coated on the PTL, creating a PTE on the anode side or a GDE on the cathode side.^190^ The CCM method seems to be more commonly used as it allows for zero gap between the CL and the membrane resulting in lower ohmic losses, good mechanical stability, and low mass transport losses,^22,103,107^ but research shows that there are advantages and drawbacks of both electrode types and the PTE/GDE type MEAs can be competitive with the CCM type MEAs.^18^ For CCMs, decoupling the PEM/CL interface results in less membrane and CL deformation.^22^ In addition, the choice of membrane is wider for PTE/GDE.^35^ For PTE/GDEs, the pore size must be optimized because small pores cause excessive mass transfer resistance while large pores lead to catalyst infiltration.^29^ Some disadvantages of PTE/GDEs are poor cohesion between the catalyst and PEM, which could result in high protonic resistance, low catalyst utilization, and poor mechanical stability. Generally, CCM configurations have better contact between the membrane and the catalyst, which can reduce the interfacial impedance and improve the conductivity and durability of the proton, but may cause swelling of the membrane.^29^ Membrane swelling can be avoided by decal transfer processes, making it an ideal method for CCMs.^107^

### 2.3 Cathode CL

For PEMWE cathodes, Pt/C is the most commonly used catalyst material as Pt is known to have one of the highest activities for the HER. Due to the significantly lower potentials seen at the cathode, the Pt catalyst can be dispersed onto carbon support particles, and fabrication of these Pt/C cathode CLs follows similar techniques as those discussed for the PEMWE anode CLs.^29,189,192^ The ability to disperse Pt nanoparticles on a viable support, combined with the fast kinetics of the HER, enables a considerable reduction in Pt loading. One key study by Bernt et al.^127^ showed that while performance losses were seen when anode Ir loadings decreased below 0.5 mg_Ir_ cm^–2^, cathode Pt loadings could be reduced from 0.30 to 0.025 mg_Ir_ cm^–2^ without any significant performance loss. However, long-term durability at such low Pt loadings may be compromised.^193^ Yu et al.^194^ used advanced post-mortem imaging and analysis techniques to show that a significant amount of Pt dissolution from a 0.30 mg_Pt_ cm^–2^ cathode was redeposited in the membrane after a 4,500 h current hold. On the other hand, some studies have examined alternative support materials to carbon, such as TiO_2_, to avoid possible carbon oxidation and improve the stability of the Pt catalyst.^44,195^ Alternative nanoscale structures have also been investigated, such as the NSTF with Pt or Pt alloys.^196^ Overall, the primary efforts for the cathode CL have been to not only develop novel HER catalysts with reduced Pt loading for lower costs, but to concurrently improve our understanding of degradation mechanisms specific to low-Pt-loading cathodes.

## 3 Membrane

### 3.1 Introduction

PEMs used in electrolysis applications have historically been made from PFSA ionomers about 175 μm thick (i.e., Chemours Nafion 117). The first generation of rather thick membranes was made by melt extruding the polymer in the sulfonyl fluoride form followed by hydrolysis and acidification to form the sulfonic acid side chain. These materials were first commercialized for the chlor-alkali industry, thus making them readily available for the PEMWE research community and early demonstrations.^197–199^ The first ionomer of this kind was commercialized by DuPont (now Chemours) under the Nafion trade name. Later, the two-carbon side chain version was developed by Dow and commercialized by Solvay (now Syensqo), and the four-carbon version made by 3 M (now discontinued). All three structures are shown in Figure 11, designated as short, medium, and long side chains.

**Figure 11:**
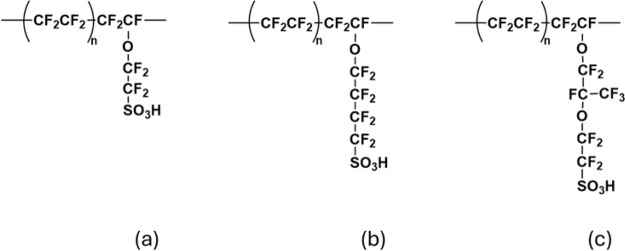
Structures of the three main PFSA ionomers. (a) Short side chain made by Syensqo, (b) medium side chain – now discontinued – from 3M, and (c) long side chain from Chemours, AGC, and others.

As the market matures, and both system cost and operating costs become increasingly important, the industry is moving to thinner and thinner membranes. Not only do thinner membranes have less of the expensive ionomer per square meter of active area, but they also have lower proton resistance, resulting in lower ohmic losses and an increase in cell efficiency.^164^ Extruded Nafion 115 (125 μm) is becoming more common in commercial systems, with indications that membranes thinner than 100 μm are being considered.^200,201^ This move to thinner membranes comes with a transition to solution casting from dispersions of the PFSA ionomer in mixtures of alcohol and water.^202,203^

Thinner membranes, however, come with trade-offs, especially increased concerns regarding mechanical and chemical durability. Drawing parallels with fuel cell reports, increased oxygen crossover to the cathode may result in increased peroxide formation.^204^ In addition, thinner membranes have higher hydrogen crossover to the oxygen side, presenting a possible safety issue. Fortunately, solution casting is well-suited for the introduction of mechanical reinforcements such as expanded polytetrafluoroethylene (ePTFE), chemical stabilizing additives, and gas recombination catalysts. From a practical point of view, the thicknesses of PFSA membranes are unlikely to go below about 50 μm as the loss of hydrogen due to crossover (even with gas recombination catalyst (GRC)) starts to offset the advantages of the reduced ohmic losses.^205^

### 3.2 Mechanical Durability

Mechanical durability of membranes is governed by the interplay between the membrane’s intrinsic stability and the external stressors acting on the membrane, which primarily arise from assembly (cell) and operational (environmental) loads. The construction of an individual cell starting from the MEA surrounded by the gaskets at the edges and sandwiched by the PTL (anode side) and GDL (cathode side) results in mechanical stresses in and through the plane directions of the membrane.^78^ These stresses are required to ensure adequate contact between the components (edge) gaskets and end plates to optimize/minimize contact resistance while preventing/minimizing leaks. In PEMWEs, the clamping stress can be anywhere from 0.5 to 1.55 MPa, while above 2.5 MPa, ohmic losses were reported to increase.^206,207^ In addition, electrolyzer membranes operate in a hydrated state at elevated temperatures, resulting in dimensional expansion compared to their initial state, which leads to swelling-driven compressive stresses.^77,78^ While this concept, in principle, is similar to widely studied phenomena in fuel cells wherein the membrane is subjected to rapidly alternating stress states following the load (duty) cycles,^72,77^ the operational characteristics of electrolyzers result in long-term constant loads maintaining a relatively steady level of hydration. For this reason, the mechanical property of interest in electrolyzer membranes becomes creep, which is the deformation of the ionomer under a constant stress, causing a change in thickness (which is a reduction under compression) of a hydrated membrane under prolonged times. Moreover, PEMWEs are commonly operated with a differential or back pressure of 1.5–3.0 MPa (15–30 bar) to deliver pressurized hydrogen, which results in additional compressive stresses acting on the membrane.^70,198,208,209^ A direct consequence of compression creep is the reduced membrane thickness, although in reality, all components deform in tandem under constraints and mechanical loads;^78^ however, the non-uniformity of the coarse PTL that provides space for the membrane to flow (creep), thereby triggering interfacial deformation. These distortions at the membrane-electrode-PTL interfaces could create additional complications and failure points, which have several implications. While a reduced membrane thickness would imply a lower ohmic resistance, it would also increase gas crossover and, more importantly, trigger interfacial failure mechanisms, especially in the presence of non-uniform contacts at the membrane-electrode-PTL interfaces, which are expected to deform under stress, thereby compromising the chemical-mechanical durability.^78^ For example, Hintzen et al.^210^ associated membrane creep in an operating water electrolyzer with changes in local current density. The authors suggested that the regions with higher local current density could experience higher hydrogen crossover. Moreover, Arthurs and Kusoglu^70^ showed, using a controlled mechanical testing methodology, that PFSA membranes under compression exhibit creep, in the form of reduced thickness, which is higher for hydrated membranes and under higher compression loads. The work of Arthurs and Kusoglu^70^ is summarized in Figure 12. As an example, for a fully hydrated Nafion 117 membrane held under 10–35 MPa pressure, the thickness reduces by 6–10 μm over 24 h, corresponding to 0.25–0.4 μm per hour.^70^ Even though in a device environment, the creep might be lower due to lower stresses and the constrained geometry of the membrane, these studies demonstrate the role of dimensional stability (swelling in water) and creep compliance (ability to withstand prolonged compression loads) in characterizing the mechanical stability. To address the stability concerns and reduce gas crossover, a thicker membrane has been favored in electrolysis. However, improving performance and reducing cost and ohmic resistances necessitate thinner but stable membranes, which can be achieved through mechanical reinforcement, enhancing the dimensional stability and chemical stabilization via additives to eliminate the crossover gases.

**Figure 12:**
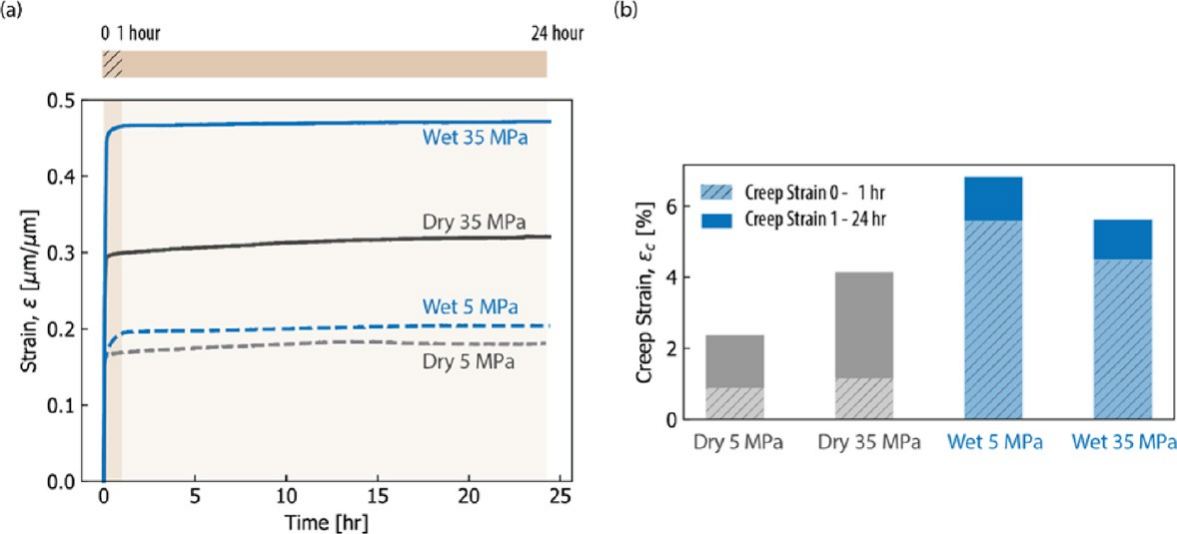
(a) Compression creep of pretreated Nafion 117 at 5 and 35 MPa and in dry and wet conditions. (b) Creep strain of these tests with the creep strain of the first hour in light blue and the creep strain after the first hour in blue. Reproduced from ref 70. Copyright 2021 American Chemical Society.

Compression behavior, therefore, is more critical for understanding membrane behavior and durability in electrolyzers, where the PEM experiences a high-pressure, hot liquid environment during longer operating times. Furthermore, creep failure or membrane thinning has been observed in Nafion-type membranes, especially at elevated temperatures, such as 80 °C.^198,208^ As much as 75% thickness reduction has been observed in electrolyzer cells during accelerated stress testing.^209^ Thus, for electrolyzers, it is even more imperative that mechanical properties be studied in a compressive mode, especially in water, as this is more relevant to device operation and will inform device lifetime.

### 3.3 Reinforcement Strategies

Reinforcements are incorporated into PEMs with the aim of improving handleability, strength, and mechanical durability. Primarily, the incorporation of porous ePTFE has been shown to be an effective strategy in fuel cell membranes, especially by increasing the dimensional stability in response to RH swings^72,77,211^ as demonstrated by improved lifetime under mechanical RH cyclical tests and other AST studies.^212,213^ The most common reinforcement is ePTFE and, more recently, for PEMWE membranes, monofilament woven webs incorporated as one or several layers in the membrane in a roll-to-roll coating process.^214–216^ Electrospun nanofibers are also potential candidates, but have not yet been incorporated into a commercial PEM.^217,218^

Reinforcements are well understood to increase mechanical strength, reduce gas crossover, and in-plane swelling at the expense of increased through-plane swelling (with a moderate effect on overall hydration). Despite the increase in thickness, reinforcements are still effective at reducing the in-plane stresses during operation.^72,77,219^ These effects generally increase as the amount of reinforcement increases, but at the penalty of increased membrane resistance (at the same thickness) and an associated decrease in performance. Thus, it is necessary to balance the mechanical durability imparted by the reinforcement and the performance from the ionomer. Work by Giancola et al.^217^ on electrospun polysulfone-reinforced PFSA membranes investigated the effect of equivalent weight (EW) on membrane properties. The polysulfone reinforcement was shown to reduce membrane swelling and improve tensile mechanical properties relative to unreinforced membranes. Interestingly, the differences between reinforced and unreinforced membranes became more pronounced as the ionomer EW decreased. Note that it is attractive to use ionomers with low EW for performance benefits due to higher conductivity. However, a decrease in EW is generally related to membranes with higher swelling and lower hydrated mechanical properties because water effectively acts as a plasticizer of the polymer chains but, more importantly, increases the fraction of hydrophilic domains in the membrane.^17^ The work of Giancola et al.^217^ suggests that reinforcements may be particularly effective for improving the mechanical durability of low-EW ionomers, resulting in membranes with both improved performance and mechanical durability.

Woven webs are of particular interest as novel water electrolyzer membrane reinforcements, since they can be manufactured from a range of engineering polymers and thus have the potential to be mechanically stronger than ePTFE or electrospun nanofibers. Typical attributes of woven webs are fiber diameters of ∼30 μm and mesh size of ∼200 μm.^216,220,221^ Notably, this relatively large mesh size may reduce the difficulties of incorporating woven webs into PEMs. However, an open woven reinforcement structure results in PEMs that may have more heterogeneous local mechanical properties, as the ionomer is unsupported in the relatively large spaces between fibers (∼100 μm). For example, a nanoindentation study conducted by Borowec et al.^222^ found that the local compressive reduced modulus and hardness of a woven web-reinforced membrane were higher in areas directly in the vicinity of a fiber of the web.

### 3.4 Chemical Durability

The chemical stability of PFSA membranes has been studied for several decades. Despite their exceptional thermal, hydrolytic, and oxidative stability, these membranes are still subject to degradation in some circumstances. The most direct evidence is fluoride ions detected in effluent water.^223,224^ Early on, researchers identified the carboxylic acid end groups as vulnerable to attack by peroxyl radicals (HO·), leading to the “unzipping” of the polymer backbone.^202,225,226^ These radicals originate from hydrogen peroxide formed when oxygen crosses over to the hydrogen electrode and reacts in the presence of the Pt catalyst.^204,227,228^ A significant advance in the stabilization of PFSA ionomers was the capping of the end groups by converting them to stable perfluoro methyl groups (−CF_3_).^17,229,230^ Despite this, degradation is still observed in accelerated fuel cell testing at OCV under dry conditions (90 °C, 30% RH).^223,224^ Subsequently, other reactions were proposed where peroxyl radicals (HO·) can attack the sulfonic acid group or the side chain ether.^231,232^ It must be noted that there is some controversy regarding the stability of the perfluoro ether linkage, with some publications claiming it is too stable to be degraded by peroxyl radicals^226,230^ while others suggest it is a weak link.^233,234^ A less referenced degradation mechanism is the reaction of hydrogen atoms (H·) with the tertiary fluorine in the backbone or side chain of the ionomer.^226,235,236^ Hydrogen atoms are thought to come from either peroxyl radicals abstracting a hydrogen from hydrogen gas^226^ or from “hydrogen spill over” at the catalyst surface of the hydrogen electrode.^237^ In any event, there is ample evidence for hydrogen atom chemistry during fuel cell accelerated lifetime testing.^235,236,238,239^ The locations where chemical attacks by radicals are reported and the simple degradation products are shown in Figure 13. It should be noted that fluorinated organic compounds have also been reported in the outlet water of fuel cells under highly ASTs.^236,240–242^ Since all of the reactions proposed involve a radical intermediate, the introduction of inorganic free radical scavengers such as cerium or manganese ions or oxides has proven to be a major advance in the lifetimes of fuel cell membranes.^231,243–245^ Extensive discussions on the chemical stability of PFSA ionomers can be found in one of the many excellent reviews on this topic.^17,246–248^

**Figure 13:**
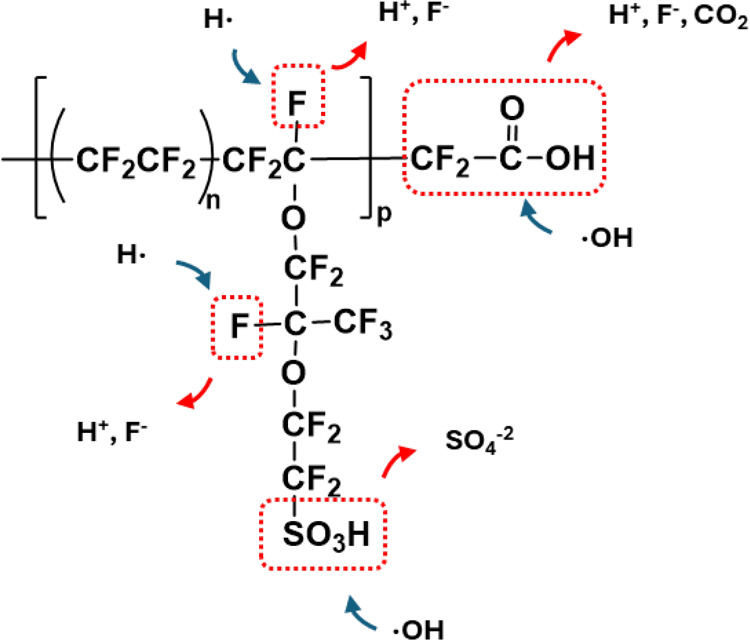
Primary reaction sites for radical degradation of a long side chain ionomer by either peroxyl radicals (HO·) or hydrogen atoms (H·).

While the chemical stability of PFSA membranes has been widely studied in fuel cells, less is known for PEMWEs. This may be due, in part, to the historical choice of thick membranes such as Nafion 117 or 115 (175 and 125 μm, respectively) or the presumption that degradation rates are lowest when the membrane is fully hydrated. In addition, fluoride measurements are more difficult in electrolyzer applications owing to dilution by large volumes of water and the use of polishing filters to remove trace levels of anions and cations. Still, researchers recognized nearly 35 years ago that fluoride release rates (FRR) are important in understanding electrolyzer membrane lifetime^197^ and that membrane thinning would occur under some operating conditions.^198^

There is little doubt that end group unzipping through reactions with hydroxyl radicals is the predominant mechanism in PFSA degradation, but it is not clear to what extent this occurs under electrolysis conditions, or if there are other significant mechanisms like those observed in fuel cells. In an early study, end-of-life (EOL) membranes were titrated and found to have the same ion-exchange capacity (IEC) despite experiencing significant thinning.^198^ This result implies no detectable degradation of the sulfate groups. Other evidence for end group unzipping under hydrated conditions was demonstrated by Coms et al.,^231^ where vapor phase hydrogen peroxide experiments showed that the fluoride release rate remains largely constant under high humidity conditions and dramatically increases under low humidity (30% RH). Importantly, when the samples were returned to high humidity, the fluoride release rate was higher than before the low RH excursion. This experiment suggests new chain ends are formed due to backbone cleavage under dry conditions and unzipping is the main reaction for hydrated membranes. In addition to the well-known peroxide chemistry, there is ample evidence of hydrogen atom (H·) attack on the tertiary fluorine in low RH accelerated fuel cell testing,^235,236,238^ but we are not aware of similar evidence in electrolysis conditions. In total, the literature to date seems to suggest that unzipping of the polymer via the carboxylic acid end groups is the likely degradation pathway for well-hydrated membranes, and as such, is potentially the most important for electrolyzer membranes. A greater understanding of the chemical durability of PEMWE membranes is complicated by the limited reported lifetimes and the wide range of FRR. A collection of literature values for FRR as a function of current density are summarized in Figure 14, where the range spans 3 orders of magnitude from around 10 ng cm^–2^ h^–1^ up to nearly 10 μg cm^–2^ h^–1^. Most FRR data is from the cathode water only, with some papers reporting fluoride measurements from the anode side. Typically, anode fluoride is negligible,^249^ but meaningful levels have been reported at the beginning of life (BOL) that decrease over time.^250^ The reported FRR numbers are qualitatively in agreement with EOL cross sections showing membrane thinning. In two cases where membrane thinning was reported, values of 2.6 μg cm^–2^ h^–1^ are estimated from the beginning and end of the test thickness measurements.^209,251^

**Figure 14:**
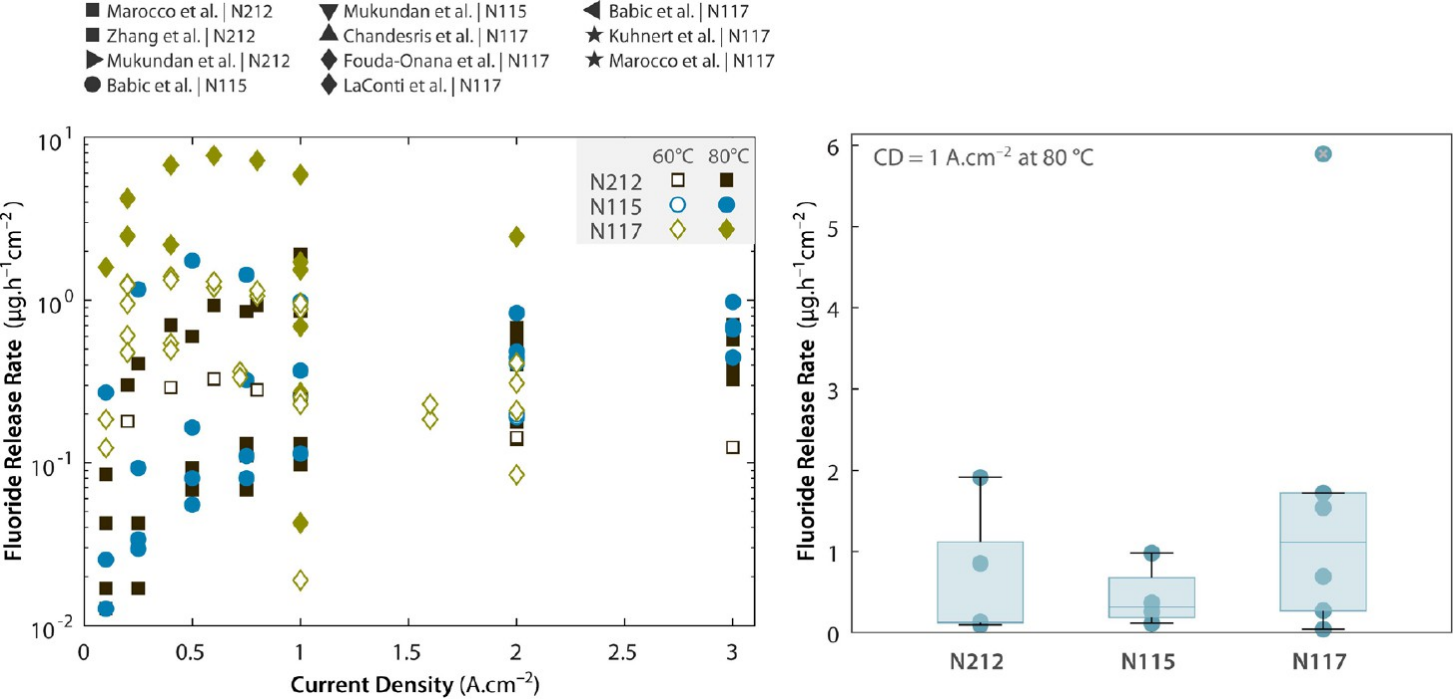
(Left) Literature reported FRR as a function of current density, and (right) averaged data at 80 °C and 1 A cm^–2^ for the three main membrane types reported.

While each study has somewhat different objectives, Table 3 shows that the material sets and operating conditions are largely similar, suggesting other factors are responsible for the large discrepancy in FRR. Variables such as current density, cell temperature, GRC, and differential pressure are often the main variables studied, but others may play an equally large role. It is well-known from the fuel cell literature that certain ions like iron­(II) or titanium­(IV) will catalyze the decomposition of molecular peroxide into reactive hydroxyl radicals (HO·).^252–254^ Because these ions are not always quantified when undertaking an electrolyzer durability test, there is the possibility that the variability in the literature is due, in part, to variable levels of Fenton active ions. Likewise, ionomer end group modification is usually not quantified for the membranes studied. One notable exception is the work of Frensch^252^ that considered both factors, demonstrating careful accounting of iron (Fe) and end group concentrations, showing these variables are indeed important.

**Table 3:** Reference, Catalyst Loadings, and Operating Conditions for FRR Reported in the Literature

author	membrane	anode (mg cm^–2^)	cathode (mg cm^–2^)	temperature (°C)	pressure (bar)
Marocco et al.^249^	N117 and N212	2.0 Ir	1.0 Pt	60 and 80	ambient
Zhang et al.^255^	N212	2.0 Ir	0.4 Pt–C	80	ambient
Fouda-Onana et al.^251^	N117	2.0 IrO_2_	2.0 Pt	60 and 80	1 bar
Chandesris et al.^256^	N117	1.94 IrO_2_	1.2 Pt	60 and 80	atmospheric
LaConti et al.^199^	N117	4.0 Pt–Ir	4.0 Pt	48, 60, 82, 149	6.7 bar
Mukundan et al.^213^	N212 and N115	0.1 IrO_2_	0.1 Pt–C	80	ambient and 5 bar
Babic et al.^257^	N115 and N117	Ir (no detail)	Pt (no detail)	60	assumed ambient
Kuhnert et al.^250^	N117	2.0 Ir	1.0 Pt	not specified	not specified

Despite the variability of reported results, membrane thickness is a key parameter expected to impact FRR. Higher gas crossover rates are associated with higher peroxide formation in fuel cells,^204^ and it is reasonable to expect the same effect in PEMWEs. Surprisingly, in publications where different thickness membranes were studied, the trend was the opposite.^249,258^ The reasons for this result are not clear but may lie in the previously discussed Fe or end group variables or related to cast (NR212) versus extruded (N117 or N115) processing. In both examples, the authors compare thick extruded membranes (N117 or N115, 175, and 125 μm, respectively) to a thinner cast membrane (NR212, 50 μm). Peroxide scavenging additives such as manganese and cerium ions and oxides have been shown to dramatically increase the lifetimes of fuel cell membranes.^224,243–245^ It stands to reason that these additives would also benefit PEMWE membranes. However, there have been no detailed comparisons between electrolysis membranes with and without these additives. Perhaps this is due to the historic use of extruded membranes, where incorporation of ions is difficult. Even if effective, this class of inorganic ion additive is known to be very mobile and may or may not remain in the regions where it is most needed.^259^

Regardless of the exact degradation mechanism, the trend toward thinner membranes will require greater chemical stability. Using the metric of 10% membrane loss proposed by LaConti et al.^199^ and typical lifetime targets of 20,000, 40,000, and 80,000 h, one can generate the allowable average FRR as a function of thickness. The average FRR at each of these lifetime targets is plotted as a function of membrane thickness in Figure 15, showing that the tolerable FRR decreases as membrane thickness decreases. In other words, as the industry moves to increasingly thinner membranes, the rate of peroxide generation due to increased oxygen crossover is likely to increase, and the tolerable FRR will need to simultaneously decrease.

**Figure 15:**
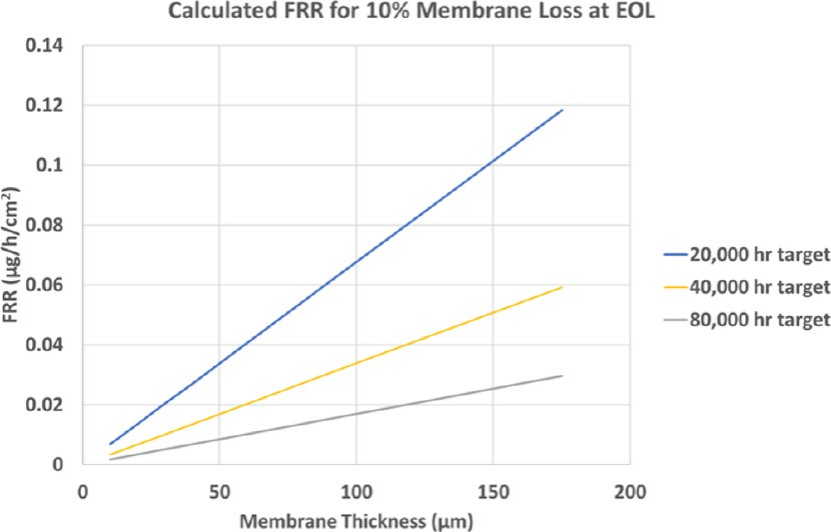
Estimated fluoride release rate (FRR) as a function of membrane thickness needed to maintain 10% loss of membrane for three typical electrolysis lifetime targets.

### 3.5 Hydrocarbon Membranes

Almost all PEM water electrolysis membranes used today are based on PFSA ionomers. These materials have a proven record in a variety of applications such as chlor-alkali electrolysis, fuel cells, flow batteries, and electrolyzers.^17^ However, fluorinated materials have come under increasing scrutiny due to the adverse effects of some per- or polyfluorinated alkyl substances (PFAS).^260^ Most notably, the 2023 proposal by the European Chemical Agency (ECHA) aims to ban the manufacture, placing on the market, or use of PFAS substances, mixtures, or articles.^261^ While exemptions and derogations are expected based on socio-economic considerations, it is incumbent on the PEM research community to seek alternatives. As shown in Figure 16, hydrocarbon (HC) ionomers are a PFAS-free alternative ionomer that typically consists of sulfonated aromatic polymers. However, to be commercially viable they must demonstrate properties equal to or exceeding those of PFSA ionomers like Nafion. Key metrics for membrane comparison are water uptake, mechanical durability, ionic conductivity, gas crossover, and resistance to chemical degradation.

**Figure 16:**
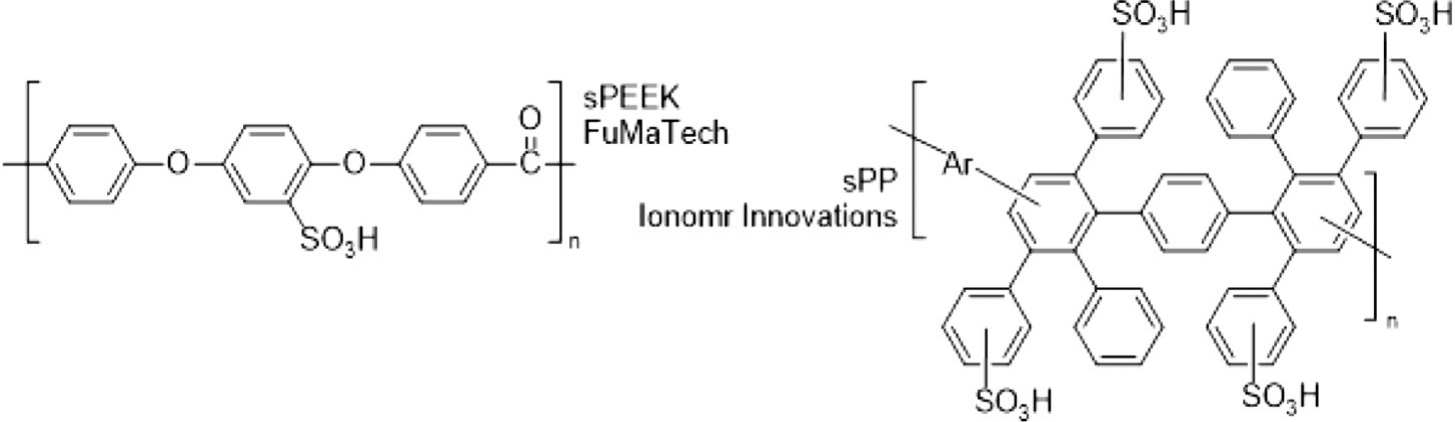
Examples of hydrocarbon ionomers from FuMaTech, sulfonated polyetheretherketone (sPEEK), and Ionomr Innovations, sulfonated polyphenylene (sPP).

#### 3.5.1 Swelling

Membrane swelling is the result of sulfonic acid groups absorbing water. These acid groups provide the protons necessary for ionic conduction, thus creating a trade-off between sulfonic acid group content and swelling. More acid groups naturally mean more ions for conduction and more swelling. This relationship is shown in Figure 17 for literature values of PFSA, hydrocarbon, and fluorine-containing hydrocarbon membranes, where increasing the moles of sulfonic acid groups per gram of polymer (known as IEC) correlates with increasing water uptake (WU, mass of water sorbed divided by the mass of dry membrane). High degrees of swelling are undesirable, as this usually results in increased gas crossover and diminished mechanical properties.

**Figure 17:**
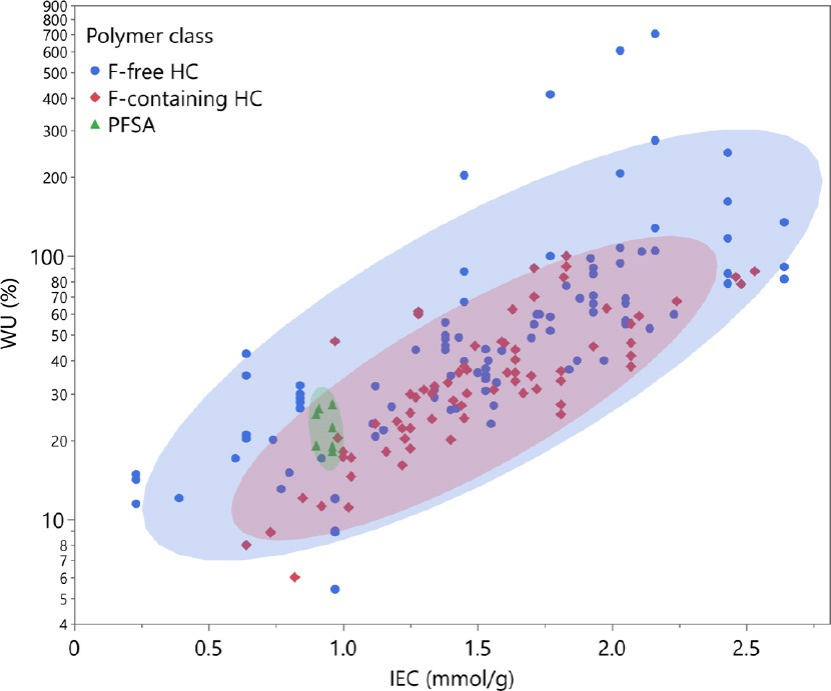
Water uptake (WU) against IEC. All data collected after submersion in water. Water temperatures between 20 and 95 °C.^262–272^

Comparing the ionomer types over this wide range of IECs, no statistical difference is observed in water uptake (WU). Instead, the dominant contributor to a given membrane’s water uptake is its acid content, not the membrane’s backbone or side chain structure. This relationship is discussed in a recent publication by Bangay et al.^273^ and suggests that well-designed HC ionomers have the potential to compete with PFSAs for water uptake and conductivity.

#### 3.5.2 Mechanical Durability

Comparing HC and PFSA ionomers in non-submerged (i.e., dry) conditions, HC typically exhibits higher Young’s modulus (HC: > 500 MPa, PFSA: < 300 MPa) and lower break strain than PFSA ionomers (HC: <100%, PFSA: >100%).^262,267,268,274–277^ These results show that hydrocarbons are generally more resistant to instantaneous deformation, whereas PFSAs, owing to their semicrystalline matrix, could exhibit a more ductile deformation before break. However, higher modulus also means a higher stress generated in a constrained membrane during swelling, and mechanical durability is too complex to be quantified by a single metric or property like stiffness.^77^ For example, recent publications suggest that the critical mechanical property in water electrolysis is creep.^70^ Studies comparing HC and PFSA by Bae et al.^278^ and Saito et al.^279^ demonstrate that creep measured in ambient conditions is similar between both ionomer types. Both publications note that HC membranes have higher IECs, and thus contain more water, suggesting that for the same internal water content between the compared HC and PFSA, HC should creep less. Mirfarsi et al.^280^ compared the changes in the mechanical properties of a PFSA and an HC ionomer as temperature and hydration level changed. The results demonstrated that HC mechanical properties are more sensitive to water content than typical PFSA ionomers. This illustrates the importance of water management via IEC change for HC ionomer. Moreover, the device-level mechanical durability is complicated by the membrane-electrode-PTL interactions and interfacial deformation, which are expected to exacerbate further with hydration and pressure effects.

#### 3.5.3 Ionic Conductivity

High ionic conductivity of a membrane is critical for low cell resistance, resulting in better energy conversion efficiency. Figure 18 illustrates the relationship between IEC and conductivity for a large group of literature reports. Focusing on the IEC region of around 0.9 mmol g^–1^ (as this is the only IEC with both PFSA and HC data), HC polymers exhibit lower conductivity than PFSAs. This suggests that HC ionomer conductivity is inferior to PFSA at a given IEC. However, this conclusion is misleading, as conductivity is a volumetric property, not gravimetric, as it is typically used for IEC (mmol g^–1^). Sulfonic acid group concentration in mols per cm^3^ is a more useful comparison and can be calculated by multiplying IEC by membrane density (dry). Since the average HC density is approximately 30% lower than that of PFSAs (1.4 and 2 g cm^–3^, respectively), a PFSA ionomer with an IEC of ∼0.9 mmol g^–1^ should be compared with HC ionomers with an IEC of ∼1.4 mmol g^–1^.^17,271,273,281^ As the conductivity of PFSA at 1 mmol g^–1^ is similar to the conductivity of HC at 1.4 mmol g^–1^ in Figure 18, it suggests that HC ionomers demonstrate largely the same ionic behavior in fully hydrated, i.e., PEMWE conditions.

**Figure 18:**
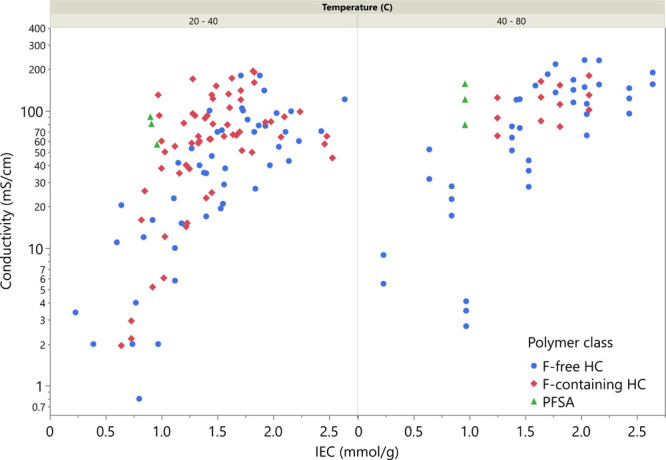
Ionic conductivity against IEC. All data from membranes submerged in water. (Left) temperature between 20 and 40 °C, and (right) temperature between 40 and 80 °C.^262–272^

#### 3.5.4 Gas Permeability Coefficient

In PEMWEs, gas permeation should be minimized for safety, efficiency, and durability.^205^ For both hydrogen and oxygen, HC ionomers exhibit lower permeability coefficients than PFSA ionomers, as shown in Figure 19. While these results are exclusively from fuel cell testing, electrolysis testing of HC ionomers by Klose et al.^32^ demonstrates that the lower gas crossover observed at high RH translates to the fully hydrated (submerged) environment.

**Figure 19:**
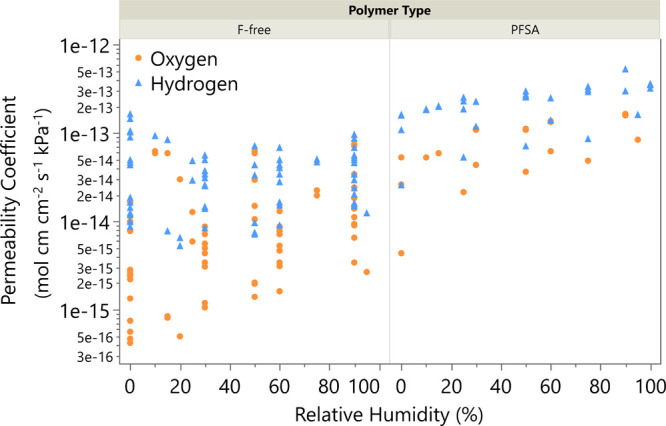
Hydrogen and oxygen permeability coefficients against RH. Measured at 80 °C.^274–276,278,279,282–285^

#### 3.5.5 Chemical Durability

HC ionomers have consistently demonstrated a lower resistance to oxidative attack during ex-situ experiments, such as Fenton tests.^286^ However, this does not always translate to in-cell experiments owing to the fact that reactive radicals must first form to cause this degradation.^248^ In this regard, HCs are advantageous, since lower gas crossover implies less radical formation. A publication by Han et al.^287^ demonstrates that HC chemical degradation is most severe in wetter conditions when gas crossover is highest, in contrast to PFSA ionomers, where dry conditions accelerate degradation. This finding implies that chemical degradation of HC ionomers may be accelerated in PEMWE conditions.

When Klose et al.^32^ fabricated a full HC MEA for PEMWE conditions and ran a 1 A cm^–2^ hold test, both HC membranes failed within 100 h, while the PFSA comparator showed no signs of degradation. However, the publication notes that failure was due to electrical shorting, which was attributed to both the “lack of mechanical robustness” when immersed in water and radical degradation. Choi et al.^30^ also explored HC membranes for PEMWEs, using PFSA electrodes with interlocking tie layers to ensure a good membrane-electrode interface. Critically, membranes were tested both with and without cerium oxide in the interlocking layer. When held at 1 A cm^–2^ for 100 h, the voltage increase rate was 65 and 612 μV h^–1^ for the membrane with and without cerium oxide, respectively. This result suggests that additives will be necessary for increasing the lifetime of HC PEMWE membranes, either by quenching radical reactions or by facilitating the repair of HC radical adducts, as suggested by de Wild et al.^288^

## 4 Porous Transport Layer

### 4.1 Introduction

The anode PTL is responsible for liquid water transport to the anode CL, oxygen and heat removal, electron transport, and mechanical support to the membrane. It must be able to withstand the highly acidic environment and high cell voltage operating conditions, which limit the material choice to Ti. It must be sufficiently permeable and porous to facilitate liquid–gas transport but also have sufficient solid particle content to maintain good electrical and thermal conductivity.^289^ State-of-the-art PTLs are sintered Ti-powders or Ti felts and are typically sputtered with a Pt layer to prevent oxide formation and improve contact resistance.^290^ The cathode PTL, or the gas diffusion layer (GDL), does not have such material restrictions and shares the same carbon paper structure as GDLs used in PEMFC electrodes.^126^ Because the anode is responsible for the bulk of the overpotential losses in a PEMWE cell, there is little research effort into GDL materials optimization. As such, this section will review current anode PTL research efforts, particularly focusing on morphological considerations, interfacial engineering, novel fabrication methods, and Ti alternatives.

### 4.2 Morphological Impacts on Performance

PTL morphology can play a significant role in cell performance. Morphological parameters such as thickness, pore size, tortuosity, and pore size distribution (PSD) affect transport through PTL and across the PTL/CL interface. The two most studied types of PTLs, Ti fiber and sintered Ti powder PTLs, are presented here. Sintered PTLs typically have lower porosities, smaller mean pore diameters, and lower thicknesses than Ti fiber PTLs. Fiber PTLs are comprised of varying diameter Ti fibers bonded together to form a porous network. Ito et al.^291^ investigated the impact of porosity and pore diameter with various fiber PTLs and observed increased performance with decreased pore size down to 10 μm. Parra-Restrepo et al.^292^ performed a study in a segmented cell comparing sintered PTLs with porosities ranging between 26 and 37% and mean pore diameters between 3 and 60 μm. The authors observed increased performance as mean pore diameter decreased. This was attributed to lower contact resistance at the PTL/CL interface. A mean pore diameter of 3 μm resulted in mass transport limitations and hysteresis effects in the polarization curve. Yuan et al.^47^ reviewed a variety of PTL studies to identify structure-performance trends. The authors found a weak correlation between lower cell voltages and smaller pore diameters for both sintered powder PTLs and fiber PTLs. Increasing the porosity was also found to have a weak relationship with decreased cell voltage. Weber et al.^293^ investigated the impact of PTL thickness on performance. Several fiber PTLs with thicknesses varying from 2 to 0.16 mm were compared. Their results suggested an optimal thickness of 0.5 mm as mass transport losses were observed when PTLs were thinner than 0.25 mm. Several studies have determined that the largest impact of morphological properties is on the PTL/CL interface rather than bulk transport. Schuler et al.^55,294^ performed a comprehensive study comparing six different fiber PTLs. They quantified the contact area between PTL and CL and observed increased catalyst utilization and decreased contact resistance with increasing contact area. Peng et al.^53^ observed similar trends in their study on fiber PTL performance at low catalyst loadings. These results have motivated most research efforts to focus on improving the PTL/CL interface.

### 4.3 Interfacial Engineering

The PTL/CL interface is crucial for maintaining good electrical connectivity, high catalyst utilization, and enabling low catalyst loadings.^40,55,295,296^ Recent research efforts to improve the interface are discussed below.

#### 4.3.1 Microporous Layer

One way of improving the interface is the introduction of an MPL in between the anode CL and the PTL.^289^ Kulkarni et al.^297^ used operando X-ray computed tomography (XCT) to investigate the impact of MPLs on the interface and oxygen transport. The authors found that introducing an MPL can improve the TPCA by over 20% and improve contact point density, which lowers the electrical contact resistance. Figure 20 below visualizes the catalyst distribution and TPCA for a PTL and two MPLs. The addition of MPLs significantly increased catalyst utilization.

**Figure 20:**
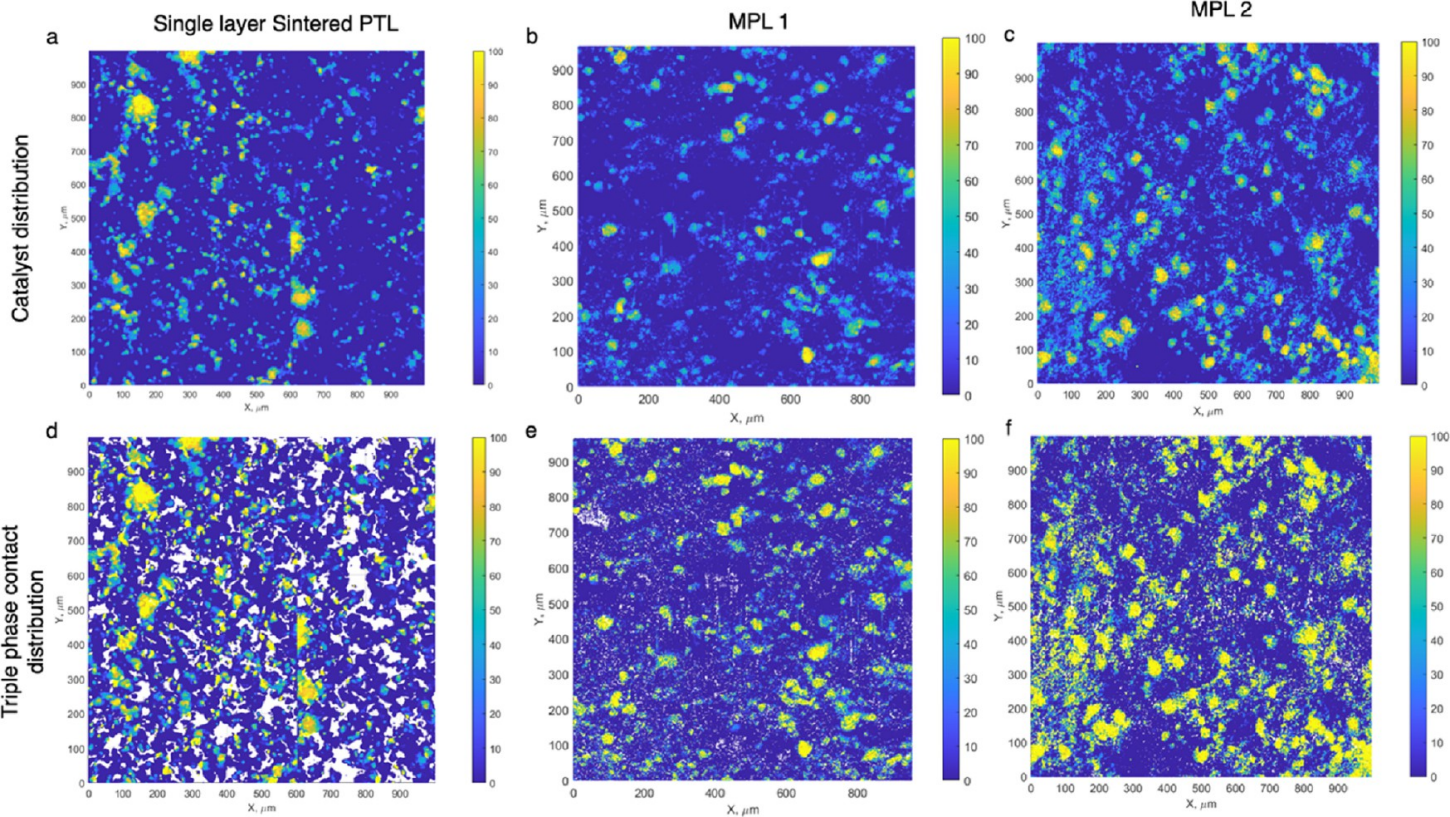
Contour plots visualizing the (a–c) catalyst distribution and (d–f) TPCA for a sintered PTL and two MPLs in a 1 mm^2^ region. The color bars represent the percentage of catalyst for catalyst distribution and the percentage of catalyst in contact with the region for TPCA. Adapted from ref 297. Copyright 2023 American Chemical Society.

They also suggested that MPLs should be designed with finer particle sizes and lower thicknesses to maximize contact point density and ensure sufficient oxygen removal. Recent literature has also shown improved performance with MPL at low Ir loadings (<0.4 mg_Ir_ cm^–2^) due to improved in-plane electrical conductivity and smoother surfaces enabling lower contact resistance.^41,42,298,299^ Notably, MPLs have also been suggested to enable the use of thinner membranes because they lower hydrogen permeation.^42^ Hasa et al.^40^ investigated the mechanisms of performance improvement and suggested that, in addition to the reduced contact resistance, MPLs also facilitate better oxygen removal and ensure homogeneous current distributions. Lee et al.^299^ also found that MPL performance improvements were agnostic to PTL type, which can potentially enable cheaper PTL alternatives. Laser-perforated Ti sheets were also used as MPLs by Garsuch et al.^300^ and demonstrated performance improvements because of decreased contact resistance between the Ti foil and CL, resulting in lower HFR. Another way to improve the interface is via surface modification. Lee et al.^301^ performed laser ablation on the surface of commercial sintered PTLs and reported a maximum reduction in cell potential of 230 mV compared to the unmodified PTL at ultralow loading of 0.055 mg_Ir_ cm^–2^. The MPLs in the previous studies were produced in a variety of ways, such as tape casting or calendaring Ti powders that were deposited onto a base PTL. It does not seem to be a consensus on a suitable fabrication method that is both affordable and easily scalable, as studies have focused primarily on MPL impacts and mechanistic understanding. Further research is needed to find optimal MPL microstructures and demonstrate MPL fabrication at scale and low cost.

#### 4.3.2 Surface Coatings and Treatments

Currently, Pt is sputtered onto PTLs to prevent passivation and lower interfacial contact resistance (ICR).^302^ Use of PGMs is expensive, and so there is a significant research effort into alternative coatings that either lower the precious metal content needed or eliminate precious metal usage altogether. An alternative coating should have high corrosion resistance to maintain durability comparable to Pt coatings and have low ICR. Alternatives to Pt that have been investigated include compounds based on niobium (Nb), tantalum (Ta), and Ti hydride (TiH_*x*_) formation via surface treatment.^97,302–304^ Stein et al.^305^ recently published a long-term durability test that compared precious metal coatings and Nb, TiH_*x*_, and TiN_*x*_ to an uncoated reference. Pt and Ir coatings were found to be highly stable after 2,000 h, with Pt still running up to 5,000 h and a degradation rate 93% lower than the uncoated PTL. The authors reported TiH_*x*_ to be the best non-precious metal coating due to its 49% lower degradation rate, as shown below in Figure 21, and decreased ohmic losses compared to the uncoated reference PTL.

**Figure 21:**
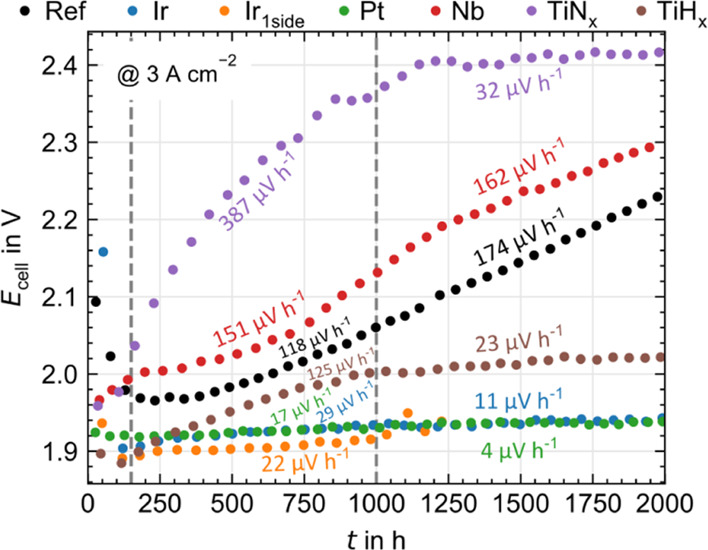
Plot showing cell potential at a current density of 3 A cm^–2^ vs time for different types of coatings. Adapted from ref 305. Copyright 2025 The Authors under Creative Commons Attribution 4.0 License (https://creativecommons.org/licenses/by/4.0/).

A potential way to reduce Pt loading is to use an interlayer where an alternative lower-cost material is first deposited onto the PTL and a Pt layer is subsequently deposited. Moradizadeh et al.^97^ investigated the effect of Ta interlayer thickness with a 50 nm Pt layer. A 320 nm Ta layer was found to be the optimal thickness for reducing contact resistance and improving corrosion resistance.

### 4.4 Fabrication Methods

Currently, commercial PTLs are produced by sintering Ti powder or by stacking Ti fibers and sintering.^306^ Fabrication methods that have been previously demonstrated in the literature include photolithography, tape casting, and vacuum plasma spraying.^306–309^ Photolithography offers the advantage of producing thin layers (25 μm) with straight-through pores of tunable diameters and has demonstrated good electrochemical performance due to better contact and lower electrical resistance.^307^ Further work has demonstrated the photolithographic process to be suitable for manufacturing MPLs and high-performance PTEs.^310,311^ Tape casting can be used to produce very large sheets and is a highly scalable manufacturing process. Ti powders are mixed with a solvent to form a slurry and then cast and subsequently sintered. Hackemüller et al.^306^ fabricated PTLs using tape casting. They compared two different kinds of Ti powders, gas-atomized (GA) and hydrogenation-dehydrogenation (HDH), at various sintering temperatures (800 °C – 1200 °*C*). The powder type and sintering temperature had significant impacts on the electrochemical performance and morphologies of the PTLs. They were also able to scale the process up, producing a porous Ti sheet of 470 × 470 mm^2^. Lee et al.^308^ later used poly­(methyl methacrylate) (PMMA) as a pore-former in the Ti slurry and fabricated PTLs using tape casting. They were able to tune the pore structure by varying the Ti:PMMA ratios in the slurry. In a later work, the authors used tape casting to produce MPLs.^299^

Recent alternative fabrication methods that have been studied are shockwave-induced spraying (SWIS), cold gas dynamic spraying (CDGS), high velocity flame spraying, and spark plasma sintering (SPS).^312–314^ SWIS and CDGS are proposed because they do not require a heat treatment process. Fakourihassanabadi et al.^312^ utilized SWIS and CDGS to produce highly porous (>60*%*) PTLs. The high porosity was achieved by using Al and Cu as pore-formers that were later leached out of the substrate. Spark plasma sintering offers the advantage of high thermal efficiency and lower temperatures, up to 500 °C lower than the conventional sinter process.^315^ Phuong et al.^313^ fabricated PTLs at sinter temperatures between 500 °C – 650 °C and compared the resulting microstructures as well as their electrochemical performance. They found that increasing the sinter temperature improved corrosion resistance, mechanical strength, electrical conductivity, and electrochemical performance. Bobzin et al.^314^ used high velocity flame spraying to produce PTLs with porosity gradients, with one side designed to function as an MPL. The resulting PTLs had average porosities of 32% and thicknesses of 500 μm, twice that of commercial PTLs. Despite the thickness difference, the thermally sprayed PTLs had similar electrochemical performance.

### 4.5 Alternatives to Titanium

There is also some research effort to find alternatives to Ti because of its cost. The most common alternative is stainless steel (SS). Using SS requires coatings to prevent corrosion and lower ICR. Daudt et al.^316^ fabricated porous sintered SS316L PTLs with a thin layer of Niobium (Nb) to limit corrosion. The total thickness of the PTL ranged from 333 to 369 μm, and the porosity ranged from 16% up to 35%. Electrochemical testing showed comparable performance to a tape-casted Ti-based PTL up to a current density of 1 A cm^–2^. Beyond this current density, the polarization curve formed an S-shape typical of cation contamination. In a later study, Stiber et al.^98^ coated SS with a Nb/Ti mixture. ICR measurements showed similar resistances compared to a standard Pt-coated sintered Ti PTL. The electrochemical performance was also similar to a cell with a standard PTL with a cell voltage of 1.9 V at 2 A cm^–2^. The authors also performed durability tests up to 1,450 h using a constant current density hold of 2 A cm^–2^ AST protocol and did not find any signs of cation contamination in the cell.

## 5 Bipolar Plate

### 5.1 Introduction

BPPs are a critical component of PEMWEs, performing multiple functions that directly influence the efficiency, durability, and cost of the system. Their design and material selection substantially affect the overall system’s performance and economic viability.^317,318^ BPPs electrically interconnect individual cells within a stack, enable electron conduction, direct the flow of reactant and product gases (hydrogen and oxygen), and provide mechanical support. Moreover, they assist in thermal management by incorporating cooling flow patterns, which help regulate the temperature of the MEA. Figure 22 illustrates the configuration of a stack, including the positioning of BPPs, MEAs, and integrated cooling channels. Given that BPPs can contribute around 25% of the total stack cost and must operate under harsh conditions - such as high current densities, acidic environments, high pressures, and elevated temperatures - materials engineering of these components is essential.^23^ Optimizing both design and material selection is key to improving performance, ensuring long-term durability, and reducing capital costs for scalable hydrogen production.^319–321^

**Figure 22:**
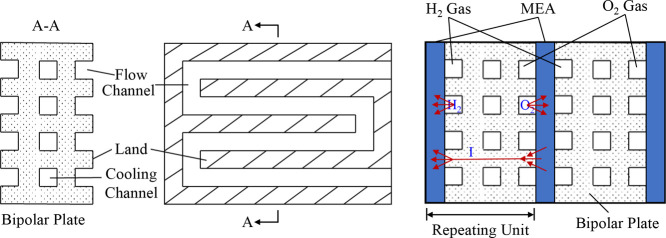
Schematic illustration of a PEMWE stack: (left) cross-sectional view of a BPP with an integrated cooling flow channel between the anode and cathode flow fields; (middle) face view of the flow-field design; and (right) repeating unit of the stack structure comprising BPPs and MEAs. Adapted from ref 319 with permissions from Elsevier B.V.

To ensure an efficient current distribution across the stack, BPPs must exhibit high electrical conductivity between adjacent MEAs, minimizing contact resistance and overall power loss.^322,323^ The flow fields etched onto the surfaces of the plates guide the supply of water and the removal of hydrogen and oxygen gases, maintaining optimal distribution across the MEA surface and supporting continuous electrochemical reactions. These flow fields must also ensure gas separation to prevent cross-contamination and safety risks, while simultaneously providing a secure seal under pressurized conditions. BPPs also offer essential mechanical reinforcement to the thin and brittle components within the stack, such as carbon-based electrodes, by maintaining their structural integrity under compression. Additionally, the heat generated during electrolysis must be efficiently dissipated to avoid thermal-related performance loss and material deterioration. Some advanced BPPs incorporate dedicated cooling channels that enhance thermal regulation across the stack, reducing temperature gradients and extending component lifespan.^317^

The flow-field design strongly affects mass transport and system efficiency. Optimized geometries aim to reduce pressure drop while maximizing gas–liquid contact and providing uniform flow distribution. Achieving these goals requires careful optimization of the geometry of the flow path, including the shape, width, depth, and orientation of the channel. Similarly, material selection for BPPs is a key factor that determines not only corrosion resistance and conductivity but also fabrication complexity and overall cost. The interplay between geometry and material properties makes the BPP design a multidimensional challenge requiring simultaneous consideration of electrochemical performance, durability, manufacturability, and cost-effectiveness.^322,324^ These performance requirements make the choice of material for BPPs a decisive factor in PEMWE stack efficiency. The following section evaluates key material classes used in current designs.

### 5.2 Materials for Bipolar Plates

The selection of materials for BPPs in PEMWEs plays a pivotal role in ensuring system efficiency, long-term durability, and economic feasibility. BPPs must endure highly corrosive acidic environments, elevated temperatures, and intense current densities, while simultaneously delivering low electrical resistivity, mechanical robustness, and cost-effective manufacturability.^317,318^ Additionally, these components significantly contribute to stack weight and volume, demanding materials with favorable strength-to-weight ratios. Common materials—such as Ti, SS, and carbon-based composites—must often be engineered or coated to improve their surface stability, lower ICR, and minimize performance degradation over time.^321,322^ The choice of material is thus dictated not only by its intrinsic properties but also by how well it responds to electrochemical aging, surface passivation, and manufacturing constraints specific to large-scale PEMWE deployment.^318,321^ In the following subsections, the most commonly used materials for PEMWE BPPs—namely Ti, SS, carbon-based composites, and nickel (Ni) alloys—are discussed in detail, with a focus on their electrochemical performance, degradation behavior, surface engineering approaches, and practical applicability in long-term operation.

#### 5.2.1 Titanium

Ti is widely regarded as one of the most suitable materials for BPPs in PEMWEs due to its exceptional corrosion resistance in acidic environments and long-term electrochemical stability. Its low electrical resistivity (∼4.2 × 10^–7^ Ω m), high thermal conductivity (∼21.9 W m^–1^ K^–1^), and minimal hydrogen permeability make it ideal for use under extreme anodic potentials (up to 2 V vs SHE) encountered during water electrolysis.^322^ These properties ensure efficient current conduction and heat dissipation while preventing hydrogen leakage through the plate structure. However, one of the primary challenges with Ti is its tendency to form a passivating oxide layer (TiO_2_) under anodic conditions. Although this layer enhances corrosion resistance, it also leads to a significant increase in ICR—often exceeding 100 mΩ cm^2^ without surface treatment.^322^ This elevated ICR reduces the overall efficiency of the stack by increasing ohmic losses at the BPP/PTL interface.

To address the formation of oxide layers on Ti BPPs, various surface engineering strategies have been developed. Titanium nitride (TiN) coatings enhance corrosion resistance and reduce ICR, making them promising for PEMWE applications.^325^ Similarly, Pt coatings suppress passive TiO_2_ layer formation, maintaining stable interfacial properties and improving long-term performance.^322^ Alternative coatings like zirconium nitride (ZrN), applied via reactive sputtering, have also demonstrated improved durability and electrochemical performance in acidic environments.^326^ Additionally, atomic layer deposition (ALD) offers a method to apply ultra-thin, uniform coatings that enhance surface conductivity without compromising Ti’s inherent corrosion resistance, offering a viable low-cost alternative to precious metal coatings. In summary, while Ti remains a preferred material for BPPs due to its stability, ongoing advancements in coating technologies remain essential to overcoming challenges related to contact resistance and economic scalability in large-scale hydrogen production.

#### 5.2.2 Stainless Steel

SS, particularly 316L, is considered a promising material for BPPs in PEMWEs due to its high mechanical strength and better ductility compared to graphite. However, its tendency to corrode in the acidic and oxidative conditions of PEMWEs leads to increased ICR and reduced durability over time. To overcome these limitations, protective coatings have been applied. Ti and Pt layers are among the most studied, offering enhanced corrosion resistance and stable electrical performance. For instance, Ti–Pt coated 316L plates have shown improved electrochemical stability and reduced ICR under PEMWE operating conditions.^327^ Niobium coatings also demonstrate long-term viability; in one study, Nb-coated SS operated for over 14,000 h at 65 °C with minimal performance degradation.^328^

In addition, conductive polymers such as polyaniline have been investigated for their ability to form corrosion-resistant barriers on SS surfaces. A study using a PANI/Zn-porphyrin composite coating showed improved corrosion resistance while maintaining suitable conductivity.^329^ Although these coatings improve the performance of SS in PEMWEs, achieving consistent long-term stability and cost-effective manufacturing remains a key challenge for widespread adoption.

#### 5.2.3 Carbon-Based Materials

Graphite and carbon-based materials are valued in BPP applications for their excellent electrical conductivity and corrosion resistance in acidic environments.^317,321^ However, their brittleness limits mechanical durability under compressive forces, making them prone to cracking and less suitable for long-term operation. At the anode, significant carbon corrosion reduces the thickness of the BPP, which increases the electrical contact resistance between the MEA and the current collector, ultimately resulting in degraded performance. To improve mechanical integrity while preserving electrochemical properties, researchers have developed carbon-polymer composites. Thermoplastic-bonded carbon plates, for example, have demonstrated stable performance for over 500 h under operational conditions.^321^ Carbon fiber-reinforced composites further enhance conductivity and strength while reducing weight. These materials can be processed through scalable techniques, offering a practical alternative to graphite-based plates.^330^

Recent work has also explored the addition of conductive nanomaterials such as carbon nanotubes (CNTs) and graphene. These fillers improve both electrical and mechanical properties by creating conductive pathways and reinforcing the matrix. For example, composite plates incorporating functionalized carbon nanofibers have shown significantly improved conductivity and flexural strength.^331^ While composite carbon materials show strong potential for next-generation PEMWE systems, challenges remain in fabrication uniformity, mechanical reliability, and large-scale cost optimization.

#### 5.2.4 Nickel and Nickel Alloys

Ni-based alloys, such as Inconel and Monel, are widely recognized for their remarkable chemical inertness and structural resilience under harsh conditions. These properties have made them attractive for various industrial applications, including energy systems operating under elevated temperatures and acidic conditions. In the context of PEMWEs, their relatively high electrical conductivity compared to SS and Ti has positioned them as potential candidates for BPPs.^332^

Research has shown that Monel 400 offers exceptional corrosion resistance, with a corrosion current density of 5.9 × 10^–7^ A cm^–2^ and a low ICR of 7.2 mΩ cm^2^ at 140 N cm^–2^, successfully meeting the 2025 DOE targets.^332^ Similarly, Inconel 625 has emerged as one of the most corrosion-tolerant alloys tested under high-temperature steam electrolysis, highlighting its potential applicability in PEMWE anodic operation.^333^ Despite these findings, it is crucial to conduct further research to fully assess the long-term performance of Ni-based alloys in PEMWE environments. Understanding their behavior under prolonged exposure to operational conditions will be essential in determining their viability as materials for BPPs in PEMWE systems. Based on the detailed evaluation of individual materials, Table 4 provides a comparative summary of the commonly used BPP materials in PEMWEs. This includes their electrical conductivity, corrosion resistance, ICR, mechanical strength, and practical engineering notes. The table highlights key trade-offs to consider in optimizing PEMWE stack performance.

**Table 4:** Key Properties of BPP Materials in PEMWEs

material	conductivity (S m^–1^)	corrosion resistance	ICR (with/without coating)	mechanical strength	notes
titanium	∼2.4 × 10^6^	excellent	high/low with TiN or Pt	moderate	requires coatings (e.g., TiN, Pt) to reduce ICR
316L SS	∼1.4 × 10^6^	moderate-low	moderate/low with Nb or Pt	high	cost-effective alternative with proper coatings
graphite	∼1.0 × 10^5^	excellent	low	low (brittle)	brittle; machining complexity
carbon composites	∼10^4^ to 10^5^	good	moderate	improved over graphite	lightweight; scalable; enhanced via nanofillers
Ni-based alloys	∼1.6 × 10^6^	good-excellent	low	very high	promising; requires more long-term validation

### 5.3 Design Considerations for Bipolar Plates

The design of BPPs for PEMWEs is a multifaceted challenge involving the careful integration of flow-field architecture, material selection, mechanical robustness, and thermal management. Among these, optimizing the flow-field geometry is particularly critical, as it governs the transport of water, hydrogen, oxygen, and coolant across the cell. Flow fields are typically etched, machined, or molded into the surface of the plates and must achieve a balance between minimizing pressure drop, maximizing reactive surface area, and ensuring uniform distribution of water and gas streams across the MEA. Poor flow distribution can lead to local flooding, gas accumulation, or dry-out, all of which degrade performance and reduce durability. As a result, flow-field design is often an iterative process involving computational fluid dynamics (CFD), experimental validation, and durability assessment. Classical patterns, such as serpentine, parallel and interdigitated geometries, have been widely adopted in both PEMFCs and PEMWEs, with recent adaptations aimed at addressing the unique challenges of electrolysis conditions.^319^ Figure 23 illustrates several representative flow-field configurations used in PEMWE stacks. The most widely studied and implemented flow-field patterns in PEMWEs include serpentine, parallel, and interdigitated designs, each with distinct transport and pressure characteristics, as discussed in the following subsections.

**Figure 23:**
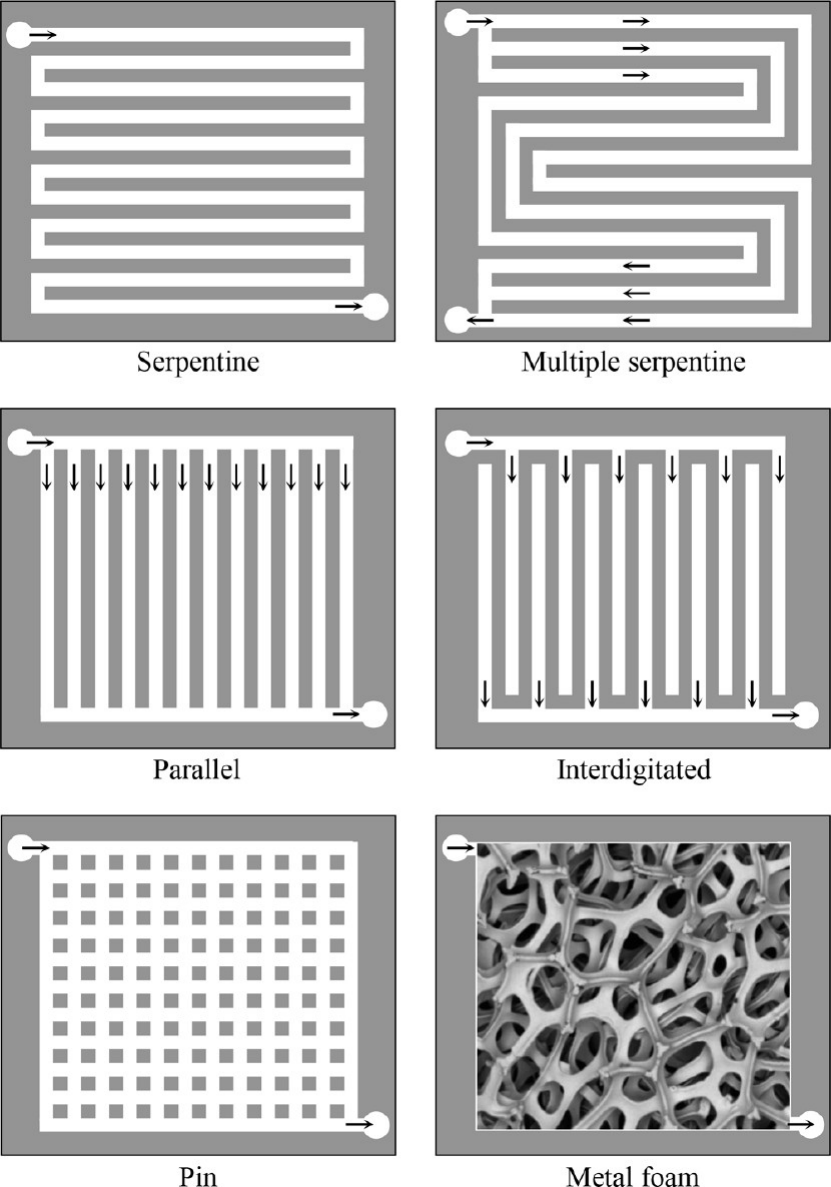
Representative flow-field configurations commonly used in PEMWEs, including serpentine, parallel, and interdigitated patterns. Reproduced from ref 319 with permissions from Elsevier B.V.

#### 5.3.1 Serpentine Flow Field

The serpentine flow field is a prevalent design in PEMWEs, characterized by a continuous, winding channel that spans the electrode surface. This configuration ensures a uniform distribution of reactants and efficient removal of gas bubbles, which is crucial for maintaining consistent electrochemical performance. The design’s inherent high-pressure drop enhances convective flow, thereby improving mass transport and reducing the likelihood of gas accumulation.^334^ However, the significant pressure losses associated with serpentine flow fields necessitate higher pumping power, impacting the overall energy efficiency of the system. Additionally, the complex flow path may lead to non-uniform compression across the MEA, potentially affecting the mechanical integrity and longevity of the stack.^335^ Recent advancements have focused on optimizing serpentine flow-field designs to mitigate these challenges. For instance, modifications such as the incorporation of circular arc columns within the serpentine channels have been proposed to enhance oxygen transport performance, thereby improving the overall efficiency of the PEMWE system.^336^ Furthermore, the integration of baffles within serpentine channels has been investigated as a means to enhance water removal and gas management. The strategic placement of baffles can disrupt flow patterns to prevent water accumulation and facilitate the removal of gas bubbles, thereby improving overall cell performance.^337^ In summary, while the serpentine flow-field design offers significant advantages in terms of reactant distribution and gas removal in PEMWEs, ongoing research and optimization are essential to address its inherent challenges. Tailoring channel configurations and integrating design modifications such as baffles and circular arc columns can lead to enhanced performance and durability of PEMWEs.^336^

#### 5.3.2 Parallel Flow Field

The parallel flow field consists of multiple straight channels aligned in parallel across the BPP surface. This configuration is widely recognized for its low-pressure drop, making it an energy-efficient option due to reduced pumping requirements.^334^ The minimal flow resistance in parallel designs allows for a more uniform distribution of pressure, which is beneficial in maintaining the mechanical integrity of the MEA over prolonged operation. However, the parallel configuration presents challenges related to flow maldistribution, particularly in the presence of two-phase flow conditions. The accumulation of gas bubbles can block specific channels, leading to uneven reactant supply and localized performance degradation.^338^ This non-uniformity can result in hotspots and dry-out zones, negatively impacting the durability and overall efficiency of the PEMWE system.

To address these limitations, researchers have explored various strategies such as modifying channel cross sections, incorporating flow restrictors, or integrating secondary structures to manage gas–liquid separation more effectively.^339^ Additionally, the combination of parallel and other flow-field designs (e.g., interdigitated or serpentine-parallel hybrids) has been proposed to balance the trade-offs between pressure drop and reactant distribution.^335^ In summary, while parallel flow fields offer advantages in terms of low energy consumption and mechanical stability, their susceptibility to flow maldistribution and gas clogging under dynamic operating conditions necessitates further optimization. Continued innovation in channel geometry and hybrid configurations is key to enhancing the viability of parallel designs in high-performance PEMWEs.

#### 5.3.3 Interdigitated Flow Field

The interdigitated flow field is a non-conventional but increasingly studied configuration for PEMWE due to its potential for enhanced mass transport. Unlike parallel or serpentine flow fields, interdigitated designs do not allow a direct flow path from inlet to outlet; instead, channels are blocked at one end, forcing reactants through the porous PTL. This mechanism intensifies convective mass transport by promoting through-plane flow, thereby reducing concentration gradients and improving the overall electrochemical reaction rate within the CL.^320,335^ As a result, interdigitated flow fields can significantly enhance reactant utilization and water delivery, particularly under high current density operation.

Several numerical and experimental studies have demonstrated that interdigitated configurations lead to more uniform current density distributions and improved gas removal efficiency, particularly for the evolved oxygen on the anode side.^339^ However, these advantages are accompanied by a notable increase in pressure drop due to the forced convection mechanism. This higher resistance requires greater pumping power, which can impact system efficiency if not properly optimized. In recent developments, researchers have proposed hybrid interdigitated-jet designs, integrating jet holes into interdigitated structures to further improve liquid water distribution and oxygen removal while minimizing parasitic losses.^340^

Although interdigitated flow fields are not yet standard in commercial PEMWEs, their performance benefits, especially in terms of mass transport enhancement and water management, make them a promising candidate for next-generation electrolyzer stacks. Ongoing optimization of channel geometries, blockage configurations, and integration with advanced materials (e.g., metal foams or engineered PTLs) continues to expand the feasibility of this design in high-efficiency, durable PEMWE systems.^320,340^ However, the implementation of interdigitated designs in commercial stacks remains limited due to increased fabrication complexity and higher associated costs compared to traditional flow fields.

#### 5.3.4 Pin Flow Field

The pin flow field, also referred to as pin-type or pin-array flow field, consists of an array of cylindrical or square pins arranged between the inlet and outlet manifolds. This design promotes three-dimensional and multidirectional flow patterns, enabling enhanced mixing and reactant distribution across the electrode surface. In contrast to conventional serpentine or parallel flow fields, the pin structure allows fluid to maneuver through a maze-like environment, which improves mass transport and facilitates more effective removal of oxygen bubbles formed on the anode side during electrolysis.^320,335^ The increased turbulence induced by flow obstruction around the pins significantly reduces concentration polarization, leading to more uniform current density and better water management within the cell. This makes pin-type flow fields particularly attractive for high current density applications or dynamic operating conditions where efficient gas removal and stable performance are critical.

Experimental studies and CFD simulations have confirmed that pin flow fields can outperform traditional designs in terms of pressure uniformity and bubble detachment, especially at higher current densities.^335,341^ Additionally, the pin geometry can be tailored (in terms of pin height, diameter, and spacing) to fine-tune the pressure drop and optimize convective transport without excessive energy penalties. However, the primary challenge associated with pin flow fields lies in the increased fabrication complexity and higher material usage, which may impact manufacturability and cost-effectiveness.

Despite these challenges, the unique benefits of pin-type configurations in promoting reactant access and efficient gas evacuation have led to growing interest in their application for advanced PEMWE systems. When combined with flow-field modifications or integrated into composite BPP structures, pin arrays offer a promising route toward improving electrolyzer performance and durability, especially under dynamic and high-demand operational regimes.^320,341^

#### 5.3.5 Metal Foams and Meshes

In PEMWEs, the design of the flow field plays a pivotal role in determining the efficiency of reactant distribution and the removal of generated gases. Traditional flow-field designs, such as serpentine and parallel configurations, have been widely employed. However, recent research has explored the use of metal foams and meshes as alternative flow distributors to enhance performance. Toghyani et al.^342^ conducted a comprehensive study comparing the performance of PEMWEs utilizing metal foam flow distributors against those with conventional serpentine and parallel flow fields. Their findings indicated that the metal foam flow field facilitated a more uniform distribution of current density and reduced pressure drops across the cell. This improvement is attributed to the high porosity and interconnected structure of metal foams, which promote effective mass transport and gas bubble removal from the electrode surface. Complementary studies have further substantiated the benefits of metal foam flow fields. For instance, Hassan et al.^334^ demonstrated that metal foam structures contribute to lower temperature gradients and homogeneous current density distribution, leading to enhanced hydrogen production rates. Similarly, research by Wu et al.^343^ highlighted the superior water management capabilities of metal foam flow fields, emphasizing their potential to improve overall cell performance and durability. Despite these advantages, the widespread adoption of metal foam-based flow fields in commercial PEMWEs is still constrained by manufacturing challenges, material costs and durability concerns under long-term operation.

While the integration of metal foams and meshes into PEMWEs presents challenges, such as material compatibility and manufacturing complexities, ongoing advancements in materials science and engineering are addressing these issues. The unique properties of metal foams and meshes, including their ability to enhance mass transport and reduce pressure drop, make them promising alternatives to traditional flow-field designs. Continued research and development in this area are essential to fully harness their benefits, paving the way for more efficient and durable electrolyzer systems. Table 5 summarizes the key design characteristics of the major flow-field types used in PEMWEs, highlighting trade-offs in pressure drop, reactant distribution, manufacturability, and water/gas removal efficiency.

**Table 5:** Qualitative Comparison of PEMWE Flow-Field Configurations Based on Key Design and Performance Parameters

flow field	pressure drop	reactant distribution	manufacturability	two-phase removal efficiency
serpentine	high	good	moderate	good
parallel	low	poor (under two-phase flow)	high	poor
interdigitated	moderate-high	excellent	low	excellent
pin-type	moderate	good	moderate	very good
metal foam/mesh	low	very good	moderate-low	excellent

### 5.4 Challenges in Bipolar Plate Development

Despite their essential role in PEMWEs, the development of BPPs continues to face several interrelated technical and economic challenges. A primary issue is the trade-off between cost and performance. BPPs account for a substantial fraction of the total stack cost - up to 25% or more in some cases - particularly when advanced materials such as Ti alloys or coated SS are employed to meet performance and corrosion resistance requirements.^317–319,322,324^ Although these materials improve durability and electrical conductivity, they significantly increase manufacturing costs. Therefore, identifying alternative materials or coatings that maintain electrochemical and mechanical performance while reducing cost is a key research priority.^321,323,329^

Flow field optimization presents another complex challenge. While CFD modeling has enabled progress in designing architectures that improve reactant distribution and minimize pressure losses,^320,335,340,344^ real-world implementation remains difficult. Flow fields must strike a delicate balance between low pressure drop, uniform water/gas management, and manufacturability - a task further complicated by evolving requirements at high current densities and dynamic operation.^334,337^ Moreover, complex designs such as interdigitated or pin-type geometries, while beneficial for mass transport, often impose higher fabrication complexity or material usage.^336,341^

Manufacturing remains a critical bottleneck, especially for composite and metallic plates that require precise machining, coating, or forming techniques. Achieving the required tolerances for thin, lightweight, and corrosion-resistant plates - while ensuring scalability - remains cost-intensive and process-sensitive.^317,319,324^ Long-term durability under electrolysis conditions is also a significant concern. BPPs are continuously exposed to harsh acidic environments, elevated temperatures, and gas-evolution-induced mechanical stress.^318,333^ These factors can lead to material degradation, delamination, or corrosion-induced performance loss over time. Even minor surface degradation can increase interfacial resistance and compromise stack reliability.^322,323,328^ To mitigate this issue, recent research has focused on corrosion-resistant coatings, such as TiN, Nb, or carbon-based layers, and surface treatments that maintain low ICR over extended operational lifetimes.^325,326,328^ Despite these challenges, ongoing innovations in materials science, surface engineering, and flow-field design are steadily improving the viability of advanced BPPs.^321,330,331^ As the global demand for green hydrogen accelerates, the role of BPPs will remain central to scaling PEMWE technology for cost-effective, efficient, and durable hydrogen production systems. Addressing these current limitations will be critical not only to optimizing individual components but also to enhancing the overall performance and reliability of PEMWE stacks.^317,320,342^

## 6 Gasket

### 6.1 Introduction

Gaskets in PEMWEs serve the dual purpose of sealing against hydrogen, oxygen, and water leakage and maintaining mechanical contact uniformity across the MEA. Proper gasket design has a direct impact on performance, electrical resistance, safety, and durability.^345^ Three main principal sealing structures can be identified (Figure 24):^346^

- Direct-seal frame. The gasket is compressed directly over the membrane inactive regions and, sometimes, between the MEA and BPPs to form primary and secondary seals, respectively. This approach is simple and reproducible but relies heavily on gasket softness and membrane compatibility.
- Rigid-wrapped frame. This method consists of adding a rigid PTFE/plastic support frame around the PEM edges. The rigid frame prevents membrane damage under compression, with rubber gaskets applied to enhance sealing.
- Seal-wrapped PEM/MEA configuration. The gasket is molded or injected around the PEM and bonded to form a unified cell component. This allows for robust high-pressure performance, even though at the expense of complicating disassembly.

**Figure 24:**
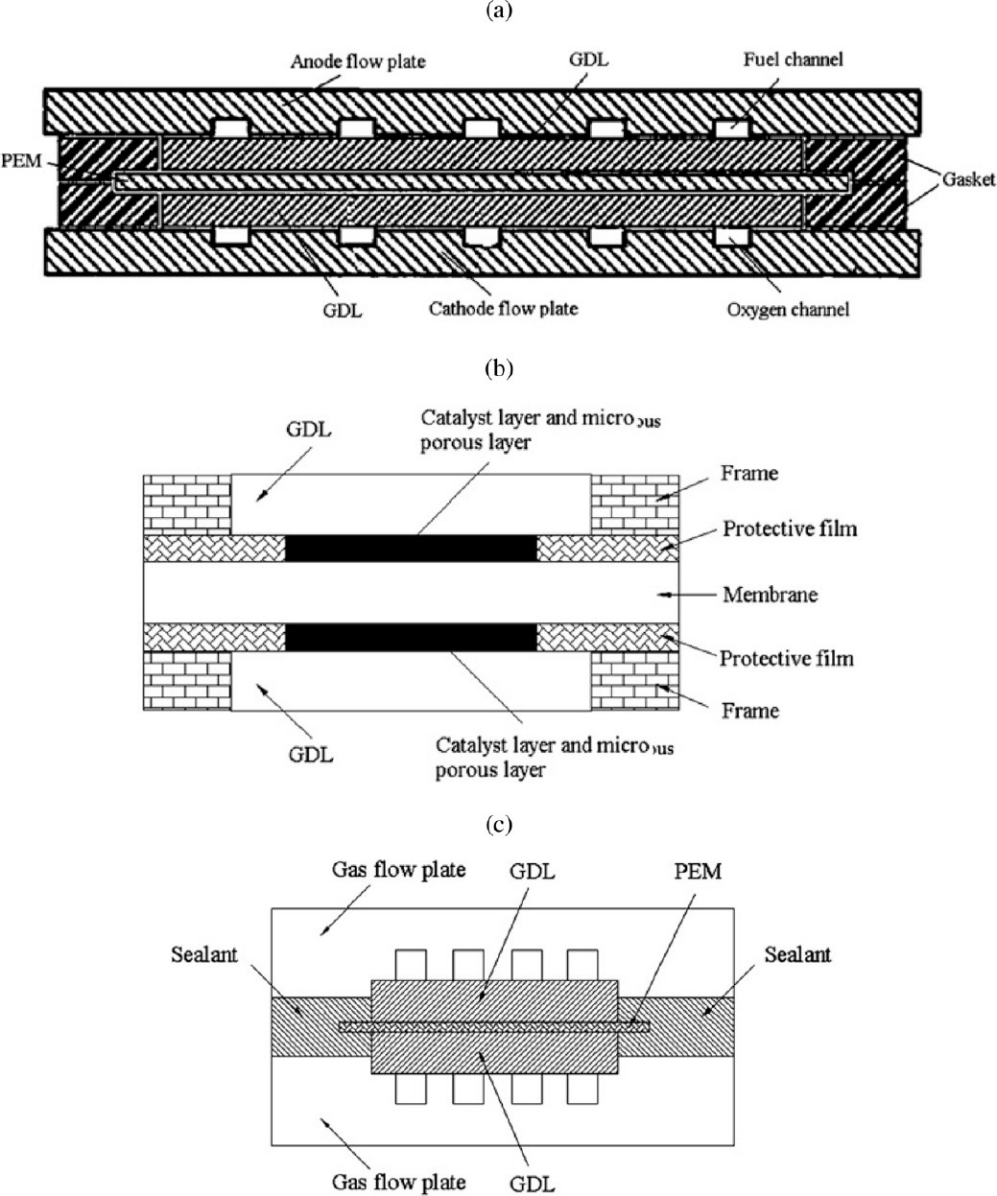
Schematic of different sealing structures used in PEMWEs: (a) PEM direct sealing structure, (b) rigid protective frame sealing, and (c) PEM-wrapped frame sealing structure. Adapted from ref 346 with permissions from Elsevier B.V.

Gasket compression behavior is typically modeled using finite element studies, incorporating hyperelastic constitutive models, such as the Mooney–Rivlin, Yeoh, or Ogden models. Additionally, they incorporate frictional contact and groove geometries to predict proper compression ratios and contact stress profiles.^347,348^

### 6.2 Material Selection

Typical sealing materials used in PEMWEs are PTFE and fluoroelastomers (FKM) due to their excellent chemical, thermal, and pressure resistance.^349^ ePTFE and structured PTFE are usually preferred due to their minimal creep, excellent chemical resistance, compressibility, and electrical insulation. ePTFE exhibits superior compliance and maintains seal integrity under dynamic conditions, while structured PTFE is particularly effective in thin-gasket, low-bolt-load stacks.^350^ FKM and ethylene propylene diene monomer (EPDM)/nitrile rubber may be used in lower-stress regions or combined with PTFE layers. Composite gaskets, which combine nitrile elasticity with PTFE surface layers, have shown a reduction of around 87% in gas crossover under dynamic conditions. Other advanced materials, such as graphene-enhanced FKM, are promising. Graphene nanoplatelets enhance tensile strength, gas-barrier properties, and mechanical durability, thereby improving long-term sealing.^351^ Metal gaskets (SS, Ni alloys) are sometimes employed when mechanical strength is important.

### 6.3 Performance and Durability Considerations

Gaskets influence PEMWE performance indirectly but significantly through mechanical boundary conditions. Non-uniform or excessive compression of the MEA and PTL, often caused by improper gasket design or assembly torque, results in increased contact resistance, localized CL damage, and electrochemical non-uniformity.^352,353^ Furthermore, gaskets serve to maintain consistent interface pressure, which supports optimal water and gas flow through the flow channels and across the active area. Compression mismanagement can distort flow fields, reduce membrane utilization, and lower stack efficiency.^354^

Gasket failure often manifests as mechanical creep, stress relaxation, or fatigue cracking under thermal and pressure cycling. These processes degrade the uniformity of compression over time, leading to a loss of seal integrity, progressive leakage of hydrogen and oxygen, and increased stack safety risk.^355^ The mechanical mismatch between gasket and adjacent components (e.g., rigid BPPs or soft membranes) also induces edge stresses that may compromise the MEA.^356^ Furthermore, gaskets in PEMWEs are exposed to harsh chemical environments (acidic pH, high oxidative potentials near the anode) and elevated temperatures (∼60 to 80 °C). FKM and ePTFE have shown higher chemical resilience. However, degradation through radical attack, oxidative embrittlement, and hydration-induced swelling remain concerns, especially for long-term operation.^357,358^ Accelerated aging tests indicate that standard rubber gaskets may degrade within 5,000–10,000 h unless reinforced by nanomaterial fillers or cross-linking additives.^359^ Graphene-enhanced FKM composites, for instance, exhibit improved resistance to permeation and mechanical fatigue, extending operational lifetimes under equivalent load conditions. Sealing reliability is directly tied to safety in PEMWE operation, particularly under pressurized hydrogen generation. Minor imperfections in the gasket profile, surface roughness, or torque application can lead to microscopic leak paths. Over time, this compromises both gas purity and personnel safety. Sensor-embedded gaskets and pressure-sensitive assembly films are being investigated as diagnostics for early failure prediction. Other aspects under consideration to improve the operation of gaskets include machine-learning-guided design, sustainable gasket materials, and the development of standardized testing protocols for chemical and mechanical aging of gasket materials under PEMWE-specific environments.

## 7 Modeling

### 7.1 Introduction

Numerical modeling is a powerful tool in electrochemical research that is beginning to gain popularity in the electrolyzer literature.^289,360,361^ Models are differentiated by their dimensionality and by the treatment of the physical processes. The majority of the PEMWE models seen in the literature are 0D models.^362–385^ They provide limited insight into the phenomena occurring within a cell, as distributions of the potentials, reactants, and products are not available. Instead, they are typically used for parameter estimation or as part of a larger system model,^375–379^ to investigate bubble dynamics^380–382^ or for crossover modeling.^383–385^ A number of review articles have focused on low-dimensionality models;^28,361,386,387^ therefore, they will not be the main focus of this review. Multidimensional modeling is required in order to investigate a number of issues, such as flow-field design and sizing, catalyst utilization optimization, and analysis of the impact of PTL/CL interfacial effects and non-uniform compression. In the next subsections, the most relevant physical processes and governing equations used to develop PEMWE models are discussed in more detail. A summary of PEMWE modeling presented in the literature is listed in Table 6.^407^

**Table 6:**
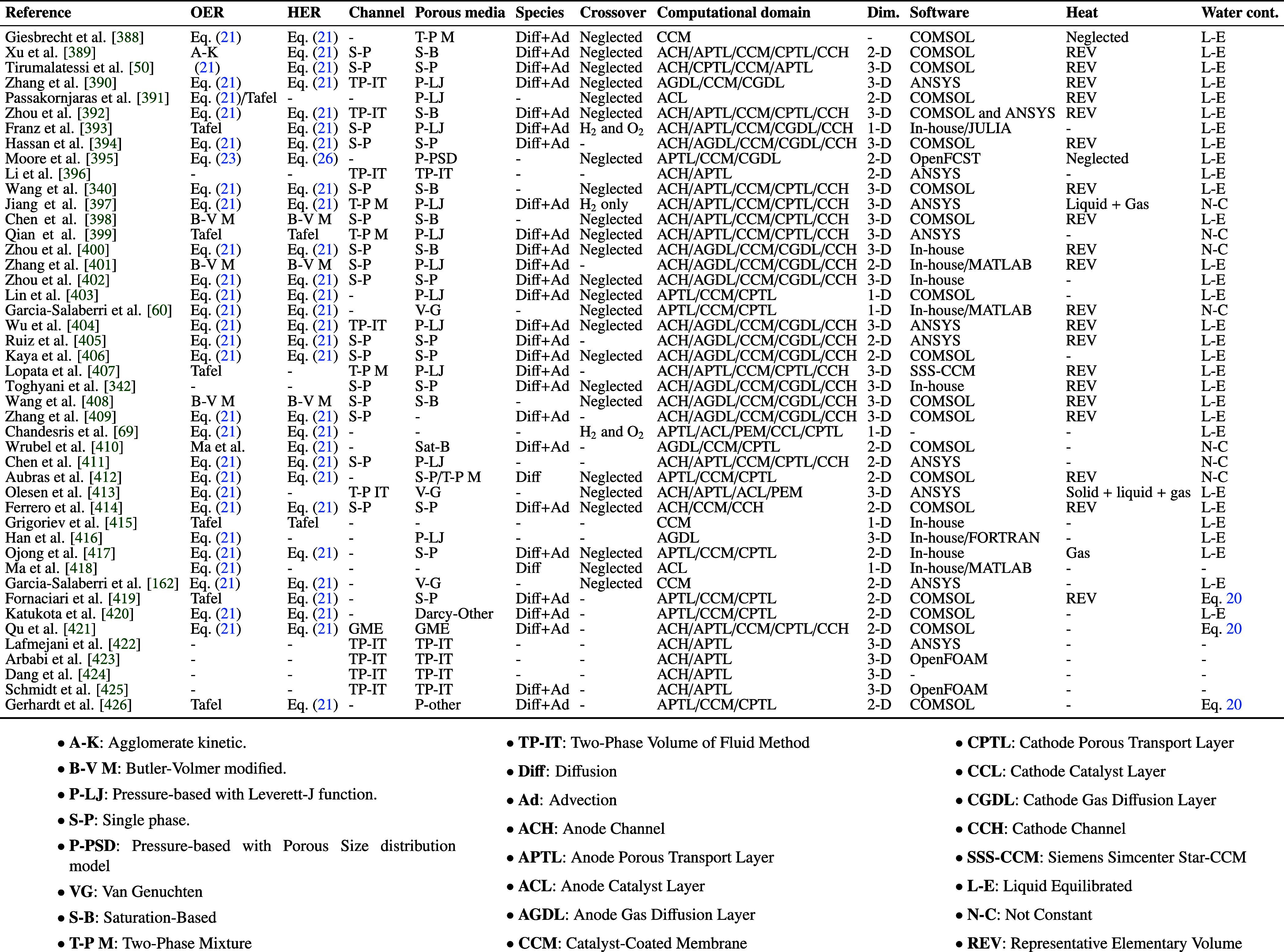
Summary of PEMWE Modeling Approaches

The aim of multidimensional numerical modeling is to identify the physicochemical processes in each cell component that limit performance and durability, and ultimately to lead to a computer-aided design tool for PEMWEs. There are a number of key physicochemical processes occurring in a PEMWE that contribute to performance losses revealed in the polarization curve and electrochemical impedance spectroscopy (EIS) spectra. In particular, the following phenomena are of interest: (i) the reaction kinetics in each electrode, (ii) the transport of electrons and protons in the solid and ionomer phase, respectively, and (iii) the transport of reactants (water) and products (hydrogen and oxygen). To predict these phenomena, the governing equations for the gas and liquid phase pressure and velocity, oxygen and hydrogen concentrations in each phase, electronic and electrolyte potentials, membrane water content, and temperature solution variables need to be solved concurrently, leading to a complex multiphysics, multidomain problem involving processes at varying temporal and physical scales, making it an extremely challenging problem to solve. Figure 25 summarizes the main solution variables in each domain. Most solution variables are coupled only via the catalyst layer, which can be fully resolved or treated as an interphase, depending on the complexity of the model.

**Figure 25:**
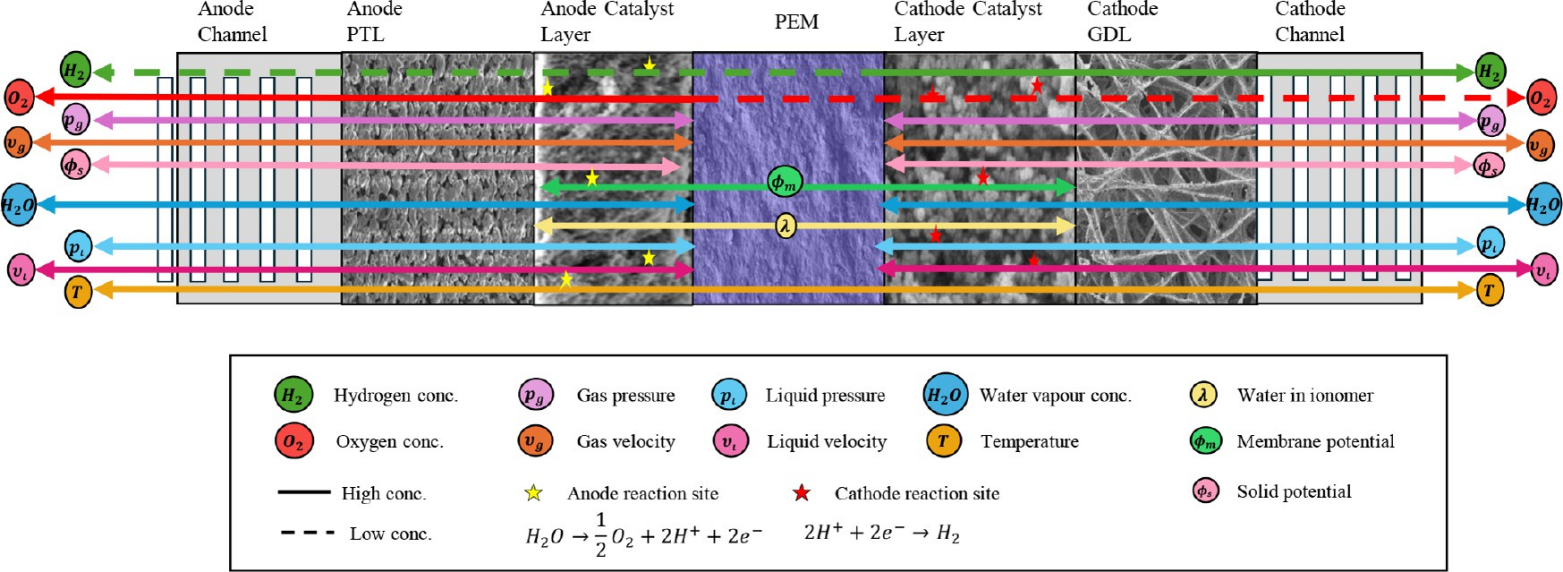
Schematic of the solution variables usually included in varying complexity proton exchange membrane electrolyzer models and their physical area of interest.

The following sections outline the key governing equations that are currently used to study each physical phenomenon, and discuss limitations and possible extensions.

### 7.2 Single-Phase, Single-Component Mass Transport

#### 7.2.1 Channel

Liquid water transport is usually considered in the anode channel of a PEMWE. Assuming that water behaves as an incompressible fluid, its velocity and pressure can be determined using the incompressible Navier–Stokes equations.^50,60,340,389–403,427–430^ These equations consist of the mass and momentum conservation equations, which govern the fluid dynamics in the channel. The mass conservation equation for a compressible fluid is given by

$$\frac{\partial \rho }{\partial t}+\nabla \cdot (\rho u)=S_{m}$$ 3

where *S*_m_ represents the mass loss from the liquid phase due to electrochemical reactions. Since the density, ρ, is assumed constant for incompressible flow, the first term ∂ρ/∂*t* vanishes, leading to the simplified incompressibility condition, ∇·**u** = *S*_m_, in terms of the velocity field **u**.

The momentum conservation equation describes the balance of forces acting on the fluid and is given by

$$\rho \frac{\partial u}{\partial t}+\rho (u\cdot \nabla )u=-\nabla p+\nabla \cdot \hat{\tau }+F+S_{u}$$ 4

where *p* is the pressure, **τ̂** is the shear-stress tensor, the term ρ­(∂**u**/∂*t*) accounts for transient effects, the convective term ρ­(**u** ·∇)**u** represents the non-linear transport of momentum due to fluid motion, and the external force term **F** accounts for additional forces acting on the fluid. In the channel, the external force term ***F*** is only the gravitational field, which is usually neglected, such that **F** ≈ 0. *S*_u_ represents the loss of momentum due to the loss of mass.

The stress tensor for incompressible Newtonian fluids is given by eq 5 ^431,432^

$$\hat{\tau }=2\mu \nabla_{s}u$$ 5

where μ is the dynamic viscosity of the fluid, and ∇_s_ = (∇**u** + ∇**u** ^T^)/2 is the (symmetric) gradient of the velocity field.

The cathode channel can either be fed with water, in which case the equations presented above apply, or have only an outlet. In the latter case, the compressible Navier–Stokes equations should be used for the governing equations of the gas phase, since large changes in density are likely. These changes are due to varying hydrogen-water vapor mole fractions with gradients in temperature and pressure, e.g., at high temperature and low pressure the mixture is likely to be mainly water vapor, while at low temperature and high pressure, the mixture would mainly contain hydrogen gas.

#### 7.2.2 Porous Media

Liquid and gas transport in porous media can be obtained by fully resolving the microstructure, as demonstrated by Schmidt et al.,^425^ or by using a volume-averaged model.^433^ A volume-averaged approach is often used, which introduces an additional friction term to account for the resistance imposed by the porous structure. When the microstructure is fully resolved, the incompressible Navier–Stokes or its simplified form assuming negligible convective effects, i.e., Stokes equations, are solved within the porous structure.

If a volume-averaged approach is used, the effect of the porous media is included by means of an external body force representing fluid-porous media interactions, and the field variables are modified to represent a volume-averaged superficial density, i.e., the mass per unit averaged-volume, and interstitial velocity, i.e., velocity inside the porous domain.^433^ A generalized form of the compressible Navier–Stokes equations was recently presented and validated by Jarauta et al.^433^ In short, if the density and velocity are defined as

$$\rho :=\{\begin{array}{ll} \rho & \mathrm{in}\;\Omega_{c}\\ \rho_{\mathrm{avg}}=\left\langle \rho \right\rangle =\frac{1}{V_{\mathrm{REV}}}\int_{V_{f}}\rho dV & \mathrm{in}\;\Omega_{p} \end{array}$$ 6a

$$u:=\{\begin{array}{ll} u & \mathrm{in}\;\Omega_{c}\\ u_{\mathrm{avg}}=\langle u\rangle^{f}=\frac{1}{V_{f}}\int_{V_{f}}udV & \mathrm{in}\;\Omega_{p} \end{array}$$ 6b

where Ω_c_ and Ω_p_ are the channel and porous media domains, respectively, then, the governing equations for single-phase transport in the previous section only need to be modified by adding the Darcy-Forchheimer drag force, i.e.,

$$F=-\mu K^{-1}\varepsilon_{V}u-\beta \rho |\varepsilon_{V}u|\varepsilon_{V}u$$ 7

where the first term corresponds to the Darcian contribution, which models the proportional relationship between velocity and the viscous resistance induced by the porous matrix,^434^ and the second term represents the Forchheimer correction, which captures non-linear inertial effects.^435,436^ The permeability tensor **K** characterizes the ability of a porous medium to transmit fluids, accounting for directional variations in permeability.^434^ The parameter ε_V_ represents the porosity of the porous media, i.e., the proportion of pore space occupied by the fluid.^437^ In the channel region, no porous resistance is present, and the drag force is zero.

The incompressible Navier–Stokes equations are challenging to integrate within a complete electrolyzer model as they result in a set of four, non-linear equations, which have to be solved concurrently with multicomponent mass transport equations, charge transport, and electrochemistry. Therefore, a number of simplifications are possible. In simulations including both channel and porous media, it might be reasonable to neglect inertial effects as long as the Reynolds number is low due to the slow flow in the channels. In this case, the equations above can be simplified to Stokes’ and Brinkman’s equations in channel and porous media by removing the convective term, i.e., ρ­(**u** ·∇)**u**, from eq 4. The use of these equations allows for a seamless coupling of channel and porous-media equations and eliminates the need for interfacial conditions, e.g., Beavers and Joseph interfacial condition.^438,439^

In cases where only the porous medium is to be resolved and the Reynolds number is of the order of unity or less, Darcy’s equation can be used such that there is a linear relationship between pressure and velocity, i.e.,

$$u=-\frac{1}{\mu \varepsilon_{V}}K\nabla p$$ 8

Considering that an explicit expression for the velocity is obtained, the equation above is usually replaced in the mass conservation equation, i.e., eq 3, in order to obtain an equation to solve for the fluid pressure, i.e.,

$$\frac{\partial \rho }{\partial t}-\nabla \cdot \left(\frac{\rho }{\mu }\hat{K}\nabla p\right)=S_{m}$$ 9

where **K̂** = (1/ε_V_)**K**. This simplified equation is linear and second-order in space, so it is ideally suited to be quickly solved using the finite element method in in-house software.^395^ As a rule of thumb, numerical models that are implemented using commercial software, such as COMSOL Multiphysics and ANSYS Fluent, tend to use the full mass and Navier–Stokes conservation equations,^50,394,402,440^ while open-source and/or in-house software tend to use eq 9 to reduce computational cost and enable the application of more complex structure–function properties, such as a saturation-dependent permeability.^388,395^

### 7.3 Single-Phase, Multicomponent Transport

The main transport mechanism for the produced oxygen and hydrogen is likely to be via convection of either gas bubbles or gas in a stable gas phase within the electrode at high current densities. However, considering that supersaturation is a necessary condition for bubble nucleation and growth,^441^ and that oxygen and hydrogen supersaturation values of around 100 times have been estimated to be necessary at a Pt electrode surface before nucleation,^442^ diffusive transport of gases in the liquid phase might also play a role, especially at low current densities and before the onset of nucleation, despite the fact that the electrolyzer is usually fed with gas-saturated liquid water.

Considering the low solubility of oxygen and hydrogen in water, the use of Fick’s law of diffusion in the liquid phase is justified to estimate oxygen concentration in the liquid fed anode before bubble nucleation begins, such that

$$\varepsilon_{V}\frac{\partial c_{i}}{\partial t}+\nabla \cdot (c_{i}u-D_{i}^{\mathrm{eff}}\nabla c_{i})=S_{i}$$ 10a

where *c*_*i*_ is the concentration of the dissolved species *i*, *D*_*i*_ is the gas species diffusivity in liquid water at the given temperature, and the source term *S*_*i*_ depends on the current produced in each CL, as discussed in Section 7.8.

The dissolved oxygen concentration can then be used to predict the most likely regions for the onset of bubble nucleation. Once bubble nucleation begins, however, a two-phase flow model is necessary that either tracks the transport of individual bubbles or the interplay between gas and liquid phases. Considering that convective and viscous forces dominate in the channel, models considering individual bubble transport are likely to be more suitable in this domain. In the porous media, capillary forces are likely to dominate in the nanopores, which might result in a more stable gas network. In this case, a volume-averaged approach to study multiphase flow might be justified.

It should be noted that, as shown in Table 6, several models use oxygen and hydrogen diffusion in either the liquid or gas phase (in many cases, the phase being solved is not clearly stated) as the major transport mechanism. This is unlikely, since diffusion will be the dominant transport mechanism for the produced hydrogen and oxygen. In the liquid-fed anode, it is more likely that either bubble transport or, if a continuous and stable gas network in the porous media is established, gaseous convection are the most likely transport mechanisms. In the cathode, fully humidified hydrogen exists in the gas phase, and therefore, convection is more likely to be the main driving force, instead of diffusion. It is the opinion of the authors that both gas and liquid phases should be solved for, and that oxygen (anode) diffusion should only be solved in the liquid phase to estimate the most likely regions for nucleation, while in the cathode, convection and diffusion should be solved in the gas phase.

### 7.4 Multiphase Mass Transport

Etzold et al.^443^ cited the need for more detailed mechanistic simulations of mass transport in PEMWE. Similarly, Lettenmeier et al.^444^ called for numerical modeling to elucidate the impact of mass transport limitations when high current density operation is used. Thus far, a number of numerical models have been developed that have investigated mass transport effects in PEMWE.^445^ In the channels, two-phase flow is usually analyzed using interface-capturing algorithms implemented in commercial software, such as the volume of fluid. In the porous media, due to the computational cost of interface-capturing algorithms and the increased role of capillary forces, volume-averaging methods are commonly used. These methods generally involve the solution of either one or two of the gas and liquid phase transport equations described above, i.e., either Darcy’s law or the Brinkman equation, in combination with either a transport equation for saturation or a closure equation relating capillary pressure to saturation.^406,412,414,416,417,420,421,446,447^ The single-phase equations are modified by including saturation dependent transport parameters, such as diffusivity, relative permeability, and electrochemical and gas exchange interfacial areas. These parameters can be estimated using pore-scale simulation approaches such as full morphology (FM) and pore network (PN) methods. As an example, Jung et al.^448^ used FM and PN to estimate the effective diffusivity vs saturation and relative permeability of a Ti-sinter and a carbon felt from their X-ray tomography images. Figure 26 highlights the different approaches that have been utilized to analyze multiphase effects in the porous media and channels and that will be reviewed below.

**Figure 26:**
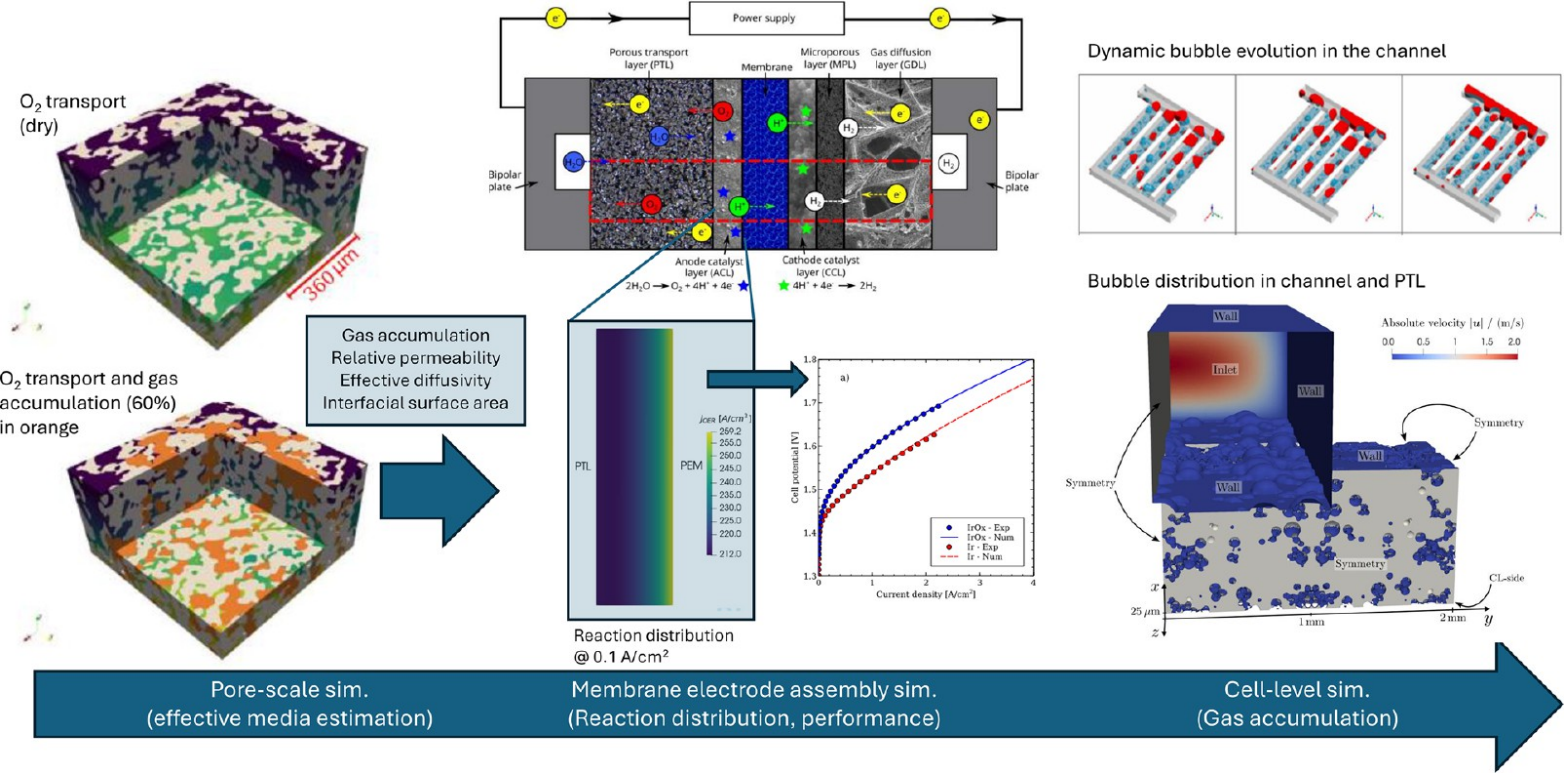
Schematic of different solution techniques to study multiphase flow in PEMWE. Adapted from refs 395,425,448 . Copyright 2021–24 The Authors under CC BY 4.0 International License (https://creativecommons.org/licenses/by/4.0/). Portions of the figure are also adapted from ref 392 with permissions from Elsevier B.V.

A number of researchers have attempted visualization studies to gain insights into the nature of the two-phase flow regime, which is crucial for the formulation of a mechanistic model. Neutron imaging,^449–452^ X-ray tomography^452–454^ and direct visualization^408,450,455–458^ have been used to investigate the nature of the water and oxygen mixture in a working PEMWE. In the channel, the most critical question is to establish the critical bubble size before detachment and, once detached, if it will form an individual bubble, a slug, i.e., a large drop that could block the channel completely, or an annulus which would displace the water away from the channel-porous media interface. In the channel, viscous forces cannot be neglected, and it is critical to solve the mass and momentum equations for the liquid and gas phases in order to predict the gas–liquid interface.

Several experimental studies have focused on understanding multiphase transport within the PTL and CL. Wang et al.^457–459^ used an etched PTL with straight-through pores to allow for the bubbles emerging from the anode CL to be imaged, allowing for comparison with those emerging from a PTL. The authors showed that bubbles emerging directly from the anode CL tend to be smaller than those from the PTL, allowing for easier removal from the channel and a reduction in the number of slugs. Seweryn et al.,^451^ Leonard et al.,^454^ and Zlobinski et al.^452^ noted that despite the hydrophilicity of the Ti PTL, a significant portion of the PTL is filled with oxygen even at low current densities. A gradient in water thickness builds up through the PTL at low current densities that is unaffected by changes in the operating current density, implying that a network of oxygen-filled pores is built that transports all of the oxygen out of the anode CL. Capillary forces are, therefore, thought to be the dominant transport mechanism. Arbabi et al.^453^ used a microfluidic cell to mimic the transport of oxygen out of the anode PTL and had similar conclusions. Based on these visualization studies, two-phase flow in PEMWE modeling articles is commonly described using capillary pressure models.

#### 7.4.1 Channel

In the channel, the movement of individual bubbles needs to be predicted, which involves analyzing the transport of the two phases individually and the surface tension forces that develop at their interface. There are several interface-capturing methods for two-phase flows, including volume of fluid (VOF), level-set, and Lagrangian-Eulerian techniques.^460–465^ In the area of PEMWE, the VOF method appears to be the most common method, e.g.,^396,404,422–424,428,466^ possibly due to its readily available implementation in commercial and open-source software despite the high computational cost, limited validation, and limitations in appropriately tracking the interface.^425,467^ Another approach commonly used is the use of a multiphase mixture model, described in ref 468. However, this approach does not account for bubble nucleation, growth, coalescence, and transport, which are dominant in the channel, and, as a result, it cannot predict the displacement of water from the channel/PTL interface. In Table 6, single-phase, two-phase using an interface tracking model, and two-phase using a mixture approach are noted as S–P, TP-IT, and TP-M, respectively.

There are many VOF implementations, as discussed in detail by.^460–462,467^ As an example, Schmidt et al.^425^ used the VOF method with the PLIC-RDF algorithm implemented in OpenFOAM software to track the evolution of gas bubbles through the channel and PTL of an electrolyzer. In this method, a phase indicator field variable, denoted as α, is introduced to determine the distribution of the two phases within the computational domain. This phase indicator field is governed by the following equation:^425^

$$\frac{\partial \alpha }{\partial t}+\nabla \cdot (\alpha u)=0$$ 11

where α is a scalar function that distinguishes between the two phases; when α = 1, the governing equations are solved for the liquid phase, and when α = 0, they are solved for the gas phase. If 0 < α < 1 the equations are solved at the interface. The velocity field, **u**, denotes the bulk velocity of the two-phase mixture used to advect the interface.

To account for interphase interactions, the governing equations for gas and liquid single-phase transport equations discussed above are solved independently, but an additional force term, **f**_S_, is introduced to account for surface tension effects. This force is commonly implemented using the continuum surface force (CSF) approach as discussed in refs 404,422,425,466

$$f_{S}=\sigma \kappa \delta_{S}n_{S}$$ 12

where σ is the surface tension coefficient, κ is the interface curvature, δ_S_ is the interface Dirac delta function, and **n**_S_ is the unit normal vector to the interface. The solution of the single-phase Navier–Stokes equations requires knowledge of the instantaneous phase indicator field variable, which is provided by solving the interface evolution equation above.

Flow patterns in the gas channel transition from bubbly to either Taylor, slug, plug or annular regimes with increasing current densities, as shown experimentally by Dedigama et al.^455^ and Li et al.^456^ Such a flow transition was studied using the VOF implementation in ANSYS Fluent by several authors.^390,392,404,422,424^ Lafmejani et al.^422^ demonstrated that their model effectively captures the gas–liquid contact angle on both the channel wall and the anode channel/PTL interface, with the latter being particularly important for accurately modeling bubble entrainment into the channel. Simulations from Dang and Zhou^424^ showed that annular flow obstructs the liquid water supply to the electrode surface, which may reduce PEMWE efficiency, and that bubbles disrupt liquid flow, resulting in a non-uniform velocity distribution and unstable pressure drops. Additionally, they showed that hydrophobic wall surfaces increase gas coverage and lead to greater oxygen accumulation inside the channel. Zhou et al.^392^ showed that a hydrophilic anode PTL increases the proportion of slug flow in the middle channels of the flow field, leading to lower bubble removal rates. Wu et al.^404^ and Zhang et al.^390^ studied the differences in flow patterns in parallel and serpentine flow fields, and at the outlet manifold of a cell, respectively.

A critical issue for these simulations is the coupling between fluid flow and electrochemical reactions. Lafmejani et al.,^422^ Dang and Zhou^424^ and Zhang et al.,^390^ for example, did not couple the fluid flow equations to electrochemical reactions, nor did they account for the PTL morphology. Instead, oxygen gas inlets were uniformly distributed at the back of the PTL to simulate the flow of oxygen generated from the electrochemical reaction. In an attempt to couple electrochemistry and two-phase flow in the channel, Wu et al.^404^ first used a single-phase channel model combined with a volume-averaged MEA model to estimate electrochemical performance and the oxygen evolution rate on the channel-PTL interface, which was then used as the oxygen inlet boundary condition in the VOF model. Then, the pseudosteady state bubble coverage was used as a boundary condition for the volume-averaged model.

Another critical issue with VOF simulations is the computational requirements. Usually, a trade-off needs to be established between computational cost and solution accuracy, due to mesh density and the method used for interface tracking. Nonetheless, the simulations are transient and computationally expensive. For example, Zhou et al.^392^ noted that “it takes 10 days to complete 1 s of simulation” with a mesh of approximately half a million cells, while Wu et al.^404^ noted that a VOF simulation took 48 h on 24 processors.

#### 7.4.2 Porous Media

Volume-average methods are commonly used to account for two-phase flow in the PTL and CLs. In the CL, small pore sizes result in representative elementary volumes (REVs) that are smaller than the computational cells used, and in small capillary numbers, thereby justifying the use of these methods.^469^ In the PTL, capillary forces still dominate; however, the larger pore sizes make REVs larger than computational cells, and a detailed microstructural simulation would be desirable.

For simulating CLs, two modeling approaches are commonly used: (i) saturation-based,^340,389,398,416,446^ and (ii) capillary pressure-based.^60,395,411,413^ The key difference between the two methods is the use of either a saturation solution variable, or both gas and liquid pressures as solution variables with a closure relationship to determine saturation, respectively. The former reduces the number of equations. However, it leads to a field variable that, considering the changing PSD between layers, should be discontinuous, which is difficult to handle for many numerical solvers, and in a formulation with boundary conditions that are difficult to impose since saturation at the channel/PTL interface is unknown as it depends on the operating conditions and channel flow rate.

In the capillary pressure-based models, mass and momentum conservation equations are solved for each phase. Considering the simplified approach described in the previous section, a combined mass and Darcy’s law equation can be used in each phase, i.e.,

$$\varepsilon_{V}\rho_{g}\frac{\partial (1-s)}{\partial t}-\nabla \cdot \left(\frac{\rho_{g}{\hat{K}}_{g}}{\mu_{g}}\nabla p_{g}\right)=S_{\mathrm{gas}}$$ 13a

$$\varepsilon_{V}\rho_{l}\frac{\partial s}{\partial t}-\nabla \cdot \left(\frac{\rho_{l}{\hat{K}}_{l}}{\mu_{l}}\nabla p_{l}\right)=S_{\mathrm{liquid}}$$ 13b

where the source terms *S*_*i*_ for each equation are based on the current produced in each CL (see Section 7.8), **K̂**_*i*_ is the permeability, and μ_*i*_ and ρ_*i*_ are the viscosity and the density of phase *i*, which is either water or oxygen. The permeability of the liquid, **K̂**_l_, and gas phase, **K̂**_g_, is either measured experimentally or estimated using, for example, a PSD model, as is discussed below. The viscosity, μ_g_, and density, ρ_g_, of the gas phase are computed using the mixture composition. Note that in fuel cell simulations involving two-phase flow, such as Zhou et al.,^469,470^ the evaporation and condensation of water need to be considered as additional source terms for the liquid and gas pressure equations. In PEMWEs, mass exchange between the gas and liquid phases must also be accounted for, especially at low pressure and when large gradients in temperature are observed, as the saturation water vapor pressure, and therefore the oxygen-water vapor ratio inside the bubbles, can change significantly.

Once the gas and liquid pressures are known, the capillary pressure can be obtained as the difference between the non-wetting and wetting phases, in this case gas and liquid, respectively. Then, a closure equation is used to relate capillary pressure to saturation. One such relationship is the Leverett J-function.^389–393,397,399,400,403,429,430^ Developed in the petroleum industry,^471^ adapted by the fuel cell modeling community,^472,473^ and applied to PEM water electrolysis modeling,^404,408,411,416,446^ the equation can be written as

$$p_{c}=\sigma \cos\;\theta {\left(\frac{\varepsilon_{V}}{K}\right)}^{1/2}J(s)$$ 14

where *p*_c_ is the capillary pressure, σ the surface tension coefficient, θ the contact angle, ε_V_ the porosity, and *K* the permeability. The J-function, *J*(*s*), is a function of the liquid water saturation, *s*, and is given by

$$J(s)=\{\begin{array}{ll} 1.417(1-s)-2.120(1-s{)}^{2}+1.263(1-s{)}^{3} & \mathrm{for}\;\theta <90^{{}^{\circ}}\\ 1.417s-2.120s^{2}+1.263s^{3} & \mathrm{for}\;\theta >90^{{}^{\circ}} \end{array}$$ 15

Han et al.^416,446^ used a saturation-based model with a Leverett-J function to investigate the impact of PTL pore size and contact angle on gas pressure and saturation in the PTL and at the anode PTL/CL interface. The mean pore size is included by using the Kozeny-Carman equation to estimate the absolute permeability, which in turn affects saturation via eq 14, which also depends on the contact angle. Chen et al.^411^ used the same Leverett J-function to study land/channel effects and liquid saturation distribution in the PTL. Wu et al.^404^ studied the oxygen content at the PTL/channel interface, which was obtained by solving a volume-of-fluid model in the channel only, with the resulting saturation being used as a boundary condition for the 3D model that captures the MEA. Wu et al.^404^ noted the importance of capturing the oxygen content in the channel, as the local current density and liquid saturation in the ACL were significantly affected. Wang et al.^408^ studied along-the-channel effects as well as performed a parametric study on operating conditions and PTL properties.

The Leverett J-function establishes a simple correlation between the capillary pressure and saturation; however, it is limited by the use of a single contact angle and that the microstructure is only characterized by the ε_*V*_/*K* term. The shape of the capillary pressure versus saturation equation is also dependent on the pore size and wettability distribution of the porous medium. For soil samples, it appears that the Leverett-J function expression captures the correct shape; however, CLs and PTLs exhibit a very different morphology from soil samples, and varying manufacturing and coating methods are used to obtain CLs and PTLs with varying pore-size distributions and mixed wettability behaviors. Therefore, more detailed methods are needed to estimate the capillary pressure versus saturation relationship. An alternative model to the Leverett J-function is the Van Genuchten model,^60,413,474^ where the breakthrough pressure of the porous medium, *p*_cb_, is used to characterize the microstructure instead of the permeability, e.g.,

$$p_{c}=p_{\mathrm{cb}}(s^{-1/m}-1{)}^{1/n}$$ 16

where *m* and *n* are empirical constants. Garcia-Salaberri^60^ used a capillary pressure-saturation correlation model based on the breakthrough pressure of the medium with van Genuchten’s equation. He found that concentration losses were negligible for the current densities studied.

The Van Genuchten model is still not capable of utilizing pore size and wettability distribution to estimate the capillary pressure versus saturation curve. Studies in the literature have shown that even the breakthrough pressure is affected by pore size and wettability.^475^ Therefore, either the capillary pressure versus saturation curve needs to be obtained for a given material, or an improved model is needed. Wrubel et al.^410^ used an experimentally obtained intrusion curve to directly relate capillary pressure to saturation. The authors noted the lack of data for intrusion curves corresponding to PEMWE anode CLs in the literature; therefore, the curves were obtained from carbon-based layers used in PEMFCs and were adjusted to yield a reasonable water uptake behavior for the IrO_*x*_ layer studied by the authors. The anode CL was resolved in their model, and they noted the dependence of the local current density on the anode CL liquid saturation.

The approaches discussed above lack the ability to capture complex microstructures and mixed wettability. When studying two-phase flow in fuel cells, Weber and Newman^476^ proposed the use of the PSD to establish a relationship between capillary pressure, saturation, and other effective transport properties of the porous media. This approach assumes that all pores described by the PSD are present at all locations in the porous medium, and transport through the medium is based on the interconnectivity of the pores, with the capillary pressure and contact angle being used to determine which of the pores are filled. Analysis of the log-normal PSD, along with the Young–Laplace equation to determine which pores are filled for a given capillary pressure, can provide expressions for quantities such as permeability, liquid/gas interfacial surface area, and saturation. In the case of saturation, it can be estimated considering two coexisting networks, one hydrophilic and one hydrophobic, using^469^

$$s=\sum_{i=\mathrm{HI},\mathrm{HO}}F_{i}\sum_{k}\frac{f_{i,k}}{2}\left[1+\xi_{i}\mathrm{erf}\left(\frac{\ln(r_{c,i})-\ln(r_{i,k})}{s_{i,k}\sqrt{2}}\right)\right]$$ 17

where the subscript *i* denotes hydrophilic and hydrophobic portions of the PSD, with fraction *F* of the total volume. The log-normal distribution is summed over modes, *k*, which are characterized by the standard deviation, *s*_*i*,*k*_, the modal pore radius, *r*_*i*,*k*_, and the fraction, *f*_*i*,*k*_, each mode contributes to the total distribution. These parameters are obtained by fitting to either experimental or numerically estimated pore-size distributions, as shown, for example, in ref 477. The critical pore radius, *r*_c,*i*_, is determined using the Young–Laplace equation and is the radius above which all hydrophobic pores are filled, and below which all hydrophilic pores are filled. Finally, ξ_*i*_ is one for hydrophilic pores and negative one for hydrophobic pores.

The pore-size distribution approach has been applied by many groups in fuel cells, e.g., refs 469,470,477–479 ; however, it was only recently applied to water electrolysis by Moore et al.^395^ to characterize the microstructure of the anode CL and PTL. The main advantage of this approach is that the effect of changing CL microstructural parameters in each layer can be easily assessed. For example, Moore et al.^395^ demonstrated that PTL saturation had a minimal effect on the anode CL saturation, due to their very different pore sizes. Moore et al.,^395^ in agreement with Garcia-Salaberri,^60^ also noted that concentration losses were negligible at current densities up to 4 A/cm^2^. It is worth noting that their models did not account for channel effects.

As previously discussed, the PTL has pores large enough that a volume-average approach might not be the most suitable. An alternative method for studying two-phase flow in PTLs is to fully resolve the porous media microstructure using micro-CT imaging followed by direct numerical simulation. Three simulation approaches are possible: (i) a fully resolved CFD simulation with an interface-tracking algorithm, as recently presented by,^389,396,425^ (ii) a pore-network model,^480^ and (iii) a morphological image opening method, e.g.^448^ The first method accounts for both viscous and capillary forces, facilitating the coupling of channel and PTL two-phase flow; however, due to the necessity to track the interface, it is computationally expensive. The latter methods take advantage of the small capillary numbers to reduce the computational cost by assuming the non-wetting fluid only enters pores at a given capillary pressure, so the simulations involve simple logistic operations, substantially reducing the computational cost; however, further work is needed to couple these simulations to the two-phase flow simulations in the channel where both viscous and surface tension forces are important.

### 7.5 Charge Transport

Charge transport is described in all PEMWE models using Ohm’s law

$$-C_{\mathrm{dl}}\frac{\partial (\phi_{e}-\phi_{p})}{\partial t}-\nabla \cdot (\sigma_{p}^{\mathrm{eff}}\nabla \phi_{p})=S_{H^{+}}$$ 18a

$$C_{\mathrm{dl}}\frac{\partial (\phi_{e}-\phi_{p})}{\partial t}-\nabla \cdot (\sigma_{e}^{\mathrm{eff}}\nabla \phi_{e})=S_{e^{-}}$$ 18b

where ϕ_e_ and ϕ_p_ are the electronic and ionic potentials, σ_e_^eff^ and σ_p_^eff^ are the electronic and ionic effective conductivities, and *S*_e^–^_ and *S*_H^+^_ are the source terms for the electronic and protonic phases, respectively. The capacitive term, *C*_dl_, accounts for the double-layer charging/discharging during transient operation. While Ohm’s law is universally applied, it is typically not applied to all the components of the cell and in many cases, electron transport is neglected, as will be discussed later. The source terms for charge transport arise from the electrochemical reactions in the CLs only, which are sources and sinks of protons and electrons. The reactions are discussed in greater detail in Section 7.8.

It should be noted that the use of Ohm’s law in the polymer electrolyte is an approximation based on several assumptions, such as only protons are transporting charge, the only positive cation in the ionomer is proton, and anionic functional group concentration is uniform and constant, which by virtue of electroneutrality, would then make the proton concentration also uniform and constant. These assumptions might be valid at the beginning of life. However, metal cations are likely to be produced in the cell, either due to catalyst dissolution or from other cell components. Therefore, despite the ubiquitous use of Ohm’s law to describe proton transport, the use of improved models, either based on concentrated solution theory or Nernst–Planck, would be beneficial.

When using Ohm’s law to describe charge transport, knowing the effective conductivities for each layer, along with the conductive phases, is critical. Due to the porous nature of the layers that comprise a PEMWE, these parameters need to be either measured experimentally^481^ or estimated from microscale simulations using accurate single-phase conductivities for each phase, if available (note that Nafion thin films exhibit different properties from bulk membranes and might depend on thin film thickness^482^).

The membrane and the PTLs are considered the main source of ohmic resistance in the cell, with less attention paid to the anode CL.^200,289,360,483^ The properties of the membrane have been studied extensively in the literature, as summarized by Ito et al.^484^ for the case of liquid water-equilibrated Nafion. Proton conductivity and its dependence on water uptake have also been well studied in the literature, with results compiled in the excellent review by Kusoglu and Weber.^17^ As the membrane is considered the largest source of ohmic resistance, it is usually accounted for by all authors. The majority use a temperature- and water content-based conductivity model,^60,69,404,408,410–414,416,417,446,447,485,486^ while others assume a constant conductivity considering the membrane is liquid equilibrated, as it is likely to be always exposed to liquid water.^395,406,415,420,421^ Of those that use a water content-based model, a minority have a water content that is not constant,^60,410–412,414^ with electro-osmotic drag and diffusion of water considered.

PTLs exhibit high electrical conductivity due to the good bulk Ti conductivity and the sintering process, which leads to good electrical contact between particles. Schuler et al.^294^ used X-ray tomography, image reconstruction and pore-scale modeling to compute the conductivity of sintered fibrous and powder PTLs and reported conductivities in the order of 105 S m^–1^. However, unless the PTL is coated with Pt, the conductivity might decrease over time due to surface passivation, affecting the interface with the anode CL.^150^ Rakousky et al.^487^ experimentally studied the degradation of PEMWEs and attributed 78% of the observed degradation rate to increasing ohmic resistance in the PTL. Despite its importance at later stages of PEMWE operation, in numerical models, the PTL conductivity is often not considered^412,415,421,447,486^ or is lumped into a single resistance with the CL and contact resistances.^406,414,420,449,485^ Several authors have resolved the PTL using conductivity values ranging from 12.5 to 1.3 × 10^4^ S cm^–1^.^60,69,395,404,411,413,417^

Ohmic losses in the anode CL will be incurred from both the protonic and electronic phases. Charge transport in the CL has been resolved in several articles, though charge transport within the anode CL is rarely a focus.^60,395,404,410,411,415^ For the protonic phase, some works considered a varying water content in the CLs when computing the protonic conductivity of the anode CL,^60,404,410,411^ while others considered a constant value, assuming the ionomer is liquid equilibrated. The appropriate model to use is still an open question; however, it appears unlikely that the formed bubbles would displace the liquid water film between the ionomer and the catalyst. Mandal et al.^481,488^ measured the protonic conductivity of an anode CL directly using the hydrogen pump method proposed by Iden et al.,^489,490^ and found that the protonic conductivity was of a similar order of magnitude to that of Pt/C layers used in PEMFCs and on the cathode of a PEMWE. The relationship between the ionomer loading and proton conductivity varied significantly depending on whether an Ir black or IrO_*x*_ was used, which may be an artifact of the measurement technique.^491^ Babic et al.^492^ measured the anode CL resistance using EIS; however, it was found to depend on the morphology of the PTL used, possibly due to variations in the water content of the ionomer phase when using each PTL.

In recent years, there has been an increasing focus on the electrical conductivity of the catalyst as a possible limiting factor in anode CL design, with Bernt et al.^150^ noting that the catalyst conductivity is often not taken into account in catalyst studies. Even when included in models, high constant values for the electrical conductivity are typically used, e.g., 3.5–50 S cm^–1^, such that electron transport is not limiting. Others, however, have used values that could be significantly lower, 1 × 10^–5^ and 0.2 S cm^–1^.^395,415^ It is possible, therefore, that both phases will impact PEMWE performance. Mandal et al.^481^ measured the through-plane electrical conductivity of an anode CL comprised of IrO_*x*_ catalyst, and showed that, depending on the ionomer loading, the IrO_*x*_ electrical conductivity could be two to three orders of magnitude lower than the protonic. Such a low electrical conductivity for IrO_*x*_ CLs explains the very low utilization of the anode CL observed by Mo et al.^493^ and the origin of the ICR with the PTL. The Ir oxidation state is likely to change the CL electrical conductivity based on the thermal annealing studies performed by Karimi and Peppley^494^ and Bernt et al.^150^ Therefore, care must be taken in the models to use an appropriate electrical conductivity for the catalyst. Schuler et al.^495^ showed a significantly higher conductivity for the TiO supported IrO_*x*_ (the catalyst used by Mandal et al.^481^ was unsupported). However, only the in-plane conductivity was measured. A three-order-of-magnitude difference in the electrical conductivity was observed between the in- and through-plane directions by Ahadi et al.^496^ for carbon-based CLs used in fuel cells, so the low conductivity of the IrO_*x*_ in the through-plane is not unreasonable. The electrical conductivity measured by Ahadi et al.^496^ can be applied to the cathode CL of a PEMWE, as they are similar to fuel cell CLs.

An interesting consequence of the lower electronic conductivity than protonic conductivities of the IrO_*x*_ catalyst measured by Mandal et al.^481^ is that most of the current density in the anode CL will likely be produced very close to the PTL interface. The impact of this reaction distribution has been discussed in the literature,^415,496–500^ and it may have a significant impact on how much of the CL is utilized. The idea of poor catalyst utilization caused by the poor electrical conductivity of the catalyst has gained interest in recent years,^493,496,501–504^ even though it is often in the context of the in-plane conductivity and a poor contact between the anode PTL and CL,^493,501,502^ with the impact in the through-plane direction only discussed qualitatively in refs 503,504 . and quantitatively in ref 505.

The interface between the anode CL and PTL has become an important area of research as the low conductivity of the anode CL results in areas far from the contact with the PTL being inaccessible to electrons. To date, the interface has been captured in the literature by simulating a single PTL pore and similarly sized PTL Ti fiber in contact with the anode CL. This method results in a domain in the order of tens of microns in the in-plane direction, with either the anode CL or CCM captured in the through-plane direction. Charge transport and the reaction kinetics are then simulated to understand how much of the pore space is utilized. Figure 27 from Garcia-Salaberri et al.^162^ demonstrates the interface and the poor utilization of the ACL that can result. The authors applied this method in their study and varied the PTL pore size, anode CL thickness, PTL intrusion into the anode CL, and anode CL conductivity. The importance of an MPL was highlighted as it can maximize the contact area, particularly when low loadings are used. Ma et al.^418^ employed a 1D model in the in-plane direction at the interface to investigate either electronic transport under the pore or gas transport at the contact point of the PTL on the anode CL. Various PTLs studied by Schuler et al.^501^ were simulated and the catalyst utilization in each region was predicted. Weber et al.^296^ used the 3D model from Wrubel et al.^410^ to study the etched PTLs studied by Zhang’s group^493,506^ and estimated the reaction and water distribution away from the contact points. Moore^505^ performed similar simulations in order to predict the conductivity of the anode CLs tested. Conductivities in the order of 1 mS cm^–1^ were required to electronically isolate the center of the pore in the PTL.

**Figure 27:**
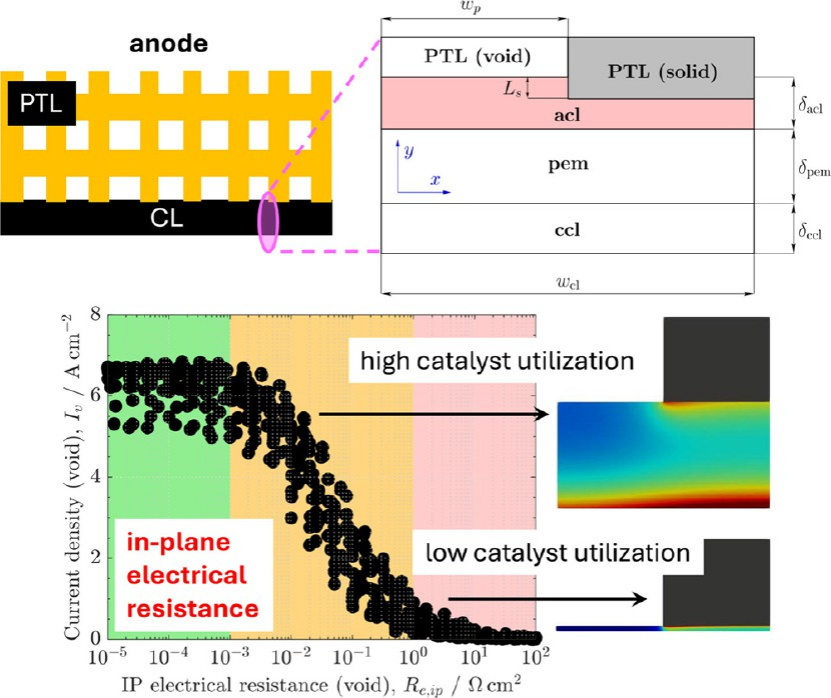
(Top) interface between the ACL and PTL, demonstrating that only part of the ACL is in contact with the Ti PTL. (Bottom) demonstration of the poor utilization of the ACL when the layer has a high in-plane electronic resistance. Thinner ACLs typically exhibit a higher resistance than thicker layers. Adapted from ref 162. Copyright 2025 The Authors under Creative Commons Attribution 4.0 License (https://creativecommons.org/licenses/by/4.0/).

On the cathode side, the porous transport layer and CL are the same as those used in PEMFCs and their conductivities have been studied in detail. In these layers, electrical conductivity is not limiting and the main concern is the proton conductivity in the CL. Estimates of CL protonic conductivity at varying ionomer loading and for different carbon supports were given by Iden et al.^490^ and Sabarirajan et al.^507^

### 7.6 Heat Transport

Thermal management is a critical aspect in PEMWEs, as temperature variations can significantly impact electrochemical reaction rates, gas solubility, transport parameters, and durability.^389–391,398,400,401,408,409,429^ Several studies have investigated thermal management in PEM water electrolyzers;^60,371,389,391,392,398,400,401,405,406,408,409,429,440^ however, to date, the importance of non-isothermal effects has not been clearly assessed. Therefore, attempts should be made to compare numerical predictions of isothermal vs non-isothermal models in order to clearly assess their importance.

The majority of models solve for heat transport in an REV, where effective properties account for contributions from both the solid and fluid mixture. Neglecting the heat capacity and conductivity of the gas phase, the energy conservation equation is given by

$$(1-\varepsilon_{V})\frac{\partial (\rho_{s}C_{p,s}T)}{\partial t}+\varepsilon_{V}\frac{\partial (s\rho_{l}C_{p,l}T)}{\partial t}+\varepsilon_{V}\nabla \cdot (s\rho_{l}C_{p,l}u_{l}T+(1-s)\rho_{g}C_{p,g}u_{g}T)=\nabla \cdot (((1-\varepsilon_{V})k_{s}+\varepsilon sk_{l})\nabla T)+S_{T}$$ 19

where *s* is the liquid saturation, ρ_s_, ρ_l_, and ρ_g_ are the densities of the solid matrix, liquid phase, and gas phase, respectively, *C*_p,s_, *C*_p,l_, and *C*_p,g_ are the specific heat capacities of the solid, liquid, and gas phases, **u**_l_ and **u**_g_ are the velocity vectors of the liquid and gas phases, *k*_s_ and *k*_l_ are the thermal conductivities of the solid and liquid phases, *T* is the temperature, and *S*_T_ is the volumetric heat source term.

Wang et al.^508^ developed a three-dimensional model to investigate the distributions of water and temperature within a PEMWE. Their study revealed that as the applied voltage increases, the temperature in all regions of the domain correspondingly rises. Moreover, an increase in the inlet velocity was found to boost liquid water saturation while simultaneously reducing the cell temperature. Subsequently, Xu et al.^389^ and Zhang and Xing^409^ estimated the optimal inlet liquid temperature. They noted that while higher temperatures can enhance the efficiency of an electrolysis cell, an exceedingly high temperature leads to overheating and excessive water evaporation. This evaporation reduces the membrane’s water content and ionic conductivity, potentially causing cracking and accelerated degradation. However, supplying water to the cathode side effectively mitigates this temperature rise. Therefore, it can be concluded that while higher temperatures enhance electrocatalytic performance, the operating temperature of the electrolyzer must be carefully controlled due to the risk of membrane degradation at excessive temperatures.^389,401,409,429,440,508^

### 7.7 Polymer Electrolyte Water Content

Electrolyte transport parameters, such as conductivity, water diffusivity, electro-osmotic drag coefficient, and permeability, depend on the water content in the polymer electrolyte. Considering that the PEMWE anode compartment is usually supplied with liquid water, several models have simply assumed a constant water content equal to that for a liquid equilibrated membrane, e.g., refs 50,389,390,395,401,508 . This assumption is supported by single-cell EIS results that show a constant HFR with varying current densities, e.g., refs 69,509,510 . There might be certain situations, however, where the polymer electrolyte, either in the membrane or the ionomer, might not be liquid equilibrated. These situations, easily detectable by a changing HFR with current density, could arise, for example, if oxygen gas accumulation displaces the liquid water away from the CL/PEM interface or if the cathode dries out because electro-osmotic drag, diffusion, and permeation cannot keep up with water evaporation.

The equations that govern water transport in PEMFCs and the relationship between transport parameters and water content are well established for membranes that are vapor equilibrated and are discussed in many previous publications which were recently reviewed in.^17,476,511^ One of the simplest governing equations is

$$\varepsilon_{m}\frac{\rho_{\mathrm{ion}}}{\mathrm{EW}}\frac{\partial \lambda }{\partial t}-\nabla \cdot \left(n_{d}\frac{\sigma_{p}^{\mathrm{eff}}}{F}\nabla \phi_{p}+\frac{\rho_{\mathrm{ion}}}{\mathrm{EW}}D_{\lambda }^{\mathrm{eff}}\nabla \lambda \right)=S_{\lambda }$$ 20

where ε_m_ is the ionomer volume fraction, ρ_ion_ is the electrolyte density, and EW is the equivalent weight. The variable λ represents the membrane water content, expressed in moles of water per sulfonic acid group, and ϕ_p_ represents the ionic potential. The term *n*_d_ corresponds to the electro-osmotic drag coefficient, σ_p_^eff^ to the effective ionic conductivity of the electrolyte, *D*_λ_^eff^ to the effective water diffusion coefficient in the electrolyte, and *F* is Faraday’s constant. *S*_λ_ is a source term accounting for water generation and consumption within the electrolyte. The equation can also be reformulated to account for water activity rather than water content, which better captures changes in electrolyte properties when transitioning from vapor- to liquid-equilibrated states.^512^

Weber et al.^296^ used the model developed by Wrubel et al.^410^ to study membrane dry-out under thin Ti sheets etched with holes in place of the PTL. Gerhardt et al.^426^ used the water activity-based model to study operation with a liquid water fed cathode, where water transport across the membrane is required to maintain the OER reaction rate. Fornaciari et al.^419^ also used an activity-based model to study water vapor fed electrolysis. The water balance across the membrane was predicted for different inlet relative humidities on the anode and cathode, and the limiting factor was shown to be the dehydration of the ionomer at high current densities. Understanding proton and water transport in the electrolyte when both liquid water and water vapor are in very close proximity, i.e., submicron scale, is critical.

### 7.8 Electrochemical Reaction Models

Activation losses are incurred by the HER on the cathode and the OER on the anode. At the cathode, Pt/carbon-based electrodes have been studied extensively in PEMFCs. Furthermore, the overpotentials incurred from the HER are significantly smaller than other losses, due to the fast kinetics of the HER on Pt catalysts.^508,513^ The evolution and oxidation of hydrogen on Pt catalysts have been studied experimentally^514–516^ and theoretically.^517–522^ The reaction mechanism is known to follow the Tafel-Heyrovsky-Volmer pathway.^522^

In contrast to the HER, the OER induces significant overpotentials and is a major source of performance loss. The highly corrosive nature of the anode where the OER occurs also causes significant catalyst and supporting material degradation, making the investigation of the OER a highly active area of research in PEM electrolysis.^76,523–529^ The mechanism for the OER is not well understood, with a number of mechanisms proposed though none are considered to be experimentally validated.^524,527,528^ The mechanism is complicated by the degradation of the IrO_*x*_ catalyst, which changes oxidation state at different working electrode potentials, some of which lead to dissolution.^530–532^ In addition, the changing reaction pathways may lead to a change in the Tafel slope, which has been observed experimentally,^533,534^ and should be considered in modeling works.

Almost all the PEMWE performance models in the literature used either the Butler–Volmer or Tafel equation to describe reaction kinetics in both electrodes.^69,406,412–416,420,421,446,447,449,485,486^ The focus in these works with regard to kinetics is primarily on the OER, as the HER does not cause significant potential losses. The Butler–Volmer equation can be written as

$$j_{k}=A_{v}j_{0}^{\mathrm{eq}}\left[{\left(\frac{c_{\mathrm{ox}}}{c_{\mathrm{ox}}^{\mathrm{eq}}}\right)}^{\gamma_{o}}\exp\left(\frac{\alpha_{a}F}{RT}(\phi_{e}-\phi_{p}-E_{\mathrm{eq}})\right)-{\left(\frac{c_{\mathrm{red}}}{c_{\mathrm{red}}^{\mathrm{eq}}}\right)}^{\gamma_{r}}\exp\left(\frac{-\alpha_{c}F}{RT}(\phi_{e}-\phi_{p}-E_{\mathrm{eq}})\right)\right]$$ 21

where *j*_k_ is the kinetic volume current density, expressed in A/cm_geo_^3^ per unit of CL volume. The exchange current density, *j*_0_^eq^. is expressed in terms of A/cm_surf_^2^ of specific catalyst surface area, while the specific surface area, *A*_v_, with units cm_surf_^2^/cm_geo_^3^, scales the computed current to the geometric volume. The transfer coefficients, α_a_ and α_c_, control the Tafel slope of the reaction rate, and *F*, *R* and *T* are Faraday’s constant, the Universal gas constant, and temperature. The potential of the protonic and electronic phases is denoted ϕ_p_ and ϕ_e_ and can be computed using Ohm’s law. The equilibrium potential, *E*_eq_, is computed based on the conditions used to obtain the exchange current density, in particular the concentrations of the reactants/products, which are the equilibrium concentrations, *c* ^eq^, in the Butler–Volmer equation.^535,536^ At high overpotentials, eq 21 can be simplified by removing either the anodic component (for the HER) or the cathodic component (for the OER) to obtain the Tafel equation.

#### 7.8.1 OER

When using the Butler–Volmer model for the OER, the exchange current density is typically used as a fitting parameter,^69,412–416,420,446,449,485^ as part of a parameter study^406,486^ or taken as a constant from another source,^417,421,447^ with Aubras et al.^412^ noting a range of exchange current densities in the literature that span several orders of magnitude, as expected considering the different activity of Ir-based catalysts reported in the literature.^532^ The transfer coefficient is given less attention, with some authors fitting it,^415,417,449^ or assuming a value, which may be taken from the literature,^412,414,421,447,486^ with values varying between one-half to two. There is little consensus as to the correct treatment of the OER kinetics; necessitating the use of a model that has been shown to reproduce kinetic data for the OER obtained from ex-situ experiments.

Tracking the oxidation states of Ir and their interactions with the reactants and products is complex, and a number of reaction mechanisms have been proposed. Microkinetic models are often developed during catalytic studies to better understand rate limiting steps.^501,534,537–544^ However, they are often applied to model electrodes, so parameters may need to be fitted for the CL under study. Oliveira et al.^537,538^ developed a model that considered O_ads_, OH_ads_, OOH_ads_, and H_2_O_ads_ intermediates and included an additional nanoscale model to account for anion adsorption. Garcia-Osorio et al.^539^ considered the same intermediates, plus O_2,ads_, for their model, which was developed to study the OER on “inactive” catalysts used in wastewater treatment. Ma et al.^534^ employed a simplified model that considered only O_ads_, using the same methodology as Wang et al.^545^ The model has only two intermediate reactions that were stated to include the formation of other species and can capture the change in Tafel slope observed experimentally. This model was implemented by Wrubel et al.^410^ and Moore et al.,^395^ although neither author focused on studying the impact of the change of the Tafel slope on cell performance.

The model by Ma et al.^534^ assumes a single intermediate species with two intermediate reactions and was used by the authors to capture the kinetics of the OER on Ir/Ru oxide core–shell catalysts. The model assumes the following intermediate steps

$$H_{2}O⇄O_{\mathrm{ad}}+2H^{+}+2e^{-}$$ 22a

$$H_{2}O+\frac{1}{2}O_{\mathrm{ad}}⇄O_{2}+2H^{+}+2e^{-}$$ 22b

from which the current density can be determined as

$$j_{\mathrm{OER}}=sA_{v}j^{*}(g_{+1}(1-\theta_{O})-g_{-1}\theta_{O})$$ 23

where the active area is given by *A*_v_ and the reference prefactor, j*, is used to scale the reaction. The coverage of the intermediate species, θ_O_, is given by

$$\theta =\frac{g_{+1}+g_{-2}}{g_{+1}+g_{-1}+g_{+2}+g_{-2}}$$ 24

and the *g*_*i*_ terms are

$$g_{+1}=\exp\left(-\left(\Delta G_{1}^{0}-\alpha_{1}e\eta \right)/kT\right)$$ 25a

$$g_{-1}=\exp(-(\Delta G_{1}^{0}-\Delta G_{\mathrm{ad}}^{0}+(1-\alpha_{1})e\eta )/kT)$$ 25b

$$g_{+2}=\exp(-(\Delta G_{2}^{0}-\Delta G_{\mathrm{ad}}^{0}-\alpha_{2}e\eta )/kT)$$ 25c

$$g_{-2}=\exp\left(-\left(\Delta G_{2}^{0}+\left(1-\alpha_{2}\right)e\eta \right)/kT\right)$$ 25d

where Δ*G*_*i*_^0^ are the activation energies of the intermediate reactions, Δ*G*_ad_^0^ are the adsorption energies of the intermediate species, α_*i*,f_ and α_*i*,b_ are the forward and backward transfer coefficients of reaction *i* respectively, η is the overpotential, and *k* is the Boltzmann constant.

#### 7.8.2 HER

Several models in the literature describe the hydrogen reaction^520–522^ beyond the standard Butler–Volmer model. However, the theoretical model from the group of Wang et al.^517–519^ captures the two pathways in the Tafel-Heyrovsky-Volmer mechanism. The model is applicable to both the HOR (hydrogen oxidation reaction) and HER, and can reproduce experimentally obtained polarization curves, while being relatively simple to implement. The model is given as

$$j=A_{v}j^{*}(g_{+T}(1-\theta_{H}{)}^{2}-g_{-T}\theta_{H}^{2}+g_{+H}(1-\theta_{H})-g_{-H}\theta_{H})$$ 26

where the intermediate adsorbed hydrogen coverage is denoted by θ_H_. The *g*_*i*_ terms express the activation barriers for the reactions

$$g_{+T}=\exp(-\Delta G_{+T}^{*0}/kT)$$ 27a

$$g_{-T}=\exp(-(\Delta G_{+T}^{*0}-2\Delta G_{\mathrm{ad}}^{0})/kT)$$ 27b

$$g_{+H}=\exp(-(\Delta G_{+H}^{*0}-0.5\eta )/kT)$$ 27c

$$g_{-H}=\exp(-(\Delta G_{+H}^{*0}-\Delta G_{\mathrm{ad}}^{0}+0.5\eta )/kT)$$ 27d

where Δ*G*_i_^*0^ are the activation energies of the intermediate reactions.

#### 7.8.3 Multiphase Transport Effects on Electrochemical Reaction

The inclusion of the liquid water saturation, *s*, in eq 23 accounts for the blocking of the active area by gas bubbles and is a common method used to induce mass transport losses.^390,410,413,446,546^ To increase the impact of bubble blocking on kinetics some researchers applied an exponent to the liquid water saturation term, *s* ^γ^, which is typically set to two.^60,395,404,411^ An alternative method is to use a concentration ratio and compute the concentration of water molecules at the reaction site. This method allows for the possibility of water vapor within a gas bubble maintaining the OER at a significantly reduced level. Another method for accounting for mass transport losses can be derived from 0D modeling, where the use of the Nernst potential is common.^416,417,447^ Aubras et al.^412^ suggested that it is probable that the catalyst is covered by the ionomer and, as a result, absorbed water is likely the reactant instead of either liquid water or water vapor. Therefore, the water content in the CL was used. Aubras et al.^412^ also considered how bubbles in the channels would affect the local water content. Garcia-Valverde et al.^375^ and Ojong et al.^417^ used a limiting current formulation to determine the mass transport losses in their 0D models, while Nouri-Khorasani et al.^382^ developed a bubble formation model to determine the stability of the bubbles in the anode CL and their impact on performance. Kadyk et al.^547^ also presented a bubble formation model; however, the focus was to determine likely locations for bubble nucleation in the electrode, with the aim of designing an electrode that minimizes bubble retention.

### 7.9 Crossover

Considering the flammability range of hydrogen is 4–95% in a pure oxygen atmosphere,^548^ estimating hydrogen and oxygen crossover through the membrane is critical for the safety of electrolyzer technologies. Thinner membranes increase gas crossover, creating a trade-off between minimizing ohmic losses and preventing the accumulation of an explosive gas mixture.^200^ At low current densities, the gas production rate becomes insufficient to dilute the crossover gas below the lower explosive limit, thereby imposing a minimum current density for safe operation. The importance of crossover is further increased by the goal of producing pressurized hydrogen in the cathode, thereby increasing the driving force for hydrogen crossover to the anode, as any electrochemical compression reduces the reliance on mechanical compression.^200,289,483^

Experimentally investigating the impact of gas crossover is challenging due to numerous safety concerns, such as handling high-pressure gases and mitigating the risk of forming an explosive mixture, as highlighted by Grigoriev et al.^549^ Consequently, numerical modeling is widely employed in this field. Several studies have explored high-pressure operation by measuring hydrogen content in the anode stream at lower cathode pressures and developing numerical models to estimate cell efficiency.^205,549,550^ In these studies, oxygen crossover to the cathode is typically regarded as having a smaller impact on Faradaic efficiency and posing fewer safety risks compared to hydrogen crossover,^551,552^ since oxygen diffusion through the membrane is slower than hydrogen diffusion.

Jiang et al.,^397^ Franz et al.,^393^ and Chandesris et al.^69^ are among the few that have implemented a crossover submodel within a multidimensional PEMWE model. Schalenbach et al.^205^ identified the following transport mechanisms: (i) diffusion of oxygen and hydrogen, (ii) electro-osmotic drag of dissolved oxygen and hydrogen due to the proton flux from anode to cathode, and (iii) permeation due to differential pressure. Several 0D models in the literature have also considered these three mechanisms.^205,550–555^ It should be noted that hydrogen and oxygen molecules are non-polar; therefore, it is only due to the water movement that hydrogen and oxygen move in response to the proton flux. One can think of differential pressure and electro-osmotic drag as two physical processes that contribute to the same mechanism, namely, the convective transport of dissolved hydrogen and oxygen through water transport from the cathode to the anode. It is surprising that water back-diffusion is not included in these models, as suggested by Chandesris et al.,^69^ even though it has been typically included in most PEM water transport models in the literature. This highlights that a closer inspection into crossover models is probably warranted as most models neglect at least one term. For example, Chandesris et al.^69^ included back-diffusion but not permeation.

Based on the three mechanisms proposed by Schalenbach et al.,^205^ Jiang et al.^397^ implemented the following governing equation in a multidimensional model

$$N_{H_{2}}=-D_{H_{2}}^{\mathrm{eff}}\nabla c_{H_{2}}+c_{H_{2}}\left(v_{H_{2}O}+\frac{1}{c_{H_{2}O}}\frac{n_{d}}{F}i_{p}\right)$$ 28

where *D*_H_2__^eff^ is the effective hydrogen diffusion coefficient, *c*_H_2__ is the local concentration of hydrogen in the electrolyte phase, **v**_H_2_O_ represents the water velocity vector resulting from a liquid pressure differential and, if desired, could also incorporate water back-diffusion, *n*_d_ is the electro-osmotic drag coefficient representing the number of water molecules transported per proton, *c*_H_2_O_ is the molar concentration of water, and **i**_p_ is the ionic current density vector. The first term in eq 28 represents the diffusion mechanism, where gases dissolve within the membrane’s liquid water pathways and polymer matrix, driven primarily by activity gradients or partial pressure differences between the two chambers.^393,552,553,555^ The second term corresponds to the bulk movement of water through the membrane, convecting hydrogen. The last term describes electro-osmotic drag, where water molecules migrating with protons toward the cathode carry dissolved hydrogen and oxygen along with them.^205,393,552,553^ It should be noted that evaluating water transport in the membrane is challenging, and an appropriate submodel to estimate **v**_H_2_O_ is crucial. An alternative approach is given by Chandesris et al.,^69^ who used an experimental water balance setup to estimate this value.

It should be noted that oxygen and hydrogen will exist in all three phases in the electrodes, i.e., gas, liquid and ionomer. A detailed gas crossover model should account for the dissolution rates between each of the three phases. Franz et al.,^393^ for example, included the transport of hydrogen in both the ionomer and gas phases, as well as an exchange term between them, while neglecting the transport in the liquid phase. As discussed by Franz et al.,^393^ transient effects can also be important, as the membrane can act as a short-term storage medium for dissolved gases. Figure 28 summarizes the direction of transport of hydrogen in each phase. It also shows Franz et al.’s^393^ predictions for the dynamic changes of hydrogen in oxygen at the cathode and dissolved hydrogen concentration when the current density changes from 4.5 to 1.63 A/cm^2^ using their one-dimensional model. The results show a significant overshoot in the hydrogen content in the anode due to the current density change, highlighting the importance of accurately capturing the concentration of dissolved species.

**Figure 28:**
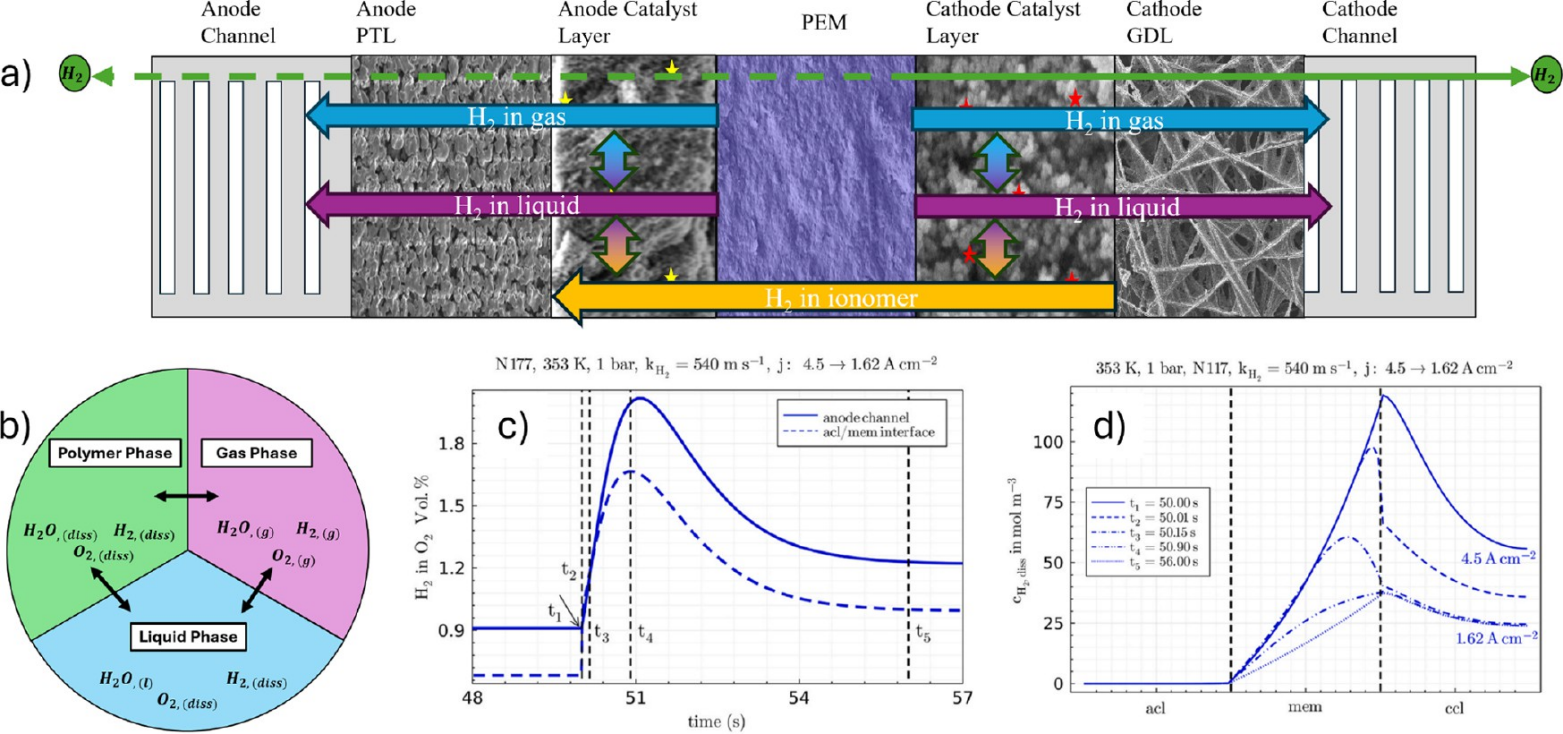
Schematic of the expected hydrogen transport direction in a PEMWE. (a) diagram of the expected gas phase exchanges, (b) example of the dynamic changes reported in ref 393 during a current density step change from 4.5 to 1.62 A/cm^2^, (c) hydrogen-in-oxygen content in the anode channel, and (d) 1D through-plane dissolved hydrogen concentration profiles. Reproduced with permissions from Elsevier B.V.

### 7.10 Degradation

The development of numerical PEMWE degradation models is crucial for a detailed analysis of the device’s lifetime, as well as for a quick estimation of lifetime under operating conditions. Nevertheless, modeling of PEMWE degradation is still in its infancy, particularly in the area of multidimensional modeling, the focus area for this review section. Notable examples include the membrane degradation models proposed by Chandesris et al.,^69^ and Frensch et al.,^252^ as well as the iridium dissolution model proposed by Kalinnikov et al.^556^

Numerous studies have experimentally investigated the degradation of PEMWE components, including catalysts, membranes, PTLs, and BPPs. These efforts have revealed a complex interplay of chemical, electrochemical, mechanical, and thermal degradation pathways that impact both performance and lifetime.^209,557^ Lifetime estimation of PEMWE components is crucial; however, it requires long and costly experiments. All components of a PEMWE could suffer from degradation. The PTL conductivity might be reduced due to the growth of an oxide/passivation layer, leading to increased ohmic losses.^305,558^ The CL might lose its activity due to catalyst oxidation and dissolution,^135,531,532^ and the membrane’s physical integrity and conductivity might be reduced due to chemical attack from hydrogen radicals of either the functional groups or even the backbone (chemical degradation) and due to thermal and/or humidity cycling (mechanical degradation).^69^ The performance of a PEMWE will change over its service life because of the aforementioned degradation mechanisms, so it is of paramount importance to develop numerical models to be able to estimate its useful lifetime.

In the area of membrane degradation, Chandesris et al.^69^ developed a one-dimensional PEMWE model that accounted for chemical degradation due to oxygen crossover. The model described oxygen crossover from anode to cathode, the formation of hydrogen peroxide at the cathode due to its low potential with respect to SHE, and the subsequent formation of radicals via Fenton reactions, which attack the polymer following the reaction described in ref 559. The coupled performance and degradation model was validated by predicting the fluoride release rate at varying current densities and temperatures. Both experimental and modeling results showed that degradation increased with temperature and occurred mostly on the cathode side. The membrane degradation model was then used to estimate the reduction in membrane thickness over time. This model was further extended in ref 252. to include temperature-dependent degradation rate kinetic parameters and validated by estimating fluoride emission. Despite the improved model, the authors also noted that their model underestimated the effect of temperature above 80 °C when compared to experimental data, highlighting that additional work is still required. Further work is also needed to account for other mechanical membrane degradation mechanisms discussed in Section 3.2, such as polymer creep.

In the area of catalyst degradation, attempts have focused on developing kinetic models that account for both reversible and irreversible catalyst degradation, however the models developed are generally 0D. Krenz et al.^560^ aimed at reproducing the observed reversible degradation in PEMWE^532,561^ by developing a simplified microkinetic ORR mechanism with two effective oxidation states of Ir. In agreement with experimental observations, they showed that periods at low potential result in performance improvement, highlighting the importance of distinguishing reversible from irreversible degradation when evaluating catalyst durability. Dominguez et al.^562^ developed a transient, 0D model to predict Ir dissolution rates based on whether Ir was in an oxidized state or had been reduced by crossover hydrogen. The model captured the buildup of hydrogen stored within the membrane during current cycling and used a multiscale temporal methodology to capture processes occurring at very different time scales. This method allowed the authors to predict increased Ir dissolution from hydrogen buildup in the membrane during cycling. Implementing models of this nature into higher dimensional models would allow researchers to more accurately predict local degradation rates during cell operation and therefore better predict cell lifetimes. A notable example is Kalinnikov et al.,^556^ who developed a model that incorporated Ir dissolution kinetics as a function of local OER overpotential, hydration, and mass transport within the CL. Their model revealed critical current density thresholds associated with water saturation, catalyst utilization, and gas diffusion behavior, and showed that improved activity often correlates with higher dissolution rates, as observed experimentally, for example, by Cherevko et al.^135^ Further work is needed in order to be able to develop a microkinetic model that includes the different states of Ir oxidation and that can predict, for example, the stability number for a given catalyst.^531,563^

## 8 Characterization

### 8.1 Introduction

Comprehensive characterization of both individual components and the integrated assembly is essential to optimize the performance, durability, and efficiency of PEMWEs. Characterization is needed to gain deeper knowledge about the fundamental properties of materials and interfaces, as well as for overcoming challenges in applied research and development. This section outlines the primary characterization techniques used in PEMWE studies, highlighting major insights and examples from the literature. Given the complexity of PEMWE systems, a multitechnique approach is necessary to characterize the diverse aspects of each component’s structure, composition, and performance, even for individual components. Characterization of integrated components poses additional challenges and typically requires an even broader range of methods and often demands extensive sample preparation.

### 8.2 X-ray Diffraction (XRD)

XRD is one of the most common methods used for characterization of crystalline bulk and nanoscale materials. The insights gained from XRD include phase identification, sample purity, crystallite size, and sometimes morphology. XRD patterns can be used to determine the sample’s composition using an extensive database of diffraction patterns for elements, compounds, and minerals. By comparing the pattern of an unknown compound to these reference patterns, one can verify the element’s identity by matching the diffraction peaks’ location, width, and relative heights. This is commonly used to determine the structure of synthesized materials, making XRD one of the most essential methods to confirm the structure of synthesized catalysts. As a bulk analysis technique, XRD data is often correlated with microscopy results to determine if observations from a small number of particles represent the entire sample.^564^ While XRD data for nanoscale materials can be straightforward to interpret, it is essential to understand the strengths and limitations, and account for the potential presence of amorphous phases, especially on the surface.^565^

XRD is an invaluable tool for characterizing catalyst powders, providing critical insights that support the optimization of catalyst performance. Figure 29a illustrates the XRD patterns used by Roiron et al.^566^ to evaluate the crystalline state of several commercial IrO_*x*_ catalysts. The XRD patterns of pure-phase rutile IrO_*x*_ display well-defined and distinctive peaks, characteristic of its tetragonal rutile structure, with prominent peaks at the (110), (101), and (211) crystal planes.^567^ In contrast, the amorphous IrO_*x*_ phase has a broad feature centered at 2θ = 34.3°, along with a smaller feature near 2θ = 60°. Only the broad amorphous contribution is observed for the amorphous samples from Tanaka Precious Metals (TKK) and Ishifuku (Ishi). However, the amorphous material from Furya (FIO) also shows evidence of an IrO_*x*_ rutile contribution in addition to the broad amorphous feature. This suggests that the FIO material consists of a mixed amorphous–rutile phase.^566^ The Alfa Aesar (AA) sample displays sharp peaks characteristic of Ir metal, indicative of its face-centered cubic (FCC) structure. Distinct peaks are observed at the (111), (200), and (220) crystal planes, with the (111) peak typically being the most intense.^567^

**Figure 29:**
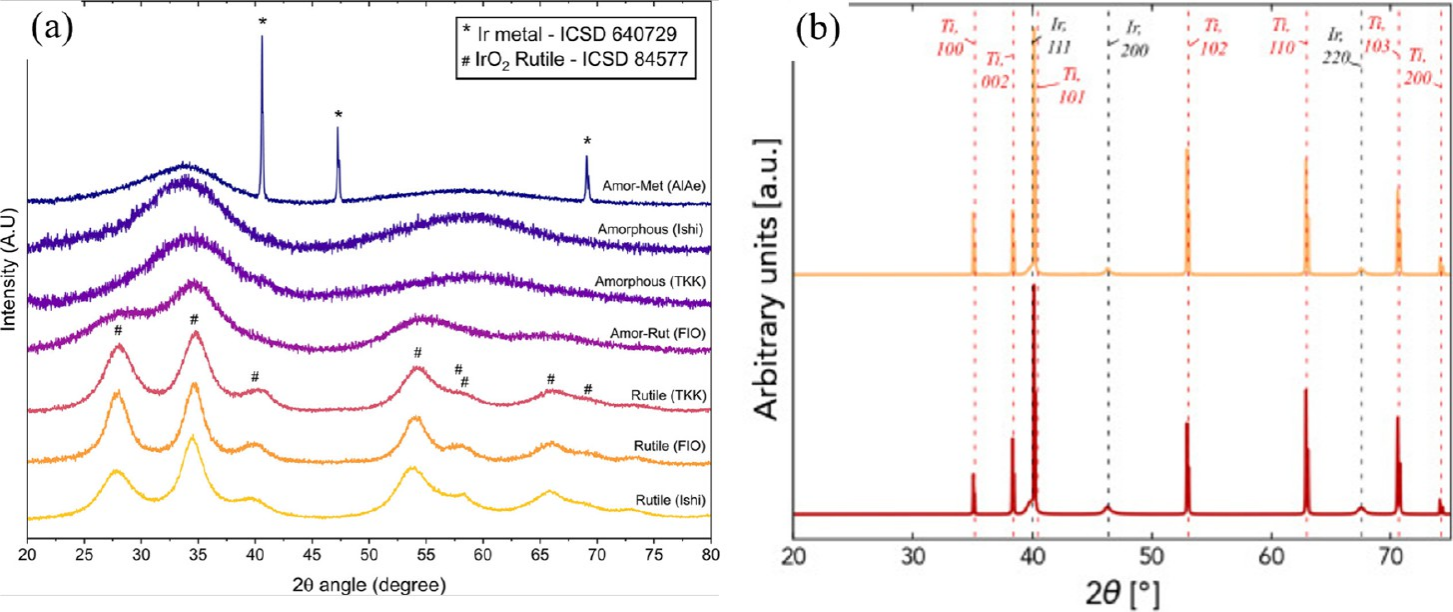
XRD patterns for (a) rutile, amorphous and amorphous-metal catalyst powders. Adapted from ref 566. Copyright 2024 The Authors under CC BY-NC-ND 4.0 International License (https://creativecommons.org/licenses/by-nc-nd/4.0/). (b) PTEs before (red) and after (orange) AST. Adapted from ref 130. Copyright 2023 The Authors under CC BY 4.0 International License (https://creativecommons.org/licenses/by/4.0/).

XRD is primarily used for catalyst characterization but also proves valuable for analyzing other PEMWE components, including BPPs, PTLs, CCMs, PTEs, MEAs, and PEMs, both before and after electrolysis. It enables assessment of degradation by monitoring changes in crystalline phases, crystallite sizes, orientation, and structural stability, and by quantifying phase composition.^93,568,569^ A study by Bystron et al.^570^ examined both pristine and etched PTLs focusing on the evaluation of the extent of Ti passivation of individual Ti felt wires. The Ti felt wires exhibited Yttrium (Y) impurities near the surface most likely originating from the Ti felt production process. The spectrum of the pristine PTL showed diffraction peaks corresponding to Ti and Y_2_O_3_. After etching, the spectra revealed the disappearance of Y_2_O_3_ peaks and the emergence of additional peaks attributed to various Ti hydrides, alongside the remaining Ti signals. This work demonstrated the effective removal of the passivation layer with the formation of a significant hydride phase at the (sub)­surface.^570^ Lee et al.^130^ conducted a study comparing the XRD patterns of ionomer-free Ir PTEs before and after AST. The analysis revealed negligible differences between the two, suggesting that the bulk phase of the PTE remained structurally intact throughout the AST (Figure 29b).

Another example is the analysis of MEAs to investigate the effects of anode catalyst oxidation on PTLs and effectively track corrosion pathways from the anode to the cathode through the membrane. This was accomplished by observing changes in peak intensity or broadening.^571^ For BPPs, XRD can evaluate coating effectiveness by assessing its impact on corrosion resistance and electrical conductivity, while also identifying phase changes during operation.^569^ XRD has also been used for PEM characterization, though less commonly due to the membrane’s primarily amorphous nature. For PEM, XRD is mainly applied after electrolysis to assess interactions between the membrane and the catalyst or PTL.^568^

### 8.3 Thermogravimetric Analysis (TGA)

TGA assesses material behavior by measuring weight changes as a function of temperature or time in a controlled atmosphere. The weight changes, which can be detected to a fraction of a microgram, are linked to alterations in the material’s chemical or physical properties, providing insights into the material’s thermal stability and composition.^572^ TGA is straightforward and cost-effective. It enables qualitative component analysis based on thermal stability differences, compositional analysis from weight loss across temperature ranges, and characterization of microstructural transformations through curve variations and peak temperatures in derivative curves.^34,573^ TGA is used to observe physical changes in the sample, such as gas absorption/desorption, phase transitions, vaporization, and sublimation, and chemical changes, including decomposition, molecular chain breakdown reactions, gas reactions, and chemically driven absorption. TGA also estimates various factors, including solvent and water loss, decarboxylation, pyrolysis, oxidation, and decomposition processes, providing compositional insights and weight percentage analyses.^574^ The first derivative of the curve, known as derivative thermogravimetry (DTG) can be taken to determine the temperature at which significant mass loss occurs in materials during TGA. DTG is primarily used to determine the precise decomposition temperature of a material, identify multiple degradation stages, and compare the relative rates of mass loss in different samples.

TGA is useful for the investigation of the initial water content in the catalyst and the loading of the catalyst in the CCMs. The catalyst loading can be calculated by measuring the weight difference of the membrane before and after the catalyst coating.^576^ Additionally, TGA can be employed to evaluate the stability of the catalyst under different environments. A study by Tovini et al.^577^ reported temperature ramp TGA experiments to compare the reduction temperature ranges of various reduced IrO_2_ catalysts within the IrO_2_/TiO_2_ catalyst. While this approach provided rapid insights, it was found to be insufficient for predicting the long-term stability of the IrO_*x*_ phase under operational conditions. To address this limitation, Tovini et al.^577^ also examined the reduction behavior of the IrO_2_ phase within the IrO_2_/TiO_2_ catalyst using isothermal experiments. These tests were performed at 80–240 °C over one hour and over the duration of this experiment, the reduction of the IrO_2_ phase was evident at temperatures as low as 80 °C, with significantly faster reactions at 160 and 240 °C. In a similar fashion, TGA can be used to characterize the thermal stability of PTLs. Lee et al.^308^ used TGA to show a significant oxidation of Ti above 300 °C in air.

One of the most common uses of TGA is the evaluation of the thermal stability and degradation behavior of the PEM. Figure 30 presents the TGA and DTG curves for commercial Nafion 117 and Nafion membranes made with 2.5, 5, and 7.5 wt % of ZrP nanoparticles.^578^ Sigwadi et al.^578^ demonstrated that the Nafion 117 membrane exhibited thermal stability up to 240 °C, whereas the nanocomposite membranes exhibited enhanced stability, showing thermal stability up to 340 °C with three distinct weight-loss stages. The initial weight loss occurs at a low temperature, around 100 °C, corresponding to the desorption of water bonded to the sulfonic groups. The second weight loss occurs at an intermediate temperature and begins at 380 °C, attributed to the degradation of the sulfonic groups. Notably, Nafion 7.5 wt % ZrP membrane showed this second weight loss at 490 °C. The third weight loss, occurring at a high temperature between 700*–*900 °C, is associated with the degradation of the polymer backbone.^578^

**Figure 30:**
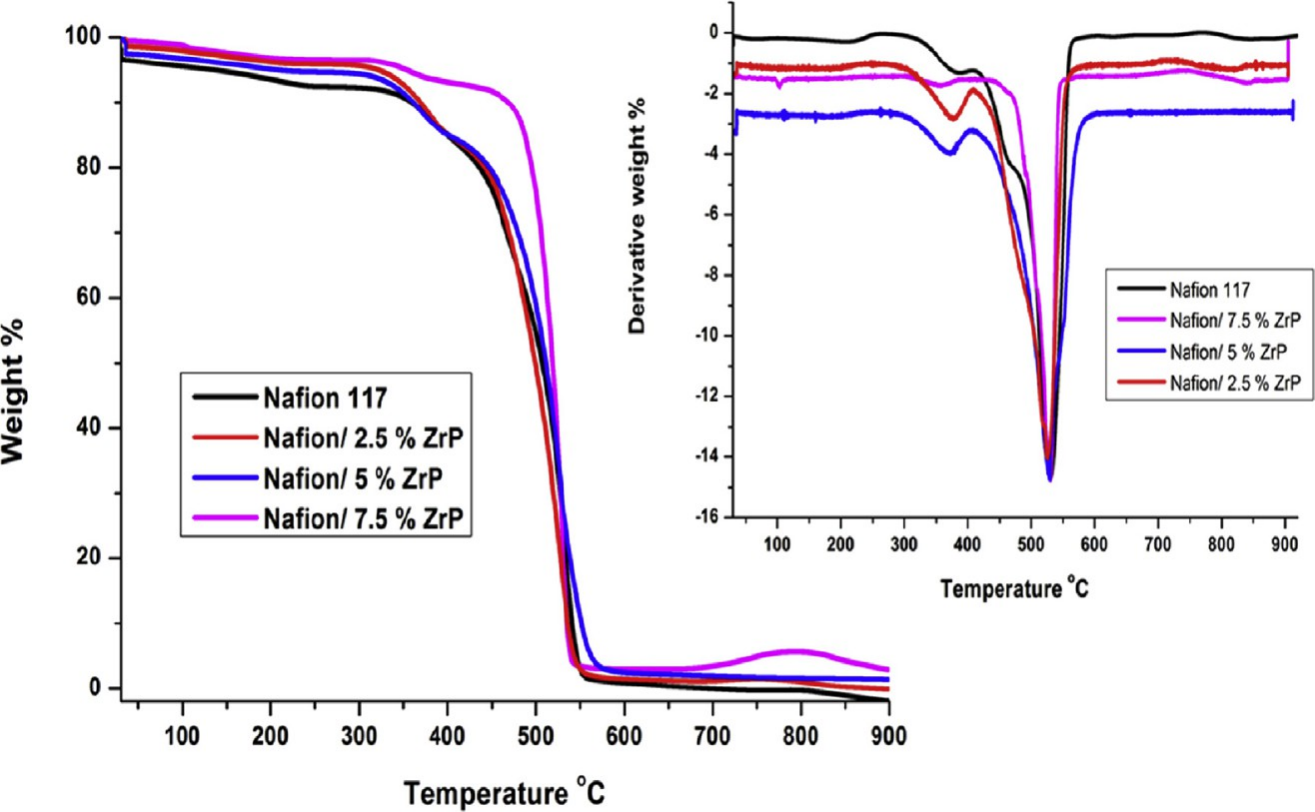
TGA and DTG of Nafion 117 membrane and its ZrP nanocomposite membranes. Reproduced from ref 578. Copyright 2019 The Authors under CC BY-NC-ND 4.0 International License (https://creativecommons.org/licenses/by-nc-nd/4.0/).

### 8.4 Inductively Coupled Plasma Mass Spectrometry (ICP-MS)

ICP-MS is mainly used in catalyst research for trace elemental analysis due to its high sensitivity, precision, and multielement capability. During analysis, samples are ionized by the plasma and transferred to the mass analyzer through a vacuum interface. Once separated, ions are detected by the ICP-MS detection system, enabling precise elemental analysis and data interpretation.^579^ Online ICP-MS enables real-time, potential- and time-resolved monitoring of dissolution rates during electrochemical operation. In contrast, scanning flow cell (SFC)-coupled ICP-MS systematically analyzes dissolution rates, enabling high-throughput screening of electrocatalytic materials. While online ICP-MS captures dynamic electrochemical changes, SFC-coupled ICP-MS provides controlled, steady-state evaluation, making each method valuable for different research applications, including electrolyzer contamination, PEM degradation, and ion migration within the system.

ICP-MS is widely used to benchmark catalysts by evaluating their stability number, a metric derived from the ratio of oxygen production rate (determined from current) to Ir dissolution rate (measured via ICP-MS). To achieve a more comprehensive understanding of catalyst stability and degradation mechanisms, Zlatar et al.^580^ investigated catalyst dissolution using a SFC system coupled downstream to an ICP-MS, enabling detailed assessments of activity and stability for single-atom Ir and highly dispersed Ru catalysts supported by indium-doped tin oxide, compared to commercially used catalysts. While these catalysts exhibited 3–5 times higher mass-normalized activities, their lower stability (approximately 10-fold) remains a critical limitation. This trade-off poses a significant challenge for long-term use in PEMWE, particularly under the harsh anodic conditions required for sustained operation.^580^

Cho et al.^86^ examined the dynamic interplay between dissolution and redeposition under various electrochemical conditions, using commercial Ti PTLs, both with and without Pt and IrO_2_ coatings. This study revealed significant Ti dissolution for uncoated PTLs, strongly influenced by pH and applied potential. Online ICP-MS analysis of Pt- and IrO_2_-coated Ti PTLs demonstrated a significant reduction in Ti dissolution compared to bare Ti PTLs. Over the course of the protocol, Ti dissolution amounts were recorded at 125 ng gTi^–1^ for the uncoated PTL, while Pt- and IrO_2_-coated PTLs exhibited substantially lower values of 39 and 4 ng gTi^–1^, respectively. These findings confirm that surface coating strategies are essential for mitigating Ti dissolution in PEMWE operation while also enhancing electrical conductivity.^86^

Building on the insights from Cho et al.^86^ regarding Ti dissolution and the effectiveness of surface coatings, Prado et al.^581^ extended the investigation to corrosion behavior in BPPs. Using electrochemical techniques, including potentiodynamic and potentiostatic polarizations, followed by weight-loss measurements and ICP-MS analysis of the electrolyte composition to assess the release of the ions into the electrolyte. Comparison between weight loss and metal release data from ICP-MS confirmed that, in all cases, corrosion involved both insoluble solid corrosion product deposition and soluble metal cation formation.^581^

### 8.5 X-ray Absorption Spectroscopy (XAS)

XAS is extensively used in material science to determine element-specific electronic and local geometric structures. XAS involves the excitation of a core electron, typically a 1s (K edge) or 2p (L_3_ and L_2_ edges) electron, which has element-specific energies. To measure transition metals and atoms with higher atomic numbers in solution, X-ray energies greater than approximately 3 keV are required. XAS measurements are primarily conducted at synchrotron beamlines, where synchrotron radiation is produced by deflecting high-velocity particles along a curved trajectory using a magnetic field. The X-ray energies produced can span 1,000 eV or more, which is necessary for an XAS spectrum.^582,583^ The increased accessibility of XAS, coupled with advancements in X-ray optics, detectors, and user-friendly software, has established XAS as a crucial tool for contemporary research in chemistry and catalysis, enabling a wide range of experiments, including studies under in-situ and operando conditions.^584^ Recent advancements in benchtop spectrometers have made XAS also more accessible, but usually for screening materials and not thorough investigation under reaction conditions.^585^

An XAS spectrum includes two distinct regions: X-ray Absorption Near-Edge Structure (XANES) and Extended X-ray Absorption Fine Structure (EXAFS). These spectra are processed independently: XANES provides information about the oxidation state and d-band occupancy, while EXAFS, which involves higher energy regions of the spectrum, reveals details about the local chemical environment around the absorber atom, such as coordination number, bond distances, and inner potential corrections.^582^ XAS is a powerful technique for exploring various components in the PEMWE but is mainly used to study catalysts to determine the oxidation states and reveal detailed information about the local atomic structure around the absorbing atom, including bond lengths, coordination numbers, and the types of neighboring atoms.^586,587^ Importantly, XAS allows one to monitor changes in these parameters during cell operation.

In a study by Diklić et al.,^588^ ex-situ XAS was employed to assess the average oxidation state of Ir in four different IrO_*x*_ catalyst powders, including AA, Umicore (Umi), and catalyst synthesized in-house, before and after heat treatment (AS and HT). By performing complementary XAS measurements on reference Ir compounds spanning oxidation states from +3 to +6, the researchers established a linear correlation between oxidation state and the white line (WL) maximum position in their XANES spectra. This approach allowed determining bulk Ir oxidation states of +3.9 for IrO_*x*_ AA and +4 for IrO_*x*_ Umi. Additionally, operando modulation excitation (ME)-XAS analyses (using a spectroelectrochemical flow cell) provided insight into oxidation behavior under operational conditions, the electrochemical conditioning at 30 mA cm^–2^ until potential stabilization (≈ 5 min), significantly enhancing performance. Figure 31b1–b4 presents the operando XANES spectra for all four IrO_*x*_ catalysts at 1.00 and 1.60 V_RHE_, illustrating their oxidation state evolution under OER conditions following the conditioning process. These spectra reveal that all IrO_*x*_ catalysts undergo surface oxidation before the onset of the OER. Furthermore, the magnitude of this shift, and the corresponding oxidation extent, varies significantly among the catalysts. This can be quantified using the difference between spectra acquired at the two potentials (the so-called “Δμ spectra”), as shown in Figure 31b5. Notably, the shape of these difference spectra remains consistent across all materials, indicating that Ir undergoes similar oxidation-related changes in all four catalysts. However, the substantial variation in Δμ spectral magnitudes suggests a greater oxidation extent for IrO_*x*_ AS and IrO_*x*_ AA compared to IrO_*x*_ HT and IrO_*x*_ Umi samples. Most importantly, Figure 31b6 demonstrates a correlation between this ex-situ variable and the oxidation extent (Δμ_max_). This relationship reinforces the idea that these groups (μ_2_-O, μ_3_-O, and μ_2_–OH established by Mom et al.^589^) play an integral role in the charge storage process. The enhanced surface sensitivity of XAS, coupled with reference compounds exhibiting oxidation states of +5 or higher, enabled the identification of Ir^5+^ as the dominant oxidation state. This study underscores the capability of XAS to probe oxidation dynamics and structural evolution, offering critical insights into catalyst performance and stability.^588^

**Figure 31:**
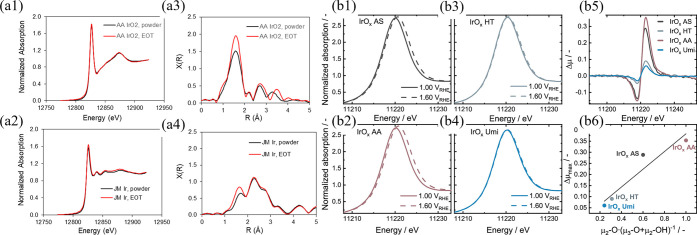
(a) Ir L_2_ XANES (left) and Ir L_2_ FT-EXAFS (right) spectra for catalyst powders and anodes from cycled MEAs (a1 and a3) AA, (a2 and a4) JM. Adapted from ref 588. Copyright 2024 The Authors under CC BY 4.0 International License (https://creativecommons.org/licenses/by/4.0/). XANES spectra acquired at 1.00 and 1.60 V_RHE_ on (b1) IrO_*x*_ AS, (b3) IrO_*x*_ HT, (b2) IrO_*x*_ AA, (b4) IrO_*x*_ Umi. (b5) Difference of the spectra acquired at 1.60 and 1.00 V_RHE_ (Δμ) and (b6) relation between Δμ_max_ and the ratio of oxygen species extracted from the O K edge XAS measurements. Adapted from ref 587. Copyright 2023 The Authors under CC BY 4.0 International License (https://creativecommons.org/licenses/by/4.0/).

Pfeifer et al.^590^ employed XAS to investigate how electronic defects influence IrO_*x*_ performance. A key finding from the study was the identification of O 2p hole states in the O K-edge spectrum, which form alongside Ir^3+^ species. These defects were linked to improved catalytic activity, suggesting that near-surface electronic modifications enhance OER efficiency. XAS comparisons between amorphous and crystalline IrO_*x*_ revealed that the X-ray amorphous phase exhibited higher catalytic performance than its rutile-type crystalline counterpart, attributed to increased surface defects that improve reaction kinetics.^590^ These insights contribute to a deeper understanding of IrO_*x*_ catalyst behavior, supporting ongoing efforts to develop more efficient materials for PEM water electrolysis. While XAS offers valuable insights into the oxidation state and local atomic structure of catalyst powders, its application extends to MEA investigations, where operando measurements reveal how catalyst oxidation states evolve under real electrochemical conditions. These structural modifications directly influence the catalysts’ ability to sustain activity and durability over time. Alia et al.^591^ analyzed Ir L_2_-edge XAS to determine the oxidation state of Ir in cycled MEAs. Figure 31a1–a4 presents the XANES spectra and the Fourier transform (FT) of the EXAFS for two postcycling anodes, alongside their corresponding catalyst powders, AA IrO_*x*_ and JM Ir. The peak height in the XANES region serves as an indicator of Ir oxidation, as does the intensity of the scattering peak at ∼1.5 Å in the FT which corresponds to Ir–O interactions. Additionally, a peak at ∼2.3 Å in the FT is attributed to Ir–Ir scattering in metallic Ir. Based on both the WL intensity and the magnitude of the ∼1.5 Å scattering peak, incorporation into the MEA and subsequent cycling induced slight oxidation in both AA IrO_2_ and JM Ir. The XANES regions of the Ir L_2_ edge absorption data revealed that after cycling the Ir in the AA IrO_*x*_ anode has a higher extent of oxidation compared to JM Ir.^591^

XAS is also well-suited for studying complex catalysts, such as RuIr alloyed oxides, to probe their electronic structures. For example, in a study by Huang et al.,^587^ the introduction of Ir into metal oxides was investigated to understand its impact on electronic structure and coordination environment. The Ir L_3_-edge XANES spectra revealed that the WL intensity of Ir_0.5_Ru_0.5_O_*x*_ is significantly higher than that of IrO_2_, indicating a higher oxidation state of Ir in the alloyed oxide. This higher WL intensity is associated with the valence state. The Ir L_3_-edge FT-EXAFS spectra revealed that the Ir–O coordination number for Ir_0.5_Ru_0.5_O_*x*_ is quite similar to that of IrO_2_. This suggests that neighboring Ru atoms regulate the Ir valence state through an inductive effect rather than coordination adjustment. In the Ru K-edge XANES spectra, the absorption edge of Ir_0.5_Ru_0.5_O_*x*_ is slightly higher than those of RuO_2_ and commercial RuO_2_, indicating a higher valence state of Ru in Ir_0.5_Ru_0.5_O_*x*_. Additionally, the Ru K-edge FT-EXAFS spectra show that the Ru–O distance in Ir_0.5_Ru_0.5_O_*x*_ is shorter than RuO_2_, revealing a strengthened Ru–O interaction in Ir_0.5_Ru_0.5_O_*x*_. The trend in the Ru coordination structure aligns with the valence state, likely due to interactions with Ir in the RuO_2_ lattice. Ru atoms in higher valence states are generally believed to be more active for acidic OER catalysis.^587^

### 8.6 X-ray Photoelectron Spectroscopy (XPS)

XPS is a powerful X-ray-based method, known for its surface sensitivity (0–10 nm) and ability to provide both elemental and chemical state information of materials. Survey scans are used to identify the elements present on the sample’s surface, offering an initial assessment of their relative amounts. Survey scans also serve as an effective method for identifying unexpected or contaminant elements. One significant advantage of XPS over other techniques is its ability to determine the chemical environment of atoms in a sample. This environment, which includes factors like nearest neighbors and the element’s oxidation state, influences the binding energy of both photoelectron and Auger peaks. The metal’s oxidation state also affects the binding energy for most transition metals.^592^

XPS is primarily used in PEMWE studies to analyze catalyst properties. In a study by Martin et al.,^593^ IrO_2_(110) film on Ir(100) surface was examined using XPS at two excitation energies: 720 eV, which provides bulk electronic insights (Figure 32a1), and 250 eV, which enhances surface sensitivity (Figure 32a2). The Ir 4f spectra from the clean Ir(100) substrate revealed an Ir 4f_7/2_ peak at 60.8 eV, corresponding to bulk Ir atoms, alongside a weaker component near 60.3 eV, attributed to surface Ir atoms. As expected, the surface metal signal was more pronounced at lower electron kinetic energy. After oxidizing the Ir(100) surface, the dominant Ir 4f_7/2_ peak shifted to 61.7 eV, indicating oxidation and aligning well with reported IrO_2_ binding energies. The IrO_2_(110) film analyzed at higher electron kinetic energy exhibited a bulk-related Ir 4f component near 60.8 eV, reinforcing its structural integrity.^593^ Freakly et al.^594^ revisited the XPS spectra of Ir, IrO_2_, and IrCl_3_, refining spectral interpretations and improving binding energy assignments. A critical aspect of the study is the curve-fitting of both Ir 4f and O 1s regions, which is essential for accurately distinguishing oxidation states and chemical environments. Fitting Ir spectra is particularly challenging due to the presence of multiple oxidation states and the asymmetric nature of the Ir 4f peaks, which requires careful deconvolution. The study addresses these complexities by optimizing peak shapes and accounting for screened and unscreened final states. Similarly, fitting the O 1s region is difficult because oxygen species can exist in various bonding environments, including lattice oxygen, hydroxyl groups, and adsorbed species. The authors demonstrated how hydration influences peak positions, complicating spectral interpretation. These refinements are crucial for electrocatalysis research, where precise characterization of Ir oxidation states and oxygen species is needed for understanding catalyst stability, reaction mechanisms, and degradation pathways. The study’s approach enhances reproducibility and provides a more reliable framework for analyzing Ir-based catalysts.^594^ The Ir and O fitting parameters established in this study are widely used by other researchers.

**Figure 32:**
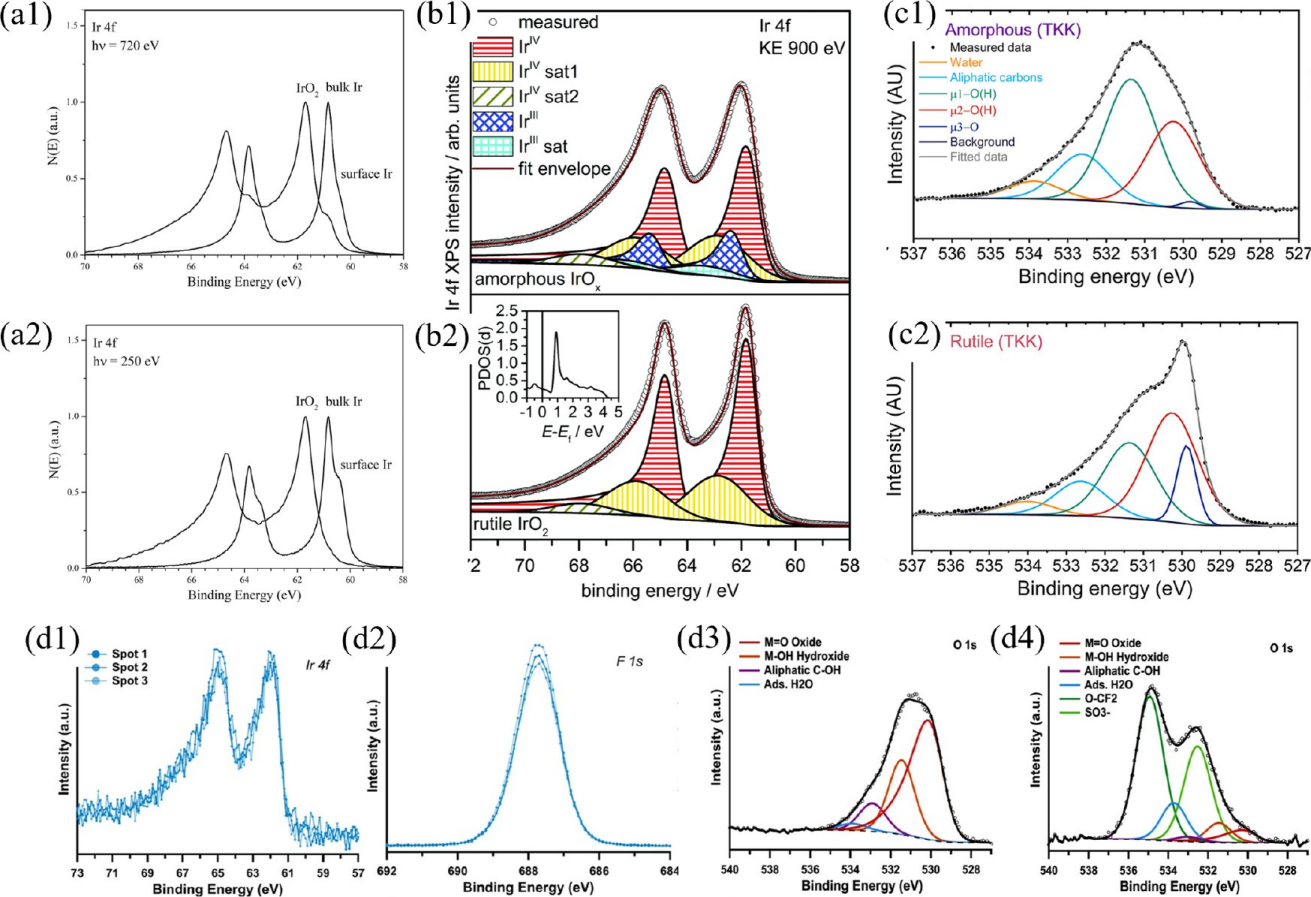
Normalized XPS Ir 4f spectra acquired from Ir(100) before and after oxidation of the surface (a1) 720 eV and (a2) 250 eV. Adapted from ref 593. Copyright 2020 American Chemical Society. Theory-based Ir fit models for (b1) amorphous IrO_*x*_ and (b2) rutile IrO_2_. Adapted from ref 590. Copyright 2016 The Authors under CC BY 3.0 Unported License (https://creativecommons.org/licenses/by/3.0/). O 1s XPS spectra with fitted contributions of oxygen and species of (c1) amorphous TKK and (c2) rutile TKK. Adapted from ref 566. Copyright 2024 The Authors under CC BY-NC-ND 4.0 International License (https://creativecommons.org/licenses/by-nc-nd/4.0/). High-resolution spectra of the spray coated CL 0.2 mg_Ir_ cm^–2^ of (d1) Ir 4f and (d2) F 1s. O 1s curve-fitting for (d3) bare IrO_2_ catalyst and (d4) 0.2 mg_Ir_ cm^–2^ spray coated CL. Adapted from ref 595. Copyright 2025 The Authors under CC BY-NC-ND 4.0 International License (https://creativecommons.org/licenses/by-nc-nd/4.0/).

Pfeifer et al.^590^ used a modeling approach to analyze Ir 4f spectra. Figure 32b1,b2 presents an example of Ir 4f peak fits derived from model studies for amorphous and rutile catalysts. Density functional theory (DFT) calculations were employed to investigate the presence of shakeup satellites in the Ir 4f spectrum of rutile IrO_2_ (Figure 32b2). The analysis predicted that the most prominent shakeup satellite would appear 1 eV above the main Ir 4f peak, with a weaker satellite positioned 3 eV higher. Using this shakeup model, the Ir^4+^ atoms in rutile IrO_2_ were fitted with a DS function peaking at 61.8 eV, accompanied by a Gaussian satellite at +1.0 eV. To further refine the fit, a small additional component at +3.1 eV relative to the Ir 4f_5/2_ line was introduced, accounting for shakeup effects from localized non-bonding states. Applying these theoretical insights, a fitting model for amorphous IrO_*x*_ was developed (Figure 32b1). The Ir 4f spectrum was deconvoluted into contributions from Ir^4+^ and Ir^3+^ species, each with its corresponding satellite structures. To accommodate the expected structural disorder in the amorphous phase, an increased full width at half-maximum (fwhm) for Ir^4+^ was used, reflecting the less regular local atomic environment compared to the well-ordered crystalline rutile structure.^590^

High-resolution O 1s spectra reveal distinct differences among samples with rutile, amorphous, and metallic phases. Low-binding-energy species are observed in samples with pure Ir rutile phases, while other samples exhibit a broader fwhm, indicating structural variations. Roiron et al.^566^ developed a curve-fitting model informed by oxygen species identified through O K-edge absorption and DFT calculations. The model incorporates signals from adsorbed water and oxygen associated with aliphatic carbon, validated by corresponding C 1s spectra (Figure 32c1,c2). The μ_3_-O component, associated with the non-defective oxide structure, appears at the lowest binding energy. While higher binding energy species correspond to defective oxide structures, either protonated or non-protonated (μ_1_-O­(H) and μ_2_-O­(H)). The developed model was applied to the O 1s spectra of several unsupported amorphous and rutile IrO_*x*_ catalysts; representative data from TKK amorphous and rutile IrO_*x*_ catalyst are shown in Figure 32c1,c2. As anticipated, oxygen atoms associated with water and aliphatic moieties exhibited comparable contributions across all catalysts. However, the μ_3_-O peak was significantly present only in the rutile catalyst, confirming its attribution to bulk rutile oxygen (Figure 32c2). The presence of μ_1_ and μ_2_-O features in rutile catalysts indicates the presence of surface oxide layer defects, even within the ordered crystalline phase. Interestingly, the ratio of these defects is inverted for amorphous and rutile catalysts, where amorphous IrO_*x*_ predominantly exhibits μ_1_-type defects (Figure 32c1), while the rutile phase favors μ_2_-type defects (Figure 32c2). Although the direct correlation between defect ratio and OER catalytic activity is not fully established in this study, it enables a more nuanced distinction between amorphous and rutile IrO_*x*_ surfaces and provides a deeper understanding for future studies of in-situ surface transformations, where surface modifications are expected to occur irreversibly during activation and reversibly at oxidizing potentials relevant to OER.^566^ Due to the complexity of the spectra and the peak overlaps in catalyst powders, peak fitting still presents significant challenges. Incorrect fitting can lead to misinterpretations of chemical states and surface compositions. The findings from these studies will contribute to the development of more precise analytical approaches for studying oxidation states, adsorption dynamics, and reaction mechanisms relevant to catalytic applications. Additional resources, such as the PHI handbook and XPS reference pages, provide comprehensive data sets for peak fitting and spectral analysis, further supporting accurate characterization.^596,597^

While XPS presents challenges for catalyst studies, analyzing CL layers introduces additional complexity to previously established fits, particularly in deconvolution of species from the catalyst and ionomer. Foster et al.^595^ investigated the homogeneity of IrO_*x*_ CLs produced by several methods, including spray coating (SP) and gravure coating (GV) at two different loadings of 0.2 and 0.4 mg_Ir_ cm^–2^. Figure 32d1–d3 shows data acquired from the SP CCMs with a 0.2 mg_Ir_ cm^–2^ loading, revealing that across three separate areas of analysis, the Ir 4f and F 1s exhibit strong consistency in peak magnitude, peak position, and overall shape. This spectral uniformity indicates that the SP technique produces surfaces with reasonably consistent spatial composition. Additionally, the reproducibility of spectra from different regions confirms that core-level signals can be summed up to enhance signal-to-noise without introducing artificial artifacts to the data. It is important to note that this study employed a more simplified fit for catalyst components compared to Roiron’s^566^ approach, primarily due to its focus on deconvoluting ionomer components in CLs made with the same catalyst (AA) and the dominant contribution of ionomer species in the CL spectrum. Specifically, the O 1s spectrum from the AA catalyst powder is fitted with contributions from M=O and M–OH, as established by Freakley et al.,^594^ along with aliphatic C–OH and adsorbed H_2_O (Figure 32d3), whereas the O 1s spectrum from the CL is fitted with two additional components: the O-CF_2_ component and the SO_3_^–^ component (Figure 32d4). It is clear that the majority of the O 1s spectrum is dominated by O-CF_2_ and SO_3_- species, with a lower contribution from M=O and M–OH.^595^

In addition to the investigation of CLs and catalysts, XPS is commonly used to analyze other components such as PTLs and BPPs. Bystron et al.^570^ examined the surface composition of pristine and etched Ti felt PTLs. The pristine Ti 2p spectrum was dominated by the Ti^4+^ 2*p*_3/2_ peak at 458.6 eV, with lower oxidation state contributions barely visible, indicating the prevalence of TiO_2_ in the passive layer. However, etching significantly altered the surface composition, revealing distinct spectral lines for Ti^3+^ (456.2 eV) and Ti^0^/Ti^2+^ (454.3 eV). Given the uncertainty in distinguishing oxidation states within this range of 0 to + II, these species were collectively assigned to Ti^0^/Ti^2+^. Simultaneously, the intensity of the Ti^4+^ peak decreased considerably, further confirming a substantial transformation in surface chemistry.^570^

### 8.7 Scanning Electron Microscopy – Energy Dispersive X-ray Spectroscopy (SEM-EDS)

SEM-EDS is a powerful analytical technique that enables imaging and elemental composition analysis across a wide range of magnifications. The main interactions include the emission of secondary electrons (SEs) through inelastic scattering and backscattered electrons (BSEs) generated through elastic scattering.^598^ SE images are generated from the sample surface’s top 5–50 nm, whereas BSE images originate from deeper regions, up to 450 nm into the sample. SE imaging is highly sensitive to surface details, providing information about the sample’s morphology. In contrast, BSE imaging is sensitive to differences in atomic number (Z-contrast), with higher atomic number elements appearing brighter in BSE images, allowing for compositional contrast.^599^ An EDS detector is typically an integrated component of the SEM instruments allowing for quantitative elemental composition analysis. EDS can analyze up to 3 μm into the sample.^600^ EDS generates both spectra and elemental maps by detecting characteristic X-ray emissions, identifying elements through spectral peaks while mapping their distribution.^601^ SEM-EDS can characterize areas of interest of individual as well as integrated components, making it an invaluable tool for characterizing every component in a PEMWE cell. Both top-down and cross-sectional analysis are commonly used and are often combined for a more comprehensive analysis, as shown in Figure 33f–h. This analysis is often conducted to study the influence of fabrication parameters, and if conducted before and after cell use, to assess potential degradation mechanisms. SEM-EDS analysis allows for comparing differences in thickness, changes in the CL structure, interface with PEM or PTL, and provides details about the distribution of various elements representing different components.^194,602^

**Figure 33:**
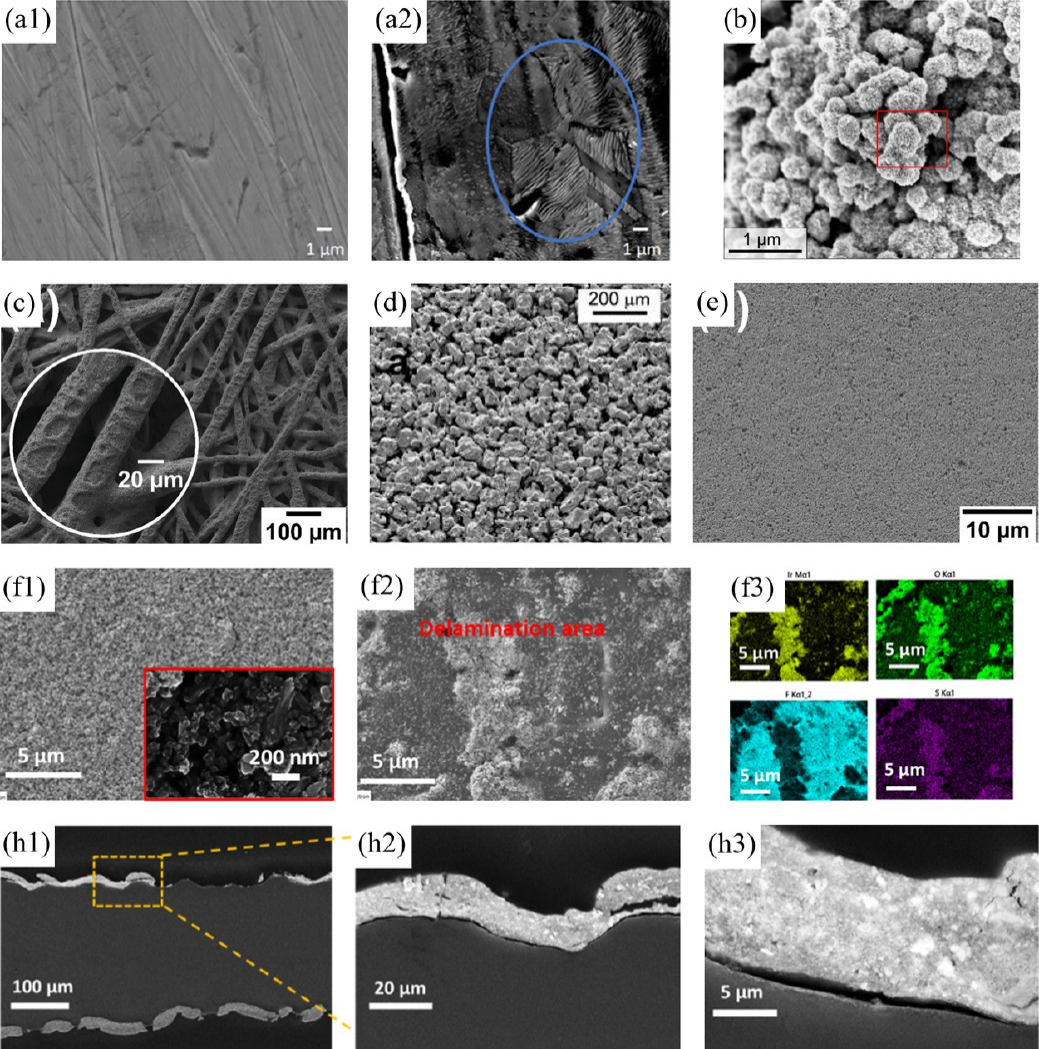
Top-down SEM images of: ss-BPP (a1) pristine and (a2) aged. Adapted from ref 328. Copyright 2022 The Authors under CC BY 4.0 International License (https://creativecommons.org/licenses/by/4.0/). (b) Pristine IrO_2_/TiO_2_ catalyst powder. Adapted from ref 167. Copyright 2023 The Authors under CC BY 4.0 International License (https://creativecommons.org/licenses/by/4.0/). (c) felt PTE, (d) sintered PTE, (e) CCM. Adapted from refs 130,604 . Copyright 2023 The Authors under CC BY 4.0 International License (https://creativecommons.org/licenses/by/4.0/). (f1) pristine MEA, (f2) aged MEA, (f3) EDS maps of aged MEA Ir, O, F, S and cross-sectional SEM images of (h1–3) aged MEA. Adapted from ref 24. Copyright 2023 The Authors under CC BY 4.0 International License (https://creativecommons.org/licenses/by/4.0/).

Figure 33a1,a2 presents surface images of SS BPPs, depicting pristine and aged samples, respectively. SEM-EDS analysis was conducted by Stiber et al.^328^ on both coated and uncoated BPPs, before and after testing, to understand their degradation and correlate structural changes with performance metrics.^328^ The PTL can also be analyzed using SEM-EDS to assess surface morphology changes and, in the case of Pt-coated PTLs, to confirm uniform Pt deposition.^603^ Building on this analysis, the Ti PTL can undergo SEM-EDS characterization to assess surface modifications resulting from various post-treatments such as acid etching. This treatment introduced Ti hydride in the subsurface, mitigating excessive passivation and lowering resistance. By enhancing conductivity, the etched Ti PTL supports long-term electrolyzer performance and offers a cost-effective alternative to precious metal coatings.^570^

SEM-EDS is also often used to characterize the catalyst powders to determine elemental composition, morphology, and particle size, with the goal to confirm catalyst synthesis (Figure 33b).^167^ Conversely, PEMs are less frequently analyzed using SEM-EDS due to challenges such as charging and melting under SEM conditions. Therefore, they are more commonly examined post-cell operation after integration with CLs to assess changes in their contact and interaction.^605^ SEM-EDS is also used to study CLs upon their integration with PEMs or PTLs, producing CCMs (Figure 33e) and PTEs, respectively. The most common substrates for PTEs are fiber-based and sintered PTLs (Figure 33c,d, respectively). The top-down SEM images reveal distinct morphologies, demonstrating the most uniform catalyst distribution with CCMs, and highlighting greater challenges for uniform coating with PTLs due to their porosity.^130,604^ Another study of SEM-EDS cross-sectional applicability to help optimize fabrication methods by Ferner et al.^57^ investigated composite anodes made with TKK and AA IrO_*x*_ and a Pt black conductive additive that was used to reduce Ir usage while maintaining performance. TKK consists of large, solid particle structures, while AA is characterized by smaller, more uniformly sized particles. Although both the AA IrO_*x*_ catalyst and its composite contain ∼0.1 mg cm^–2^ IrO_*x*_, the AA IrO_*x*_ catalyst forms a significantly thinner CL. This study demonstrates that incorporating Pt black helps sustain CL thickness, enabling reduced Ir usage without compromising structural integrity.^57^

Figure 33f1,f2 presents SEM images of the pristine and aged MEA, respectively. At low magnification, the images reveal variations in CL structure. The pristine MEA exhibits a dense, compact CL with a high degree of homogeneity, while the aged MEA displays the CL being detached in some areas and instead adheres to the adjacent PTL. The delamination of the CL was confirmed via EDS mapping (Figure 33f3), where dark regions in the Ir map correspond to PTL fiber imprints, highlighting areas where the CL adhered to the PTL, ultimately leading to CL delamination. Meanwhile, the brighter regions in both the F and S maps correspond to exposed membrane surfaces. Cross-sectional analysis of the aged MEA confirms CL detachment, with some regions showing complete separation from the membrane (Figure 33h1). In other areas, the CL remains partially attached but exhibits curved deformation (Figure 33h2,h3). Sections that remain connected display surface curvature and localized detachment, alongside the formation of voids and cracks, likely resulting from Ir dissolution.^24^

### 8.8 X-ray Computed Tomography (XCT)

XCT is a non-destructive imaging technique that reconstructs 3D representations of a material’s internal structure by capturing X-ray projections from multiple angles. It has become an essential tool for PEMWE characterization, enabling structural analysis across various components. XCT is highly complementary to SEM and TEM, as it provides high-resolution 3D imaging of internal structures, particularly for PTLs, CLs, and MEAs. It facilitates the assessment of PSD, tortuosity, and thickness variation, while also supporting water management studies to optimize mass transport and mitigate flooding. Its ability to perform time-resolved, operando studies makes it uniquely suited for monitoring catalyst performance and material evolution in real electrochemical environments. Early XCT studies focused on morphology and structure, helping researchers to understand PSD, tortuosity, and porosity variations. However, the high X-ray attenuation of Ti PTLs posed challenges, introducing image artifacts that required correction to ensure accurate interpretation. As XCT technology advanced, its applications expanded beyond PTL characterization. Micro-XCT, which operates at micron-scale resolution, emerged as a valuable tool for analyzing PTL porosity in both laboratory-based and synchrotron-based experiments. Later, Nano-XCT, capable of resolving submicron features down to tens of nanometers, became more widely used for CL characterization, offering deeper insights into catalyst dispersion and degradation mechanisms. More recently, operando XCT studies have allowed researchers to capture time-resolved electrochemical processes, revealing dynamic interactions between dissolution and redeposition mechanisms. These studies require special cell designs, ensuring that the X-ray beam path remains sufficiently transparent for accurate analysis. Additionally, 4D imaging approaches now enable scientists to correlate material structure with electrochemical performance, leading to optimized electrode designs. It allows for 3D visualization inside electrolyzers, making it possible to investigate oxygen transport mechanisms and refine PTL designs.^110,606^

SEM and XCT offer highly complementary insights, as demonstrated by Weber et al.^607^ in the investigation of MPL and PTL structures. SEM provided high-resolution surface imaging for detailed microparticle morphology analysis, as seen in the top images of Figure 34a1–a3, which highlight the MPL with a protective coating, an MPE, and a felt PTE (left to right). XCT, meanwhile, enabled 3D volumetric assessment of the MPL and PTL structures and their coatings, revealing connectivity and internal distribution, illustrated in the XCT-based surface renderings at the bottom of Figure 34a1–a3. The combined use of SEM and XCT exposes CL distribution and penetration of the catalyst into Ti substrate voids, demonstrating how SEM captures localized surface features while XCT uncovers bulk structural relationships. This dual approach enhances the understanding of material structure, particularly in electrochemical systems where surface and bulk properties jointly influence performance.^607^ Zielke et al.^608^ conducted an XCT study on eight fiber PTL materials, comparing thermal conductivity, porosity, water permeability, and electrical conductivity. Their findings advanced PSD analysis in sintered and fiber PTLs while highlighting challenges in linking structure to performance. Despite these complexities, the study identified two key correlations: an exponential relationship between porosity and two-phase thermal conductivity, and a strong link between electrical conductivity and water permeability.^608^ These insights provide a basis for future work, aiding in the development of models that reduce parameter variability when linking PTL morphological characteristics to performance metrics and experimental testing.

**Figure 34:**
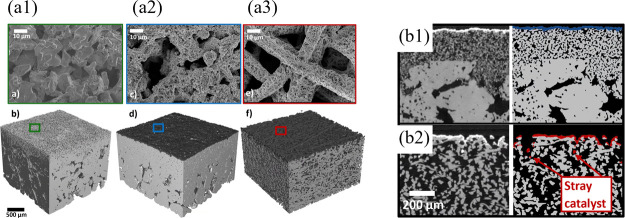
Top-down SEM images (top) and XTM surface renderings (bottom) of (a1) MPL + support layer, (a2) MPE with 2.5 mg_Ir_ cm^–2^ loading, and (a3) Ti-felt (Bekaert) with 2.5 mg_Ir_ cm^–2^ loading. Cross-section XCT slices, as gray scale (left) and segmented (right) images of (b1) an MPE and (b2) a PTE (based on Ti-felt) with 2.5 mg_Ir_ cm^–2^ loading, where the pores are black and the CL is in blue and red. Adapted from ref 607. Copyright 2023 The Authors under CC BY 4.0 International License (https://creativecommons.org/licenses/by/4.0/).

Figure 34b1,b2 presents cross-sectional XCT slices which show the distribution of the CL at the PTL/CL interface. In grayscale images, the CL appears as a white line, while in segmented volume renderings, it is depicted in blue for the MPE and in red for the PTE. The MPE exhibited a homogeneous catalyst distribution, forming a continuous layer near the surface. In contrast, in the PTE, a significant fraction of the CL extended into large voids within the PTL, resulting in isolated catalyst regions distant from the PTL surface. Black regions represent a continuous catalyst network, whereas red and blue regions indicate isolated CL areas. A comparative analysis revealed that in the PTE, more than 5% of the CL remains isolated, while in the MPE, only 0.3% is separated from the continuous network. Conversely, in the PTE, a significant portion of the CL penetrates deep into the PTL bulk. Weber et al.^607^ estimated that approximately 46% of the CL in the PTE remains inaccessible and therefore unutilized. In comparison, this effect is significantly reduced in the MPE, where only ∼18% of the CL is unavailable, as the majority remains close to the MPL surface.^607^ XCT-based imaging has proven highly effective in enhancing PEMWE performance analysis, particularly in optimizing PTL structure and CL morphology. Studies applying XCT to PTEs have primarily reported results for uncoated PTL baselines, likely due to the challenges associated with differentiating Ir and Pt using this technique. Additionally, the high X-ray attenuation of Ti PTLs introduces image artifacts, necessitating the use of advanced correction methods to ensure accurate interpretation.

### 8.9 Atomic Force Microscopy (AFM)

AFM is a less common, but also powerful tool for investigating surface structures and properties of various materials used in PEMWEs, including PEM, CCM, and BPP, providing information complementary to SEM and TEM. AFM allows for the evaluation of surface morphology and other surface properties such as stiffness, adhesiveness, viscosity, chemical structure, hydrophobicity/hydrophilicity, conductivity, and surface potential with nanometer resolution. One of its key advantages is its ability to operate under diverse environmental conditions without needing a vacuum or conductive coating. Interaction forces between the probe and the surface can also be measured in situ, providing comprehensive data during surface scanning.^609^ Obtaining high-resolution surface observations and interaction force measurements with AFM is relatively straightforward, and AFM measurements are typically non-destructive.

AFM is used to improve understanding of the performance, durability, and efficiency of CCMs before and after operation.^93,328,610,611^ A study by Torrero et al.^93^ analyzed CCMs with AFM to investigate the catalyst coverage on the membrane. Specifically, conductive and nanomechanical modes were used to investigate CCMs at three operation stages: beginning of test (BOT), short time test (SOT), and end of test (EOT).^93^ High-resolution AFM images revealed the 3D topography of the CL, with smoother surfaces at EOT due to increased ionomer coverage. PeakForce-TUNA mode measurements showed significant ionomer coverage on particles, with electron flux assessed by applying voltage between the CL and a PtIr-coated AFM tip. BOT anodes exhibited low electronic conductivity, indicating high ionomer coverage. After activation, SOT anodes showed a 10-fold increase in conductive area, aligning with enhanced OER activity. At EOT, the conductive area dropped to just 0.1% after 1,000 h, while electrochemical activity remained high. Torrero et al.^93^ showed that performance relies on the interaction between ionic and electronic conductivity and catalyst accessibility, rather than in-plane or cross-particle conductivity.

A study by Liu et al.^610^ used AFM to analyze the anode CL of pristine and aged MEAs, assessing changes in relative conductive area and surface roughness. In addition to electronic conductivity measurements, simultaneous height and deformation recordings were conducted. Figure 35 presents the height, current, and deformation data for both MEAs. Height measurements revealed a clear increase in surface roughness following long-term operation (Figure 35a,d), with values rising from 21.2 nm for the pristine anode to 32.8 nm for the aged MEA. This roughness increase could stem from mechanical interactions with the PTL, ionomer degradation, or ionomer redistribution. To further investigate ionomer changes over time, the electronic conductive area and surface deformation were examined. The study found that CL surface conductivity was affected by ionomer loss, with AFM imaging requiring high humidity or liquid water and an increased bias to accurately measure ionic conductivity. A rise in electronic conductive area was interpreted as evidence of ionomer degradation or rearrangement. Additionally, ionomer deformation provided contrast in AFM measurements; lower deformation areas indicated ionomer loss or structural rearrangement postoperation. AFM images in Figure 35b,e show that IrO_*x*_ nanoparticles remained homogeneously distributed in conductive regions, whereas non-conductive areas were primarily associated with the ionomer. The pristine CL exhibited a 41% conductive area, which increased to 83% after 4,000 h of operation, signaling ionomer depletion on the CL surface. Possible structural modifications include ionomer flow under applied potential and high temperature, as well as catalyst redeposition onto the ionomer. This ionomer rearrangement was evident in Figure 35f, where deformation measurements indicated a reduced highly deformable area for the aged MEA. In summary, AFM findings demonstrate that cell performance decrease was influenced by ionomer degradation in the MEA. Moreover, Figure 35b,e confirms an increase in electronic current for the aged MEA, reinforcing the observed material transformations.^610^

**Figure 35:**
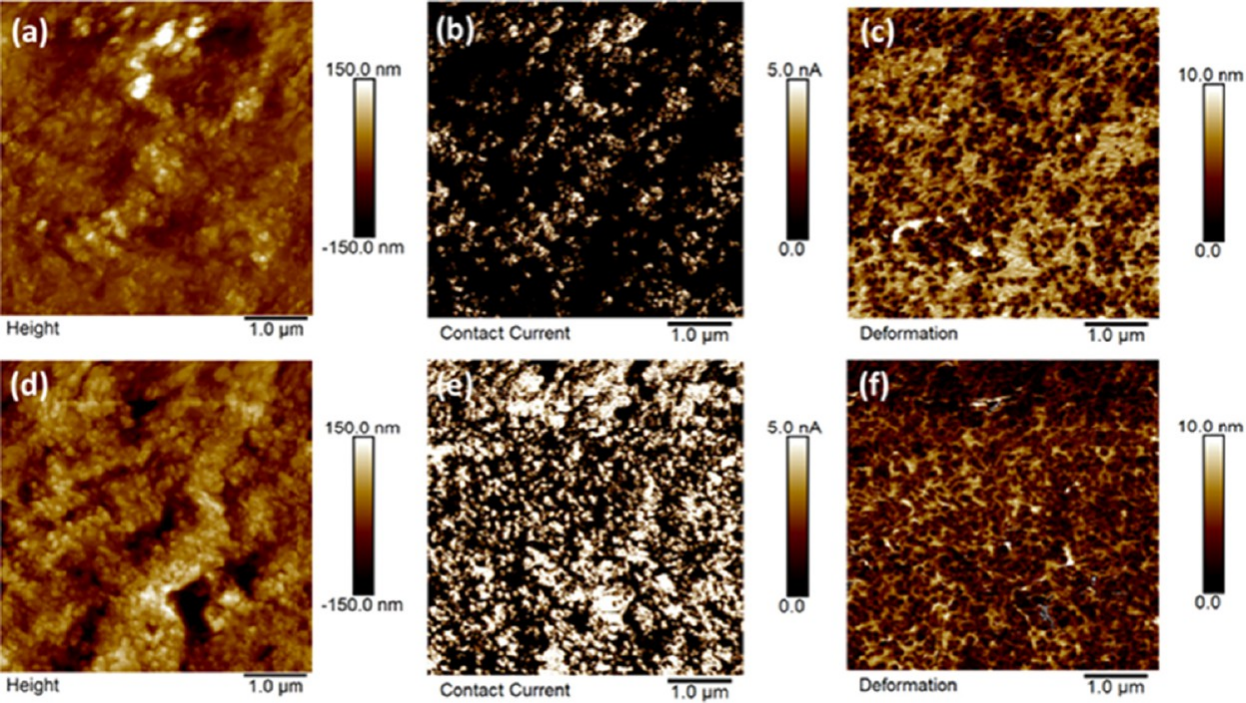
AFM measurements of pristine (top) and aged (bottom) MEAs: (a and d) height measurement, (b and e) conductivity measurement, (c and f) deformation measurement. Adapted from ref 610. Copyright 2023 The Authors under CC BY 4.0 International License (https://creativecommons.org/licenses/by/4.0/).

Additionally, AFM measurements can be carried out to investigate the structure and conductivity of BPP. A study by Stiber et al.^328^ for the SS substrate in pristine and aged samples revealed scratches and cracks. These cracks and microstructural defects can exhibit elevated hydrogen concentrations due to localized metal displacement, which serves as an initiation point for crack formation. Furthermore, Stiber et al.^328^ observed a substantial reduction in conductivity following operation. To investigate this, substrate areas were analyzed using conductive tapping AFM mode. Comparisons revealed that the conductive area at a bias voltage of 3 V for BPP decreased significantly, from 44 to 3% after operation.^328^

### 8.10 Transmission Electron Microscopy (TEM)

TEM generates images from ultra-thin samples (less than 200 nm) by directing a high-energy electron beam (80–300 keV) through a vacuum column (10^–5^ to 10^–7^ mbar). Electromagnetic lenses, formed by wire coils producing deflecting forces, shape the beam as it passes through the sample, interacting with additional lenses before reaching detectors and cameras. TEM enables high-resolution imaging (subnanometer scale), high-magnification imaging (up to 1,000,000×), allowing visualization of atomic columns in crystalline samples and providing structural and elemental insights. TEM operates in two primary modes: TEM mode, which uses a parallel beam for broad imaging, and scanning transmission electron microscopy (STEM) mode, which employs a localized probe for detailed atomic-scale imaging. High-angle annular dark field (HAADF)-STEM further enhances resolution by leveraging high-angle electron scattering. In bright-field (BF) imaging, contrast is generated as lighter elements appear dark, while heavier elements create bright regions due to differences in electron scattering. Additionally, diffraction patterns can be observed by inserting an objective aperture, which filters transmitted electrons to improve contrast.^612–614^ Furthermore, TEM capabilities are significantly enhanced by integrating EDS and electron energy-loss spectroscopy (EELS).^615^ EDS provides elemental composition data via characteristic X-ray emissions, while EELS enables analysis of excitations and ionizations within the sample. EELS facilitates the investigation of core–shell transitions, plasmon resonance, valence-to-conduction band transitions in semiconductors, and vibrational modes (phonons), broadening the scope of material characterization.^616^

TEM characterization can be applied to various components of the PEMWE, with the most common being the catalyst, CL, and the MEA, before and after operation. Due to the need for ultra-thin samples the sample preparation for TEM is more demanding than for SEM. This often involves microtoming or focused ion beam (FIB) milling to achieve the necessary thickness. Additionally, TEM samples may need support grids for stability. Cross sections of CCMs are most commonly prepared by microtoming, while analysis of PTLs and PTEs requires preparation of FIB lift-outs with either a plasma FIB or a traditional FIB.^617,618^ Despite the complexity of sample preparation, TEM provides a more comprehensive nanoscale analysis compared to other characterization techniques. While SEM excels in surface morphology imaging, TEM provides detailed visualization of internal microstructures, enabling a deeper understanding of structural changes at the atomic level. Unlike XPS and XAS, which focus on average chemical composition and oxidation states, TEM-EDS captures elemental composition and morphology of individual particles, along with their dispersion; and nanoscale transformations, making it a crucial tool for assessing degradation mechanisms in PEMWE.^602^

TEM-EDS is crucial for the analysis of the PTL and its protective coatings. Liu et al.^610^ reported a TEM-EDS map of the interfacial region of an Ir-coated PTL after ≈4,000 h of operation under real PEMWE conditions (Figure 36a1). EDS elemental mapping and line-scanning profiles from the FIB cross-section confirm that the Ti fiber is uniformly coated with Ir. Figure 36a1,a2 further reveals a 7 nm-thick native TiO_*x*_ layer (pink) beneath the added Ir layer (green), which retained its 20 nm thickness post-testing, indicating that the Ir layer remained intact without significant degradation. However, *a* < 10 nm IrO_*x*_ layer formed on top of the Ir coating, suggesting an initial oxidation process occurred while preventing further oxidation of metallic Ir within the bulk. Most notably, this thin Ir layer inhibited the growth of the TiO_*x*_ passivation layer, preventing a significant increase in cell resistance, thereby enhancing long-term electrochemical performance.^610^ Details of catalyst particle morphology and structure, including their size, shape, and distribution, are typically captured with HR-STEM and HAADF-STEM. A study by Cheng et al.^619^ highlighted notable structural differences between IrO_*x*_·*n*H_2_O and IrO_2_ catalyst powders (Figure 36b1–b4). HR-STEM images of IrO_*x*_·*n*H_2_O exhibit short, disordered lattice stripes, indicative of dense, aggregated particle formations made up of short, ordered nanoclusters (Figure 36b1). The IrO_2_ powder demonstrates a well-defined crystal lattice, showcasing its high crystallinity (Figure 36b2). HAADF-STEM provides further insights, revealing that IrO_*x*_·*n*H_2_O had a more continuous structure with fewer voids, whereas a looser, more porous arrangement characterized IrO_2_ (Figure 36b3,b4).^619^

**Figure 36:**
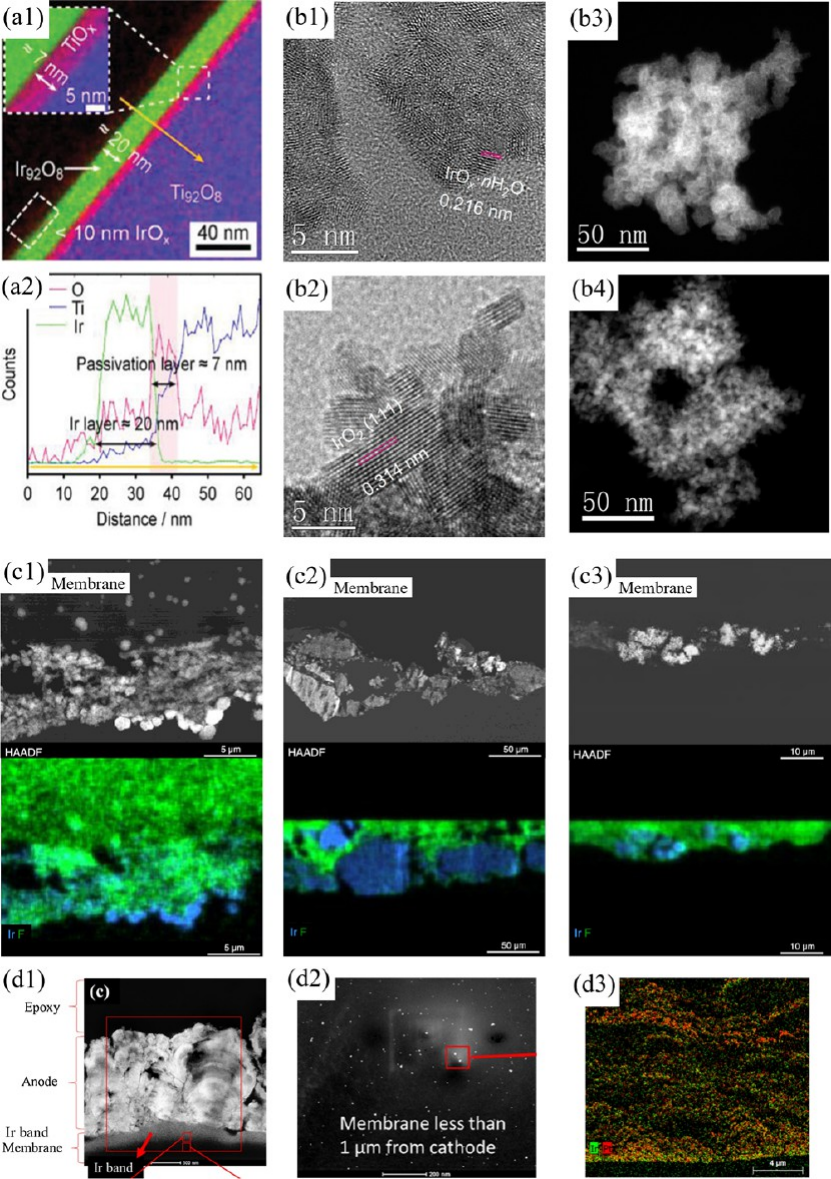
FIB cross-section of the Ir-coated PTL after 4,000 h of operation (a1) EDS composition analysis, (a2) Line-scanning profile. Adapted from ref 610. Copyright 2021 The Authors under CC BY 4.0 International License (https://creativecommons.org/licenses/by/4.0/). High-resolution TEM images of (b1) IrO_*x*_·*n*H_2_O and (b2) IrO_2_. HAADF-STEM images of (b3) IrO_*x*_·*n*H_2_O and (b4) IrO_2_. Adapted from ref 619. Copyright 2024 The Authors under CC BY 4.0 International License (https://creativecommons.org/licenses/by/4.0/). HAADF-STEM and EDS spectrum images (Ir, F) of cross-sectioned anode CLs after AST from MEAs with (c1) AA Ir, (c2) TKK Ir, (c3) JM Ir. Adapted from ref 591. Copyright 2024 The Authors under CC BY 4.0 International License (https://creativecommons.org/licenses/by/4.0/). HAADF-STEM EDS images of degraded MEA (d1) anode CL, (d2) membrane adjacent to the cathode, (d3) EDS map of the cathode. Adapted from ref 194 with permissions from Elsevier B.V.

STEM-EDS imaging is invaluable for the investigation of CLs and provides important insights into the uniformity, porosity, distribution, thickness, and the interfaces with the PEM. A study by Zaccarine et al.^602^ used TEM to compare CLs made of Ir black and IrO_2_, revealing distinct morphological differences. Ir black formed larger, less dense agglomerates, while IrO_2_ exhibited smaller, more compact structures with a more uniform oxide distribution throughout the particles. These differences influence CL stability and degradation behavior under electrochemical conditions.^602^ Several studies analyzed cross sections of MEAs after durability testing to assess how degradation varied with different anode catalysts.^194,591,602,611^ A study by Alia et al.^591^ reported that CL made with the AA IrO_*x*_ showed Ir migration into the membrane, although significant amounts of the catalyst remained in the CL (Figure 36c1). In contrast, for TKK and JM Ir metal catalysts (Figure 36c2,c3), Ir migration was minimal to none. However, the ionomer appeared non-uniform, with Ir aggregates (blue) forming in areas with little to no fluorine (green) signal. HAADF images revealed denser catalyst patches, suggesting particle agglomeration. For TKK Ir (Figure 36c2), larger catalyst blocks formed, while the JM Ir CL (Figure 36c3) was significantly thinner after durability testing, likely indicating higher catalyst dissolution and migration.^591^ Another example from work by Yu et al.^194^ focused on degradation in both the anode and cathode within the MEA. The study examined Ir dissolution, migration, and redistribution, revealing Pt–Ir precipitate formation in the membrane and catalyst morphology evolution over time. STEM imaging shows the formation of an Ir band in the membrane adjacent to the anode CL, with a thickness of roughly 500 nm (Figure 36d1). This shows direct evidence of Ir dissolution during electrolysis at 1.8 A cm^–2^ (anode potential: 1.9–2.0 V), with high-magnification images and EDS mapping revealing small Ir particles within the band and density gradually decreasing toward the membrane. Some Ir ions continue to migrate through the membrane (Figure 36d2), eventually reaching the cathode, where they are deposited (Figure 36d3).^194^

Another degradation mechanism that is associated with the anode includes corrosion processes that are taking place at the interface between the Ti PTL and the protective Pt coating. If the Pt deposit is not conformal, then at high anodic potentials and low pH, corrosion processes take place preferentially at the interfaces between the Pt and Ti and the Pt deposit can detach from the PTL.^304^ An example of such Pt flakes that are detached from the Ti PTL and remain on the surface of the IrO_*x*_ CL has been observed by Zeng et al.^620^ with cross-sectional TEM images of the aged MEA. These dense Pt flakes can dissolve under high operating voltages, generating Pt ions that may migrate within the CL. These Pt ions can either deposit on the Ir catalyst or interact with the ionomer in the CL, leading to reduced catalytic activity.^620^

### 8.11 Time-of-Flight Secondary Ion Mass Spectrometry (ToF-SIMS)

In Tof-SIMS, a pulsed ion beam ejects molecules from the outermost layers, generating secondary ions for analysis which are accelerated through a flight tube, and then classified by their arrival time at the detector.^621^ This method provides high mass resolution and offers exceptional lateral resolution (50 nm) across large fields of view. Two primary modes exist: static and dynamic ToF-SIMS.^622^ Static ToF-SIMS uses a liquid metal ion gun (LMIG) as the primary ion source. This analysis is non-destructive, with a Ga gun suited for elemental analysis and an Au or Bi gun for molecular analysis. Dynamic ToF-SIMS, in contrast, employs a secondary sputter source for depth profiling, progressively removing material to reveal deeper layers. This destructive method utilizes O_2_, Cs, C_60_ or Ar-cluster beams, with C_60_ cluster ions optimizing polymer analysis and Ar-cluster beams for soft matter characterization.^623^ ToF-SIMS depth profiling enables high-resolution, 3D chemical mapping, uncovering surface contaminants, interfacial chemistry, and molecular variations. Consequently, ToF-SIMS excels in surface and interface analysis, making it invaluable for material characterization.^624^

ToF-SIMS has been used to investigate the surface properties and corrosion resistance of SS, critical for electrical conductivity and mechanical support in BPPs.^625^ A study by Liu et al.^610^ employed ToF-SIMS to investigate protective coatings on PTLs with an emphasis on coating identification and interface characterization between the PTL and its coating. Figure 37 presents ToF-SIMS depth profiles for uncoated, Ir-coated, and Pt-coated PTLs before and after 4,000 h of operation. The profiles show the counts of Ti-containing ions (from the Ti fibers), Ir-containing ions (from the Ir coating), Pt-containing ions (from the Pt coating), and Cs_2_O^+^, representing all oxides present in both the PTL and coating, against sputtering time. Metallic and oxidized Ti species were observed across pristine and aged samples, confirming the presence of TiO_*x*_ even in the pristine PTLs. After 4,000 h of operation, the uncoated PTL exhibited a thicker, rougher TiO_*x*_ layer, evidenced by a longer TiO^+^ (black) tail with a gentler slope (Figure 37d). This change was not observed in the Ir- and Pt-coated PTLs, which showed similar trends to each other, with metallic Ir and Pt species remaining present on the Ti fiber surfaces (Figure 37b,c). Notably, slight differences emerged in the interfacial oxide regions. The Ir-coated PTL exhibited a slight increase in oxide layer width (red), suggesting mild oxide growth (Figure 37e). Whereas the Pt-coated PTL showed minimal change between pristine and aged samples, indicating no additional oxide growth.^610^ This study confirms that ToF-SIMS effectively tracks TiO_*x*_, PtO_*x*_ and IrO_*x*_ interfacial layers and highlights the role of protective coatings.

**Figure 37:**
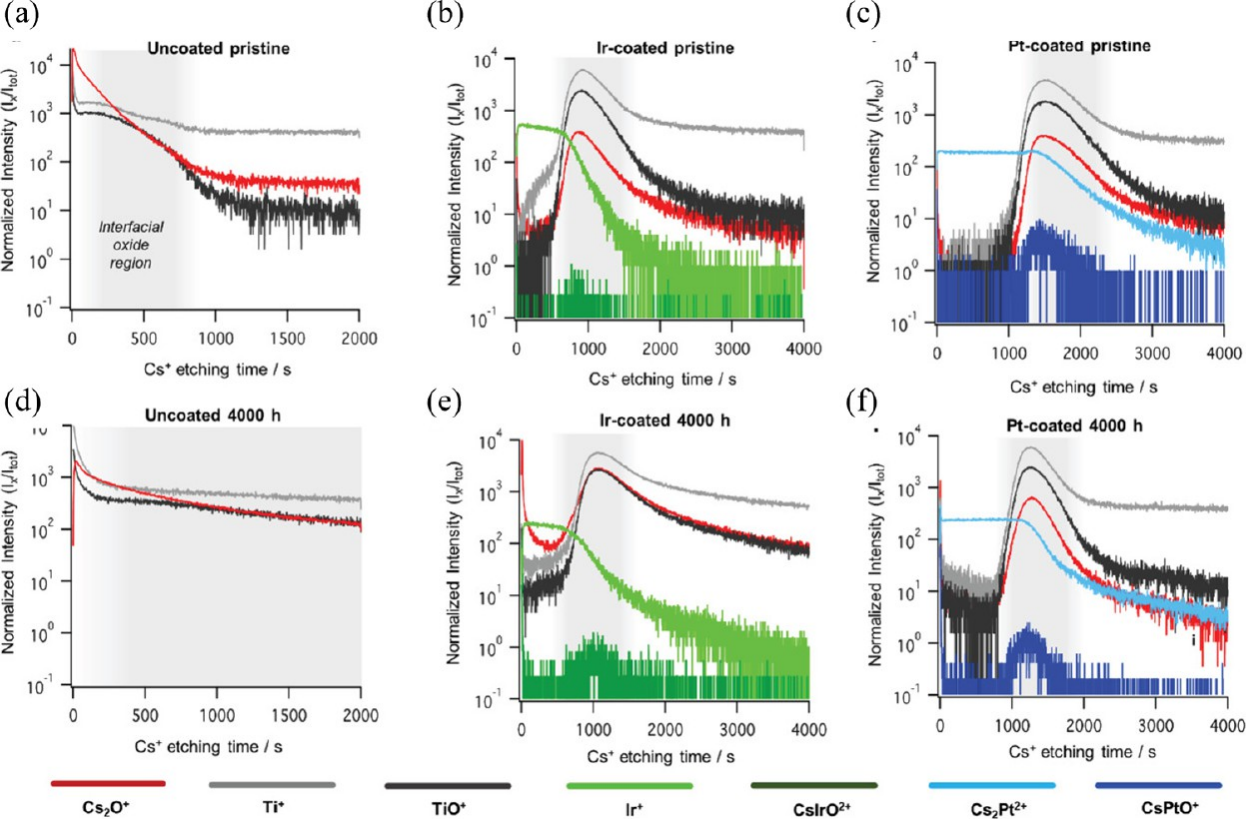
ToF-SIMS depth profiles of PTLs (a) pristine uncoated PTL, (b) pristine Ir-coated PTL, (c) pristine Pt-coated PTL, (d) uncoated PTL after 4,000 h, (e) Ir-coated PTL after 4,000 h, and (f) Pt-coated PTL after 4,000 h. Adapted from ref 610. Copyright 2021 The Authors under CC BY 4.0 International License (https://creativecommons.org/licenses/by/4.0/).

ToF-SIMS is widely recognized in materials science for its exceptional sensitivity and versatility. While preliminary studies on PEMWE components have yielded promising results, expanding its application to additional elements such as CLs is crucial. Given the morphological complexity of PEMWE samples, a deeper understanding of ToF-SIMS’s capabilities and limitations is essential. Further investigation is needed to fully assess its broader potential in this field.

## 9 Summary

The hydrogen economy will play a crucial role in the worldwide decarbonization, offering a versatile energy vector and feedstock for industrial and fine chemical production combined with renewable energies. In this context, PEMWEs emerge as an indispensable element for the electrochemical generation of high-purity hydrogen, which can be utilized in various applications, including PEMFCs, sustainable combustion, gas turbines, fertilizer production, and refinery processes. However, the widespread use of PEMWE technology still faces some hurdles, including limited performance and durability at a reduced cost. The efficient use of noble metals, materials engineering, and optimized design are crucial aspects for further advancing the technology and promoting its worldwide adoption. In this review, a comprehensive survey of the main accomplishments in the field, the current status of research activity, and opportunities and challenges for future development has been presented. The work addressed the study of the main cell components (CL, PEM, PTL, BPP, and gasket), along with the key input from multiscale, multipurpose modeling and characterization techniques. The main conclusions drawn from the different topics covered in this review are as follows:

### 9.1 Catalyst Layer

For PEMWE systems to be deployed at the GW scale, the most urgent objective of CL design is to minimize the total PGM loading, while not sacrificing high current densities and long-term durability. While minimal Pt loadings (∼0.05 mg_Pt_ cm^–2^) can be achieved at the cathode, high anode Ir loadings (>1 mg_Ir_ cm^–2^) are still required to overcome the harsh conditions and slow kinetics of the OER. Due to the high cost of Ir, improving the anode CL toward lower Ir loadings is a more prioritized challenge than the cathode. However, efforts to design the anode CL solely with a cost-optimization approach will often compromise the activity, stability, and/or durability. In recent years, the development of alternative catalyst materials and designs has shown promising progress toward improved performance at low Ir loadings. Namely, Ir-based alloys, supported Ir nanoparticles, and extended surface area or high aspect ratio morphologies are common alternatives to conventional ionomer-bound IrO_*x*_ nanoparticles. Yet, key areas for further performance improvement at the anode CL include increasing catalyst surface area, electrical conductivity, and interfacial contact with the PTL. Attention to high-quality fabrication of CLs is also critical. Modifying the CL ink dispersion, composition, and coating methods affects performance due to changes, such as variations in particle size and ionomer distribution. Continuous work to build a stronger understanding of how CL fabrication correlates to performance is crucial for eliminating overpotentials due to design and reducing specific degradation mechanisms. Another aspect that deserves further consideration is the multifaceted role of the ionomer in the CL. Ionomers are presented as a nanometer-thick film in the electrodes, acting as a proton conductor, a binder for the catalyst, and aiding in local water management. While decreasing the amount of ionomer is linked to poor kinetics from reduced proton transport pathways, increasing it can block the transport of species, requiring optimization of these functions by controlling the ionomer chemistry and coverage for improved catalyst utilization. One must note that the catalyst performance investigated in an aqueous-electrolyte environment using RDE measurements cannot always be representative of the MEA performance, due to the critical role of ionomer-catalyst interactions, which should be examined further in conjunction with ink design and CL fabrication strategies. As a final remark, it is worth noting that a better understanding of degradation mechanisms and efforts to standardize ASTs will be necessary to evaluate the long-term operation of new CL materials and designs.

### 9.2 Membrane

PEM water electrolysis membranes have historically been based on relatively thick (125–175 μm) PFSA ionomers. However, the industry is trending toward thinner membranes to meet efficiency and cost targets, requiring more highly engineered constructions. For these new membranes to meet the expected lifetime targets, a deeper understanding of their mechanical and chemical durability is required. To date, little is known about the mechanical durability of water electrolysis membranes and the implications of reducing their thickness. Similarly, chemical degradation, as measured by fluoride release rate, has been reported by several groups; however, a clear picture of the nature of this degradation mechanism remains unclear, and the similarities (and differences) with fuel cells are not fully understood. Strategies successfully applied to the fuel cell field, such as reinforcing fibers or stabilizing additives, are likely to play an important role in electrolysis membranes. In addition, fluorine-free membranes are becoming an increasingly important topic of research due to the growing concerns with fluorocarbon safety and potential regulations. Hydrocarbon membranes offer a potential alternative and have advantages in gas crossover compared to PFSAs. However, there remain many performance, durability, and cost barriers with these materials as well.

### 9.3 Porous Transport Layer

The interface between the PTL and the CL has been shown to be the most important parameter in the design of PTLs. This interface can be tailored by the addition of an MPL, laser sintering, or through other surface modification techniques. Low loading CLs are possible with good interfacial contact to the CL, as MPL can help with in-plane electron transport. Fabrication methods still primarily rely on high-temperature sintering of Ti powder or fibers; however, alternative methods based on spark plasma sintering and high-velocity flame spraying are emerging. Coatings are crucial in preventing Ti passivation and reducing contact resistance. While Pt is still the state-of-the-art, various groups have been studying alternatives and lower-cost materials compared to Pt. Ti currently represents a high cost for the stack, and SS material may become a possibility if an ideal coating is developed for the PTL.

### 9.4 Bipolar Plate

BPPs are indispensable components of PEM water electrolyzers, providing mechanical support, electrical conduction, and channels for fluid and thermal management. Designing efficient, long-lasting BPPs requires balancing corrosion resistance, electrical/thermal conductivity, mechanical strength, and ICR in harsh electrochemical environments. While Ti remains the benchmark material due to its corrosion resistance, its high cost and processing difficulty have encouraged research into alternatives like SS, Ni alloys, and carbon-based composites. Surface treatments such as conductive ceramic coatings and precious-metal layers are vital in minimizing degradation and ensuring low interfacial resistance. Additionally, innovations in flow-field designs (e.g., interdigitated and serpentine structures) directly influence reactant transport and bubble removal. The continued development of cost-effective, high-performance BPPs will depend on the co-optimization of base materials, coating technologies, and scalable manufacturing methods tailored to long-term PEMWE operation.

### 9.5 Gasket

Gaskets are an essential, multifunctional component in PEMWE stacks. Their influence on stack performance and longevity is mediated through sealing efficiency, electrical isolation, chemical stability, and mechanical integrity. Advances in material science (e.g., nanocomposites), computational modeling, and smart diagnostics are progressively transforming gasket design from a static sealing solution to a dynamic, performance-critical element. As PEMWE systems scale for green hydrogen production, reliable, high-durability gaskets will be indispensable in ensuring safe, efficient, and long-term electrolyzer operation.

### 9.6 Modeling

To date, the majority of PEMWE computational models have addressed only a subset of the physical processes required to predict performance, electrochemical impedance (EIS) and durability; however, they have already provided critical insights. For example, they have highlighted the importance of charge transport and that, in most instances, mass transport losses might not be limiting PEMWE performance. To improve beginning of life performance predictions, microkinetic models are starting to be introduced that account for activity changes with catalyst oxidation state. A more detailed treatment of the PTL/CL interface would also result in improved predictions, but it will require the introduction of PTL pore-level features into commonly used volume-averaged MEA models. In many instances, it is the trade-off between performance and crossover that dictates the cell design; therefore, crossover models are now starting to be developed. Achieving an accurate model will require accurately estimating the movement of liquid water in the PEM, and the concentration of dissolved oxygen and hydrogen at the PEM/CL interface. Research has also started on developing experimental setups to estimate individual model input parameters, such as CL conductivities; however, additional work is needed in this area. Validation studies are starting to appear, but need to be extended to include a comparison of not only polarization curves, but also high-frequency resistance, water fluxes across the membrane, and, by means of reference electrodes, anodic and cathodic overpotential. For EIS analysis, PEMWE models have to be made transient. This would be a first step toward developing degradation models, which, as we have discussed, are still in their infancy and are necessary to reduce costly and time-consuming experimental testing. Durability models should account for both mechanical and chemical membrane degradation, as well as catalyst dissolution.

### 9.7 Characterization

Table 7 summarizes the key information, advantages, limitations, and the specific components that can be analyzed by each method. Each characterization method provides unique insights, but no single technique can comprehensively assess structure and composition across the bulk, surface, and interfaces of a sample. Given the complexity of PEMWE systems, a multitechnique approach is essential to evaluate both pristine and degraded components, as well as individual and integrated components. For integrated components, understanding interfacial regions is critical, as these are the primary sites of electrochemical activity and degradation. Integrating information from multiple methods also ensures a holistic understanding of component interactions and enables optimization for improved performance and longevity. Characterizing integrated components comes with challenges, as it often involves extensive sample preparation, which increases the risk of artifacts and complicates data interpretation. These challenges are further amplified when analyzing degraded samples, where degradation patterns are often heterogeneous across both through-plane and in-plane directions. To enable meaningful cross-study comparisons and actionable insights, it is critical to report the exact sampling location within the cell or stack, including flow-field design and position. Future studies should focus on multiscale, spatially resolved analyses and clearly document where samples are taken, at a minimum, specifying their location within the MEA area. Such practices will significantly enhance the reliability, reproducibility, and comparability of degradation assessments across the field.

**Table 7:** Main Characterization Techniques Used in PEMWEs

technique	basics	capabilities	main use in PEMWE
XRD	identifies crystalline structures via diffraction patterns.	phase identification, crystallite size estimation, structural analysis.	confirms the structure of synthesized catalysts and evaluates bulk crystallinity.
TGA	measures material weight changes as a function of temperature or time.	thermal stability assessment, composition analysis, phase transitions.	evaluates material degradation and thermal properties.
ICP-MS	utilizes plasma ionization for trace elemental analysis.	high sensitivity detection of dissolved elements, multielement analysis.	tracks catalyst degradation and metal leaching (e.g., Ir, Pt, Ti dissolution).
XAS	determines element-specific electronic and geometric structure.	identifies oxidation states, local atomic arrangements, bond distances.	studies catalyst oxidation states, coordination environments, and structural evolution.
XPS	surface-sensitive technique that analyzes chemical states of elements.	measures oxidation states, elemental composition, and bonding environments.	examines surface chemistry, catalyst oxidation states, and degradation mechanisms.
SEM-EDS	uses an electron beam to generate high-resolution images and elemental spectra.	surface morphology imaging, elemental composition mapping.	characterizes PTL and CL structure, uniformity, and degradation effects.
XCT	non-intrusive 3D imaging technique for structural analysis.	high-resolution imaging, operando studies, water distribution analysis.	investigates PTL porosity, CL distribution, and mass transport properties.
AFM	measures surface morphology and material properties at the nanoscale.	surface roughness, stiffness, adhesion, chemical properties analysis.	examines CCM and PEM surface properties, including hydrophobicity and conductivity.
TEM	high-resolution imaging of atomic-scale structures using electron transmission.	atomic-resolution imaging, crystallinity analysis, elemental composition mapping.	analyzes nanostructured catalysts, particle dispersion, and defect structures.
ToF-SIMS	surface-sensitive technique using ion beam sputtering.	high lateral/mass resolution, elemental/molecular mapping, depth profiling.	studies catalyst-ionomer interactions, surface composition, and contaminant detection.

## Nomenclature

**Symbols**
*A*_v_: specific surface area/m^–1^
*C*_dl_: capacitive coefficient/A s V^–1^ m^–3^
*C*_p_: specific heat at constant pressure/J kg^–1^ K^–1^
*c*_*i*_: molar concentration of species *i*/mol m^–3^
*D*: mass diffusivity/m^2^ s^–1^
*E*_eq_: equilibrium potential/V
EW: electrolyte equivalent weight/kg mol^–1^
*F*: Faraday constant (96485.333 C mol^–1^)
***F***: volumetric force/N m^–3^
*f*_*k*_: fraction of mode *k*/–
***f***_S_: force per unit area/N m^–2^
Δ*G*: activation energy/J
*g*: kinetics parameter/–
*I*: surface current density/A m^–2^
***i***_p_: ionic current density vector/A m^–2^ s^–1^
*J*(*s*): Leverett J-function/–
*j*: superficial or volumetric current density/A m^–2^, A m^–3^
***K***: permeability tensor/m^2^
*k*: thermal conductivity, Boltzmann constant (1.38 × 10^–23^ J K^–1^)/W m^–1^ K^–1^
*m*: van Genuchten’s parameter/–
*N*_H_2__: H_2_ crossover flux/mol m^–2^ s^–1^
*n*: van Genuchten’s parameter/–
*n*_d_: electro-osmotic drag coefficient/–
***n***_S_: unit normal vector/–
*p*: pressure/Pa
*p*_c_: capillary pressure/Pa
*p*_cb_: van Genuchten’s parameter/Pa
*R*: universal gas constant (8.314 J K^–1^ mol^–1^)
*r*: radius/m
*S*_*i*_: source term/SI
*s*: liquid saturation/–
*s*_*k*_: standard deviation of mode *k*/SI
*T*: temperature/K
*t*: time/s
***u***: velocity vector/m s^–1^
***v***: crossover velocity vector/m s^–1^
*x*: primary in-plane coordinate/m
*y*: through-plane coordinate/m
*z*: secondary in-plane coordinate/m
**Greek letters**
α: transfer coefficient or scalar function/–
β: Forchheimer inertial coefficient/m^–1^
δ_S_: Dirac delta function/–
ε_V_: porosity/–
ε_m_: ionomer volume fraction/–
θ: contact angle/–
**κ**: curvature/m^–1^
λ: electrolyte water content/–
μ: dynamic viscosity/kg m^–1^ s^–1^
ρ: density/kg m^–3^
σ: surface tension coefficient/N m^–1^
**σ**: conductivity tensor/S m^–1^
**τ̂**: stress tensor/Pa
ϕ: electrical or protonic potential/V
**Subscripts and superscripts**
avg: average
c: characteristic
ECSA: electrochemically active surface area
e: electrical
eq: equivalent
f: fluid
g: gas
ion: ionomer
l: liquid
m: membrane phase
ox: oxidant species
p: protonic
REV: representative elementary volume
red: reduced species
s: solid
